# ACVRL1 drives resistance to multitarget tyrosine kinase inhibitors in colorectal cancer by promoting USP15-mediated GPX2 stabilization

**DOI:** 10.1186/s12916-023-03066-4

**Published:** 2023-09-25

**Authors:** Xiaolin Lu, Ruiqi Liu, Yuanyu Liao, Luying Cui, Haoxiu Sun, Dongzhi Zhang, Bojun Wang, Lin Fang, Xin Guan, Yuanfei Yao, Chao Liu, Yanqiao Zhang

**Affiliations:** 1 Department of Gastrointestinal Medical Oncology, Harbin Medical University Cancer Hospital, Harbin, China; 2 Key Laboratory of Tumor Immunology in Heilongjiang, Harbin, China; 3 China Clinical Research Center for Colorectal Cancer in Heilongjiang, Harbin, China; 4 Department of Orthopedic Surgery, Harbin Medical University Cancer Hospital, Harbin, China; 5 Department of Radiation Oncology, Sun Yat-sen University Cancer Center, Guangzhou, China; 6 School of Life Science and Technology, Harbin Institute of Technology, Harbin, China; 7 Department of Neurosurgery, Harbin Medical University Cancer Hospital, Harbin, China; 8 Translational Medicine Research and Cooperation Center of Northern China, Heilongjiang Academy of Medical Sciences, Harbin, China

**Keywords:** Colorectal cancer, Multitarget tyrosine kinase inhibitors, Drug resistance, Activin A receptor-like type 1, Glutathione peroxidase 2, Reactive oxygen species

## Abstract

**Background:**

Multitarget tyrosine kinase inhibitors (mTKIs) such as Regorafenib and Sorafenib have already been approved for the treatment of many solid tumours. However, the efficacy of mTKIs in colorectal cancer (CRC) is limited; the underlined mechanism remains largely elusive. Our study was aimed to find out the resistance mechanism of mTKIs in CRC.

**Methods:**

RNA sequencing was used to identify the expression of Activin A receptor-like type 1 (ACVRL1) under the treatment of mTKIs. Gain/loss-of-function experiments were performed to assess the biological function of ACVRL1 in resistance to mTKIs. The underlying mechanisms of ACVRL1-mediated mTKI resistance were investigated by using liquid chromatography-mass spectrometry assays (LC-MS), co-immunoprecipitation assays (Co-IP), chromatin immunoprecipitation assays, ubiquitination assays, dual luciferase reporter assays, etc.

**Results:**

RNA sequencing identified the activation of ACVRL1 under the treatment of mTKIs in CRC cells. ACVRL1 knockdown and overexpression significantly affects the sensitivity of CRC cells to mTKIs both in vitro and vivo. Mechanistically, we found the β-catenin/TCF-1-KCNQ1OT1/miR-7-5p axis mediated the activation of ACVRL1.

Furthermore, LC-MS assays indicated the interaction between ACVRL1 and glutathione peroxidase 2(GPX2) protein. IP assay defined ACVRL1 truncation (282–503aa) could be responsible for interacting with GPX2, and rescue experiments with ACVRL1 truncations confirmed the importance of this interaction in driving mTKI resistance. Co-IP assays confirmed that ACVRL1 associates with ubiquitin-specific peptidase 15(USP15) which directly deubiquinates GPX2 at the K187(K, lysine) site, leading to the accumulation of GPX2 protein. Rescue experiments performed with the lysine mutants in GPX2 CRISPR knockout cell model confirmed the importance of GPX2 K187 mutant. As a result, the increased ROS clearance and decreased cell apoptosis eventually lead to mTKI resistance in CRC.

**Conclusions:**

Our results demonstrate that the Wnt/β-catenin/KCNQ1OT1/miR-7-5p/ACVRL1/GPX2 biological axis plays a vital role in CRC, targeting which may be an effective approach for overcoming mTKI resistance.

**Supplementary Information:**

The online version contains supplementary material available at 10.1186/s12916-023-03066-4.

## Background

Colorectal cancer (CRC) is the third most common type of cancer [1]. However, as the disease progresses, approximately 50% of CRC patients will eventually develop metastatic CRC (mCRC), which has a 5-year survival rate of less than 20% [2, 3]. Owing to the broad anti-tumour spectrum, multitarget tyrosine kinase inhibitors (mTKIs), such as Regorafenib and Sorafenib, are considered as potential target drugs for various solid tumours [4]. However, the clinical performance of mTKIs in CRC is suboptimal. In the phase III CORRECT trial, Regorafenib improved the overall survival of patients by 1.4 months and the objective response rate (ORR) of Regorafenib was only 1.0% [5]. Several phase I/II clinical studies have observed that a small number of CRC patients treated with Sorafenib had stable disease, but no objective response was obtained [6]. The overall clinical response of mTKIs in CRC is limited, and the underlying mechanism of this poor response still needs to be further explored.

ACVRL1 is a type I receptor for TGF-β family ligands BMP9 and BMP10 and important regulator of normal blood vessel development [7]. Owing to its critical role in angiogenesis, ACVRL1 has gained attention as a therapeutic target in cancer. Pharmacological targeting ACVRL1 by using anti-ACVRL1 antibody or a ligand-trapping agent can inhibit tumour growth and metastasis in preclinical models [8–10]. Despite the increased knowledge about the role of ACVRL1 in endothelial cell biology and angiogenesis, few studies have reported the role and therapeutic value of ACVRL1 in CRC cells. Furthermore, the downstream effectors of ACVRL1 remain largely elusive, the roles of ACVRL1 in tumourigenesis and drug resistance need to be further explored.

In this study, we found that ACVRL1 is highly expressed in CRC and acts as a key driver of mTKI resistance. Knockdown of ACVRL1 significantly increases the sensitivity to mTKIs both in vitro and vivo. Mechanistically, ACVRL1 is upregulated by the activation of β-catenin/TCF-1-KCNQ1OT1/miR-7-5p axis in CRC. Further, ACVRL1 stabilizes GPX2 via the interaction with deubiquitination enzyme-USP15, then suppressed the intracellular ROS and apoptosis, eventually leading to the mTKI resistance. Our study provides novel insights into the molecular mechanisms of resistance to mTKIs in CRC. Targeting ACVRL1 has the potential to be an effective strategy for overcoming the mTKI resistance in CRC.

## Methods

### Cell culture and gene knockout

CRC cell lines were purchased from ATCC (American type culture collection, USA). HCT116, LS174T, and HCT15 cells were respectively cultured in McCoys-5A, MEM, and DMEM media (Gibco, USA) supplemented with 10% FBS, glutamine, and penicillin/streptomycin at 37°C in 5% CO_2_. All cell lines were validated by STR DNA fingerprinting. Experiments were carried out within 6 months after acquisition of the cell lines. In addition, we ruled out mycoplasma contamination using a PCR-based method.

For CRISPR–Cas9 gene knockout, the human LentiCRISPR v2-GPX2 sgRNAs were purchased from Synbio Technologies. The sgRNA sequences were as follows: GAGCTGGGTGAAGTCCCGGG (#1), AGCCACATTCTCAATCAGCA(#2),GAGCTTGGGATCGGTCATGA(#3), CTAGGAGAACTGTCAGAATG(#4). Briefly, the HCT15 cells were co-transfected with LentiCRISPR v2-GPX2 sgRNAs plasmid and GFP plasmid. Forty-eight hours later, GFP-positive cells were isolated by FACS and seeded at one cell per 96-well. Cells were grown in DMEM supplemented with 10% FBS. Single-cell clones were expanded and depletion of GPX2 was confirmed by western blot.

### Clinical samples

CRC and adjacent normal tissues used in this study were obtained from CRC patients who underwent surgery between 2013 and 2019 at the Harbin Medical University Cancer Hospital. All samples were pathologically confirmed as CRC. This study was approved by the hospital’s Protection of Human Subjects Committee. Patients have signed informed consent for the use of tissue samples.

### Reagents and antibodies

The detailed information of reagents and antibodies included in this study was provided in Additional file 2: Table S6.

### Plasmids, lentiviral, microRNA, and lncRNA transfections

Plasmids, lentiviral, and siRNAs were purchased from WZ Biosciences Inc. (JiNan, China) or GENERAL BIOL (AnHui, China) (Additional file 2: Table S7-8). Synthetic hsa-miR-7-5p mimic and inhibitor, KCNQ1OT1 (human) siRNA and their negative control oligonucleotides were all purchased from GENERAL BIOL (AnHui, China). For transfection, CRC cells were plated in culture dish at a density of 1×10^5^ cells/well for 24 h. The usage of transfection reagent (Invitrogen, USA) is according to the manufacturer’s instructions. After 48h of transfection, cells were harvested, and the expression of target gene were confirmed by western blot and qRT-PCR.

### Drug treatment

The pharmacological concentrations of mTKIs and ML347 are as follows: HCT116 (Regorafenib, 5µmol. Sorafenib, 3µmol), HCT15 and LS174T (Regorafenib, 10µmol. Sorafenib, 5µmol), HCT15 (ML347, 25µmol). After indicated time of drug treatment, the cells were prepared for biological studies.

### RNA sequencing (RNA-seq)

HCT15 and LS174T cells were treated with DMSO, Regorafenib and Sorafenib for 24 h, then the total RNA was extracted. The RNA-seq was performed by Novogene Technology (Beijing, China). Each experiment was repeated in triplicate. RNA-seq data quality was checked using FASTQC and analysed using the TopHat-Cufflinks pipeline.

### Immunoprecipitation (IP)

The cells were transfected for 48 h, then lysed in NP-40 lysis buffer (P0013F, Beyotime Biotechnology) supplemented with protease inhibitors (Roche Applied Science, USA) for 30 min at 4°C on a low‐speed rotating shaker. The lysate was then centrifuged at 12,000 rpm 4°C for 10 min. Next, approximately 500 μL total volume of primary antibody was added and was shaken on rotating shaker at 4°C for overnight. Immune complexes were collected by adding 40 µL of Protein A/G PLUS-Agarose (sc-2003, SantaCruz Biotechnology, USA) for 6 h at 4°C. Immunoprecipitates were collected by centrifugation at 2500 rpm for 5 min at 4°C. Collect the supernatant to proceed with the analysis.

### Liquid chromatography-tandem mass spectrometry (LC-MS)

To analyse ACVRL1 interacting proteins, HCT116 cells were transfected with ACVRL1-Flag or vector-Flag(pENTER) and subjected to IP assays with anti-DYKDDDDK magnetic agarose (Thermo, USA). After washing five times with PBS buffer, samples were boiled in 2×SDS loading buffer, resolved in SDS-PAGE, visualized by Coomassie Blue staining, and subjected to LC-MS analysis (Thermo Scientific IMQ Exactive Plus, USA). Simultaneously, we stained the gel using Silver Stain Kit (CoWin Biosciences, China) after SDS-PAGE finished. The staining assays were carried out as kit instructions. Compared with the control group, peptides with a ratio of more than 2 were more likely to interact with ACVRL1 in the mass spectrometry data.

### Ubiquitination assay

Cells were transfected with indicated plasmids. Forty-eight hours after transfection, the cells were treated with 20 µM MG132 (MedChemExpress, USA) for 4 h before harvesting. Cell extracts were lysed in NP-40 Lysis Buffer supplemented with protease inhibitor, and the lysates were incubated with GPX2 or HA tag antibody at 4°C overnight. Immune complexes were collected by adding 40 µL of Protein A/G PLUS-Agarose (sc-2003, SantaCruz Biotechnology, USA) for 6 h at 4°C. Immunoprecipitates were collected by centrifugation at 2500 rpm for 5 min at 4°C and analysed by western blot.

### Protein half-life assay

Cells were treated with CHX (10 μM)(MedChemExpress, USA) for various periods to block protein synthesis. Crude extracts were prepared, and protein expression was assessed by western blot analysis.

### Chromatin immunoprecipitation assay (CHIP)

HCT15 and LS174T cells (3×10^6^) were seeded in 100-mm dishes, and after 1% formaldehyde treatment, the cells were lysed by radioimmunoprecipitation assay (RIPA) lysis buffer. Genomic DNA was isolated and sheared into 200–600 bp fragments by the sonicator. After centrifugation, the supernatants were taken and chromatin was incubated and precipitated with CTNNB1 antibodies (ab32572)(1:150, abcam,USA) or IgG (Beyotime Biotechnology, Shanghai, China) at 4°C overnight. The immune complexes were then precipitated using protein A/G-Sepharose beads (GE Healthcare, Chicago, USA) for 4 h. Next, the immune complexes were washed with different washing buffers, followed by a low-salt washing buffer, high-salt washing buffer, LiCl washing buffer, and TE buffer. The immune precipitates were eluted using elution buffer and reversal of crosslinking at 65°C overnight, and KCNQ1OT1 promoter primers (F: GTTTGAACACGGTCAGCACG; R:CAGCCCAC TCTGAACCACC) were used to amplify the binding sites for β-catenin. DNA was finally eluted as per the manufacturer’s protocol and analysed by qRT-PCR. Primers are summarized in Supplementary Materials and Methods.

### Dual luciferase reporter assay

The modified reporter vectors used in this assay were all constructed by GENERAL BIOL (China). Cells were seeded in 24-well plates and co-transfected with ACVRL1 3′-UTR reporter plasmids (wild-type or mutant) and miR-7-5p mimics. The KCNQ1OT1 promoter (Kp2022) and KCNQ1OT1-binding-mut promoter (Kp1080) in which the TCF-1-binding site was mutated were cloned into pGL3-Basic vector (Promega), and the TCF-1 overexpression and control CRC cells were transfected with the above modified vector. KCNQ1OT1 gene and KCNQ1OT1-mut sequence were cloned into pmirGLO vector (Promega). Renilla luciferase (pRL-TK) (Beyotime Biotechnology, China) was used as a transfection control. Luciferase activity was measured using the dual luciferase reporter assay system (Promega, USA). Relative promoter activity was calculated as firefly luminescence/Renilla luminescence.

### Animal experiments

Six-week-old female nude BALB/C mice (Vital River Laboratories, China) were subcutaneously injected with HCT15 cells transfected with shACVRL1 or shNTC Lentivirus. When tumour volumes reached approximately 0.2 cm^3^, the mice were treated with 30 mg/kg Regorafenib or Sorafenib daily (orally) for 3 weeks.

B-NSG mice (Biocytogen, China) were used to establish patient-derived xenograft (PDX) models, as described in our previous study [11]. Tumour fragments were cut into pieces 2–3 mm in diameter and inoculated subcutaneously into the right flank of NSG mice. When the tumours reached approximately 0.2 cm^3^, the mice were randomly allocated to the treatment groups. Mice were treated with Regorafenib orally (30 mg/kg, daily), ML347 intraperitoneally (45 mg/kg, every other day), Regorafenib combined with ML347, or vehicle (PBS).

Body weights and tumour volumes were measured every other day. Tumour volume was calculated using the formula (length × width^2^)/2, and all experiments were performed according to the official recommendations of the Chinese animal community.

### Statistical analysis

All statistics are expressed as mean ± SD and were analysed using SAS 9.3 (SAS Institute Inc., USA). All experiments were conducted at least thrice. ANOVA and Student’s *t* test were performed to compare the differences among the experimental groups. Statistical significance was set at *P* < 0.05.

## Results

### ACVRL1 acts as a critical gene for mTKI resistance in CRC

**Fig. 1:**
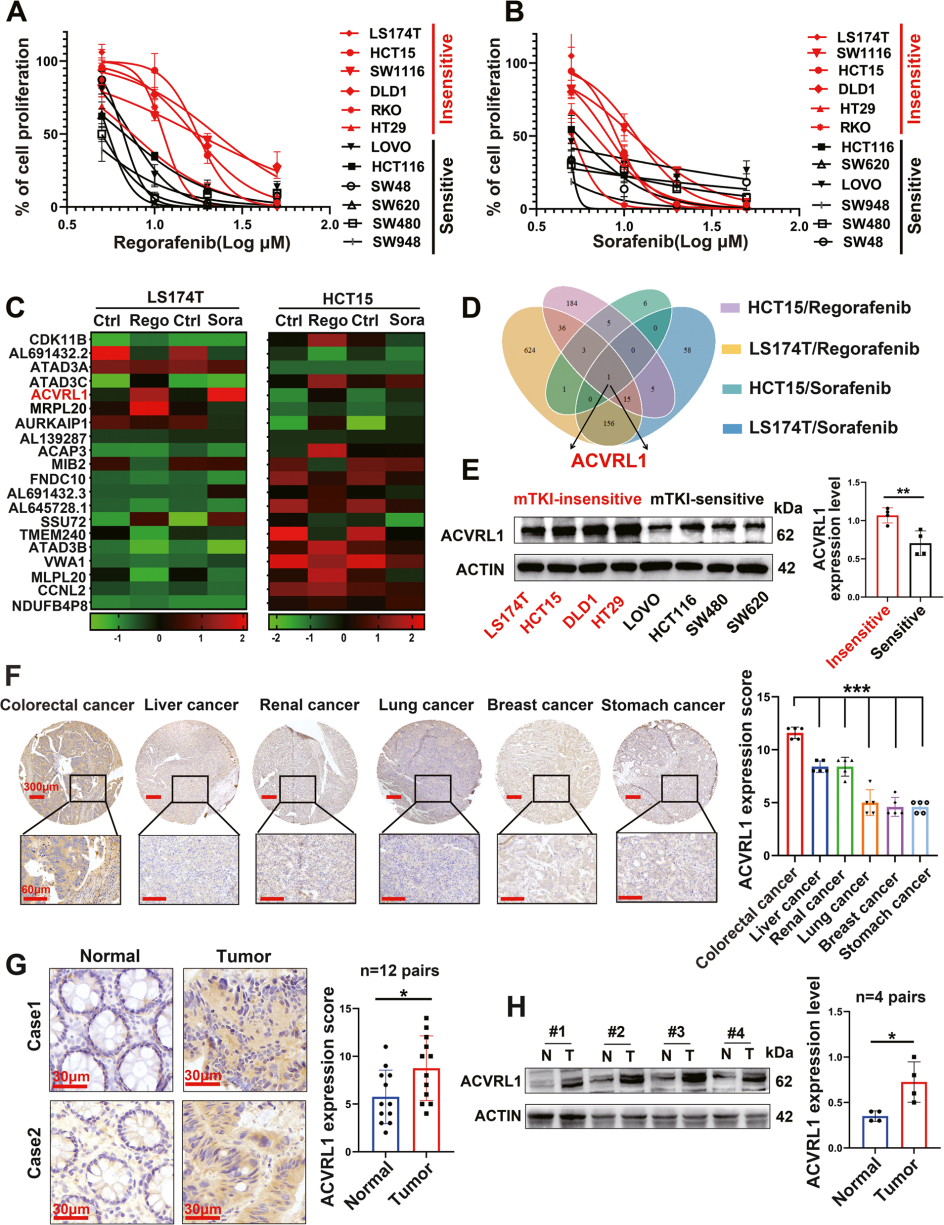
RNA-seq identified ACVRL1 as a critical gene for mTKI resistance in CRC. **A, B** CRC cells were treated with different concentrations of Regorafenib and Sorafenib for 48 h and evaluated for proliferation by CCK8. **C** The heatmap of RNA-seq revealed the upregulation of ACVRL mRNA of both HCT15 and LS174T cells pretreated with Regorafenib or Sorafenib. **D** The Venn diagram shows ACVRL1 is the only gene upregulated in four experimental groups (HCT15-Reg/Sora, LS174T-Reg/Sora). **E** The expression levels of ACVRL1 in CRC cell lines. **F** The expression of ACVRL1 was detected in multi-cancerous tissue microarray using IHC testing. **G,H** The expression of ACVRL1 in CRC and adjacent normal tissues was detected by IHC and western blot. The error bar represents the mean with upper and lower limits. All data are presented as the mean ± SD. Experiments were repeated three times. (Student’s *t* test *P < 0.05, **P < 0.01,***P < 0.001, ****P < 0.0001)

We selected Regorafenib and Sorafenib to explore the general mechanism of resistance to mTKIs. CRC cell lines were treated with Regorafenib or Sorafenib for 48 h to examine drug sensitivity. We selected the median IC50 value of TKIs to distinguish sensitive and insensitive cells and found that the insensitive cells were highly overlapping in different TKIs treated groups (IC50 values are presented in Additional file 2: Table S1-2). CRC cells could be basicly divided into mTKI-sensitive (black curves) and mTKI-insensitive (red curves) cells (Fig. 1A, B). HCT15 and LS174T cells, which represent the most insensitive CRC cell lines to mTKIs, were selected for the subsequent experiments. RNA-seq was performed on LS174T and HCT15 cells after treatment with Regorafenib or Sorafenib. As shown in the heatmap and Venn diagram (Fig. 1C, D) (Additional file 4: RNA-seq data), ACVRL1 was the only upregulated gene in all four groups (LS174T-Rego/Sora and HCT15-Rego/Sora). Western blot analysis showed that the expression of ACVRL1 was higher in mTKI-insensitive cells (LS174T, HCT15, DLD1, and HT29) than in mTKI-sensitive cells (LOVO, HCT116, SW480, and SW620)(Fig. 1E). By performing IHC testing on multi-cancerous tissue microarray, we found that the expression of ACVRL1 is significantly higher in CRC when compared with most mTKI-sensitive tumours (HCC, RCC, etc.) (Fig. 1F). Analysis of the gene expression profile of ACVRL1 in the Human Protein Atlas database (HPA) [12] and The Cancer Genome Atlas Program (TCGA) [13] also confirmed the high expression of ACVRL1 in CRC (Additional file 1: Figure S1A, B). Furthermore, IHC and western blot showed that the expression of ACVRL1 was higher in CRC tissues than in the adjacent normal tissues (Fig. 1G, H) (Additional file 1: Figure S1C). These results together revealed that ACVRL1 is highly expressed in CRC and that there is a positive correlation between the expression of ACVRL1 and mTKI resistance. Therefore, ACVRL1 may act as a critical gene for mTKI resistance in CRC.

### Knockdown and overexpression of ACVRL1 significantly affects the sensitivity of CRC cells to both Regorafenib and Sorafenib

**Fig. 2:**
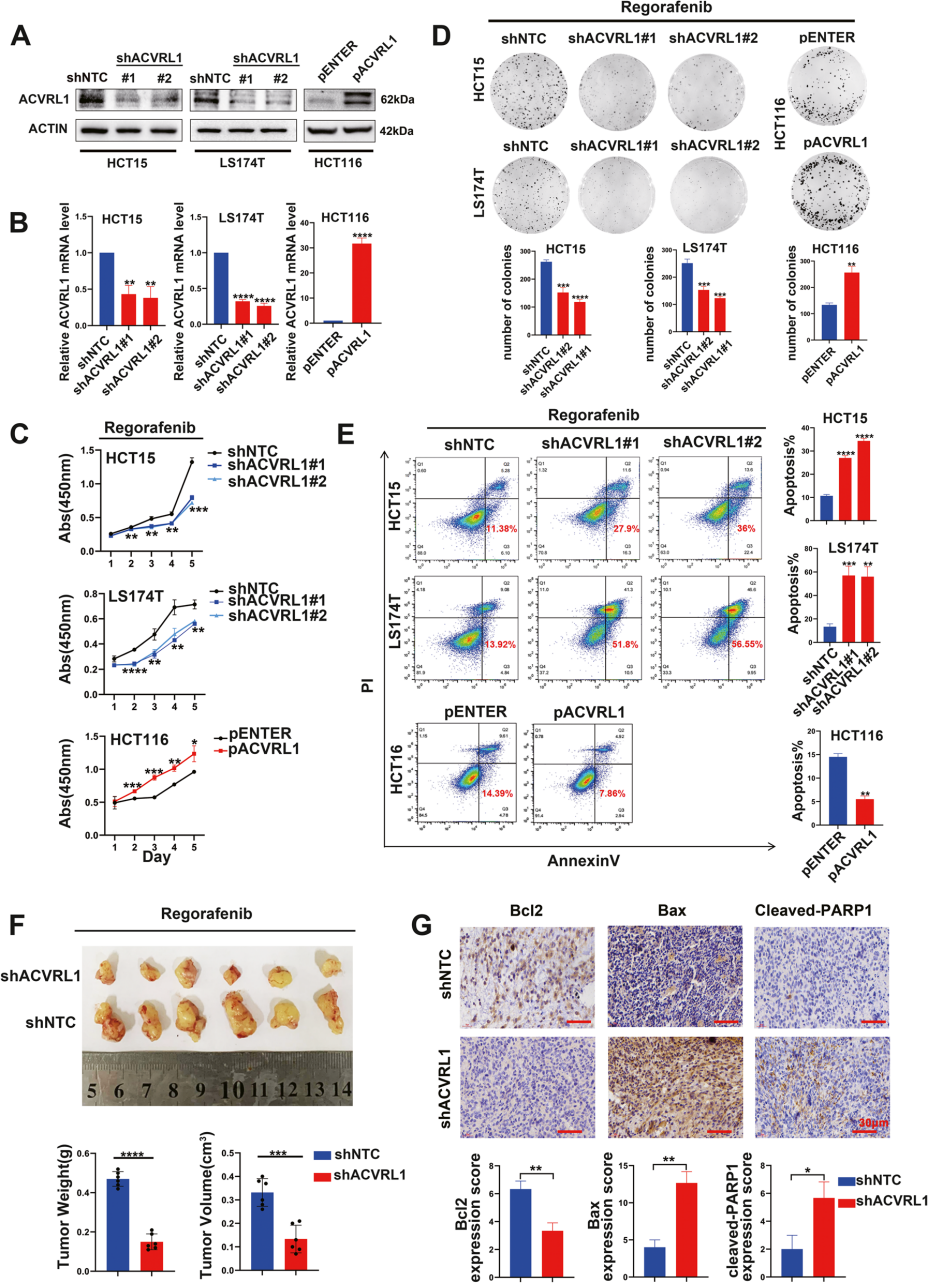
Knockdown and overexpression of ACVRL1 significantly affects the sensitivity of CRC cells to Regorafenib. **A, B** HCT15 and LS174T cells were transfected with shACVRL1 or shNTC, HCT116 cells were transfected with ACVRL1 overexpression plasmid (pACVRL1) or pENTER. The transfection efficiency was verified by western blot and qRT-PCR. **C, D** Survival rates of the transfected cells treated with Regorafenib were detected by CCK8 and Clonal formation assay. **E** Apoptosis level of the transfected cells treated with Regorafenib was detected by flow cytometry using AnnexinV/PI staining.** F** The knockdown of ACVRL1 by lentiviral-based shRNA approach sensitized CRC cells to Regorafenib in nude mice, with lower tumour weight and volume. **G** The representative tumour tissue sections from xenografts were analysed by IHC for the expression of apoptotic markers: Bcl-2, Bax, Cleaved-PARP1. Scale bar, 30 μm (*P < 0.05, **P < 0.01, ***P < 0.001, ****P < 0.0001)

To assess the effect of ACVRL1 on resistance to mTKIs (Regorafenib or Sorafenib), ACVRL1 was knocked down in mTKI-insensitive cells (HCT15 and LS174T) and overexpressed in mTKI-sensitive cells (HCT116) (Fig. 2A, B). Cells were treated with mTKIs (Regorafenib or Sorafenib) for indicated periods. CCK8 and colony formation assays showed that ACVRL1-knockdown cells had decreased survival rates after mTKI treatment when compared to control cells, whereas ACVRL1-overexpressing cells had increased survival rates when compared to that of control cells (Fig. 2C, D and Additional file 1: Figure S2A, B). Furthermore, knockdown of ACVRL1 promoted mTKI-induced apoptosis; however, overexpression of ACVRL1 inhibited mTKI-induced apoptosis (Fig. 2E and Additional file 1: Figure S2C). While without mTKI intervention, the expression of ACVRL1 only slightly affects the survival and apoptosis ratio of CRC cells (Additional file 1: Figure S6). In vivo experiments, the growth of tumours from ACVRL1-knockdown HCT15 cells was completely retarded after mTKI treatment, leading to lower tumour weight and volume than the control group (Fig. 2F and Additional file 1: Figure S2D). Consistent with the above results, IHC staining of the apoptotic markers in the xenograft tissues also indicated a higher apoptosis level in the shACVRL1 group **(**Fig. 2G). Meanwhile, interference with the expression of ACVRL1 had no significant effect on the DNA replication and differentiation of CRC cells under the treatment of mTKI (Additional file 1: Figure S7). Taken together, these findings suggest that the overexpression of ACVRL1 contributes to the resistance to mTKIs, whereas the inhibition of ACVRL1 significantly increases the sensitivity of CRC cells to mTKIs both in vitro and vivo.

### ACVRL1 interacts and positively correlates with GPX2 in CRC

**Fig. 3:**
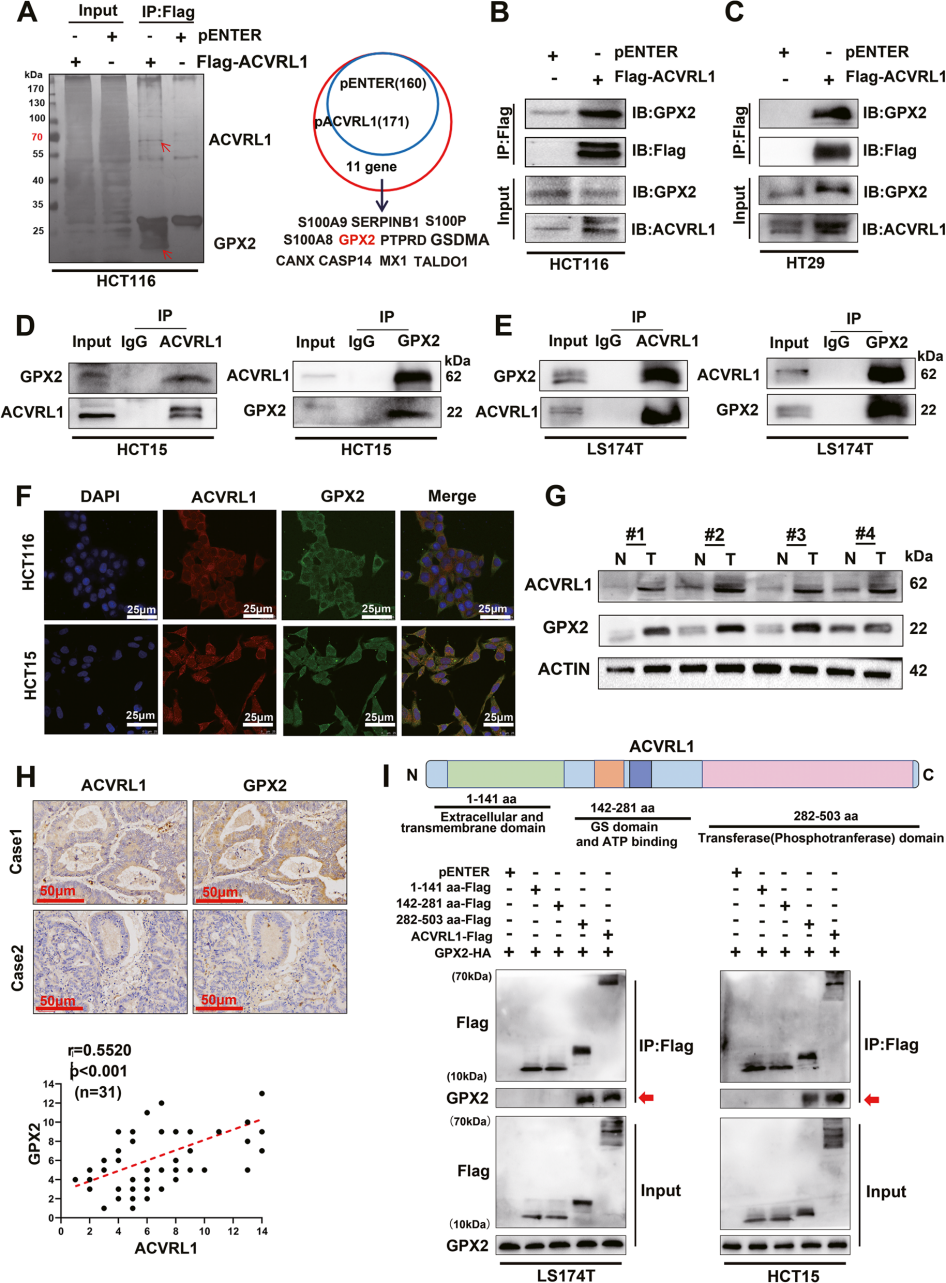
ACVRL1 interacts and positively correlates with GPX2 in CRC. **A** HCT116 cells were transfected with ACVRL1-Flag or pENTER(vector-Flag) and subjected to IP assays with anti-DYKDDDDK magnetic agarose. Lysates transfected with pENTER were used as a control. Bands specific to the Flag-ACVRL1 sample were excised and subjected to LS-MS analysis. Eleven genes were identified as candidates that might interact with ACVRL1 (right panel). The extracted proteins were then separated by SDS-PAGE and subjected to silver staining. A specific band, marked with an arrow, was identified as GPX2 protein in the ACVRL1-Flag group by LS-MS analysis. **B, C** Western blot showed that GPX2 was pulled down by anti-DYKDDDDK magnetic agarose in HCT116 and HT29 cells both of which were transfected with Flag-ACVRL1. **D, E** Co-IP assays conducted on HCT15 cells and LS174T cells revealed that GPX2 was enriched in ACVRL1-immunoprecipitated protein and ACVRL1 was also enriched in the GPX2-immunoprecipitated protein. IgG was used as a negative control. **F** IF was carried out to detect the localization of ACVRL1 and GPX2 in HCT15 and HCT116 cells, nucleus was labeled with DAPI (scale bar: 25 μm). **G** Protein level of ACVRL1 and GPX2 in four pairs of CRC tissues and their adjacent normal tissues were detected by western blot. **H** Representative images of IHC staining for ACVRL1 and GPX2 in CRC tissues (*P* < 0.01, *r*=0.5520). **I** Schematic diagram showing the domains of ACVRL1: extracellular and transmembrane domain, GS domain and ATP binding, transferase(phosphotransferase) domain. Full-length ACVRL1-Flag and Truncated ACVRL1(1-141aa, 142-281aa, 282-503aa)-Flag and GPX2-HA were co-transfected into LS174T and HCT15 cells. Lysates were analysed by IP of Flag with a subsequent western blot (*P < 0.05, **P < 0.01, ***P < 0.001, ****P < 0.0001)

To point out the underlying molecular mechanism of how ACVRL1 mediating mTKI resistance, LC-MS assays were performed on HCT116 cells transfected with ACVRL1-Flag or vector-Flag(pENTER). The lysates were subjected to IP assays with anti-DYKDDDDK magnetic agarose and then resolved by SDS-PAGE, followed by silver staining and mass spectrometry. The data showed 171 proteins in the pACVRL1 group and 160 proteins in the pENTER group (Additional file 5: LC-MS data). Eleven genes were identified as candidates that might interact with ACVRL1 (Fig. 3A). Among which, GPX2, which can neutralize H_2_O_2_ and reduce ROS accumulation, has been reported to play a critical role in maintaining the clonogenic and metastatic capacity of CRC [14]. Co-IP assays confirmed the exogenous and endogenous interaction between ACVRL1 and GPX2 in different CRC cell lines (HCT116, HT29, HCT15 and LS174T) (Fig. 3B–E). Immunofluorescence (IF) co-localisation assays revealed that ACVRL1 and GPX2 were co-localized in the cytoplasm of CRC cells (Fig. 3F). Western blot and IHC showed that both ACVRL1 and GPX2 levels were higher in human CRC tissues than in adjacent normal tissues, and there was a positive correlationship between ACVRL1 and GPX2 expression (Fig. 3G, H). To further define the domain of ACVRL1 that mediates the interaction with GPX2, we constructed ACVRL1 truncations (1–141aa, 142–281aa, 282–503aa) with Flag-tags based on the known functional domains. IP assays showed that the ACVRL1 truncations (282–503aa) could be responsible for interacting with GPX2 (Fig. 3I). These results together indicate that ACVRL1 can interact with GPX2, and there is a positive correlationship between the expressions of ACVRL1 and GPX2.

### ACVRL1 stabilizes GPX2 expression through USP15-mediated deubiquitination

**Fig. 4:**
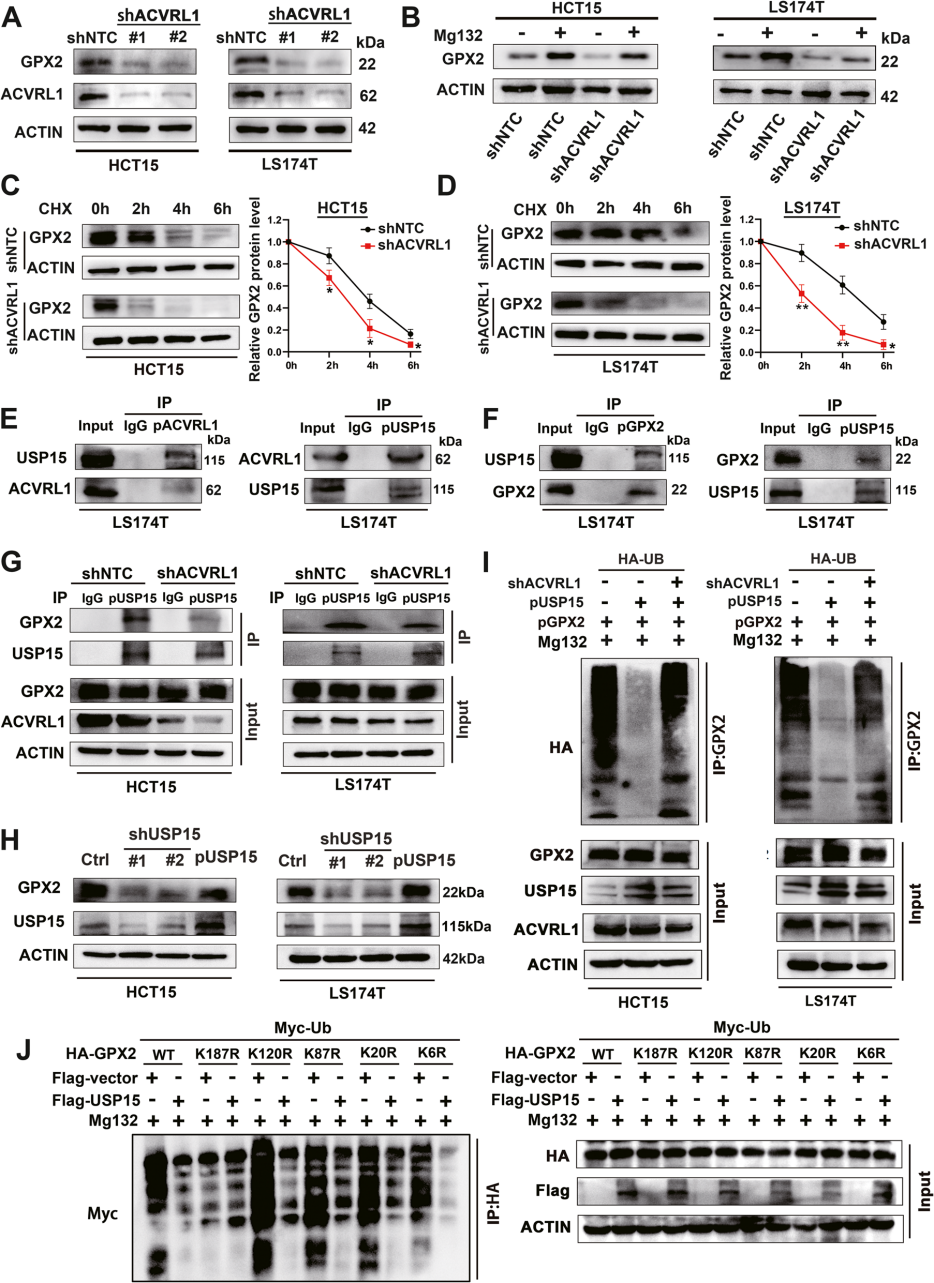
ACVRL1 stabilizes GPX2 expression through USP15-mediated deubiquitination.** A** The expression of GPX2 in ACVRL1 knocked down HCT15 and LS174T cells was detected by western blot. **B** CRC cells expressing control shRNA or ACVRL1 shRNA were incubated with MG132 (10 µM) for 4 h before western blot. MG132 stabilized GPX2 in shNTC group and rescued ACVRL1 silencing-induced GPX2 reduction. **C, D** ACVRL1 knockdown decreased the stability of GPX2 protein. CRC cells expressing control shRNA or ACVRL1 shRNA were incubated with CHX (10μM) followed by western blot. The quantified level of GPX2 protein was measured by ImageJ. **E** Co-IP assays conducted on LS174T cells revealed the endogenous interaction between ACVRL1 and USP15.** F** Co-IP assays revealed the endogenous interaction between USP15 and GPX2. **G** Western blot detected the expression level of GPX2 immunoprecipitated by USP15 in CRC cells transfected with shNTC or shACVRL1. **H** Depletion of USP15 decreased GPX2 expression and overexpression of USP15(pUSP15) stabilized GPX2 expression in CRC cells.** I** The ubiquitinated GPX2 protein were immunoprecipitated from CRC cells transfected with control vector or pUSP15 vector or both pUSP15 and shACVRL1 vector. Western blot detected the ubiquitinated level of GPX2. **J** Ubiquitination assays showed that USP15 deubiquitinates GPX2 at K187(K, lysine) site. Cells were transfected with mutant GPX2 protein with Flag-vector or Flag-USP15. The myc-Ub conjugated protein was pulled down by anti-HA antibody. The anti-myc antibody was used to detect the polyubiquitination level of GPX2 (*P < 0.05, **P < 0.01, ***P < 0.001, ****P < 0.0001)

The foregoing results indicated that ACVRL1 interacts with GPX2, but whether ACVRL1 regulates the expression of GPX2 is unknown. On further exploration, we found that knockdown of ACVRL1 did not affect the mRNA level of GPX2 (Additional file 1: Figure S3A), but significantly downregulated the protein level of GPX2 in both HCT15 and LS174T cells (Fig. 4A), which suggest that ACVRL1 may regulate the expression of GPX2 in the post-transcriptional level. IHC staining also indicated this regulatory effect of ACVRL1 on GPX2 in tumour xenografts (Additional file 1: Figure S4D). On the other hand, interference with GPX2 did not affect the expression of ACVRL1 (Additional file 1: Figure S3B)*.* CHX chase assay confirmed that ACVRL1 knockdown significantly accelerated the degradation rate of GPX2 protein (Fig. 4C, D). Thus we tend to further investigate the subcellular level at which ACVRL1 regulates GPX2 stability. Interestingly, ACVRL1 silencing-mediated downregulation of GPX2 could be rescued by the treatment with proteasome inhibitor MG132 (Fig. 4B). These results together suggest that ACVRL1 may interact with GPX2 to protect it from degradation via a proteasome-dependent mechanism. Previous studies have confirmed some DUBs, such as USP4, to have direct interaction with TβRI family [15]. Whether ACVRL1 affects the ubiquitination degradation of GPX2 through the interaction with some DUBs still need to be further explored. We overexpressed ACVRL1 and GPX2 separately in LS174T cells and then performed Co-IP studies followed by SDS-PAGE. Surprisingly, USP15 whose sequence and domain structure is high homologous with USP4 was identified as one of the most prominent interactors with both ACVRL1 and GPX2 (Fig. 4E, F). On further exploration, we found that knockdown of USP15 did not affect the interaction between ACVRL1 and GPX2 (Additional file 1: Figure S3C); similarly, knockdown of GPX2 also did not affect the interaction between ACVRL1 and USP15 (Additional file 1: Figure S3D), which indicated that ACVRL1 can directly interact with both GPX2 and USP15 independently. But when ACVRL1 was knocked down, the interaction between USP15 and GPX2 was significantly weakened (Fig. 4G), indicating that the interaction between GPX2 and USP15 is indirect which depends on the expression of ACVRL1. Besides that, we found that USP15 inhibition significantly decreased the protein level of GPX2, while overexpression of USP15 stabilized GPX2 in CRC cells (Fig. 4H). To further identify whether ACVRL1 stabilizes GPX2 through the deubiquitination mediated by USP15, we overexpressed both USP15 and HA-UB in CRC cells and then treated the cells with proteasome inhibitor MG132, the results showed USP15 overexpression significantly inhibited the ubiquitination of GPX2, which could be rescued by the depletion of ACVRL1 (Fig. 4I). The ubiquitination sites of GPX2 were predicted through the website [16], and then we constructed five lysine residue mutants of the GPX2 protein (GENERAL BIOL, China). Further studies indicated that the K187 (K, lysine) site of GPX2 was deubiquitinated by USP15 (Fig. 4J). These findings together suggest that ACVRL1 promotes the stability of GPX2 through the interaction with USP15 which directly mediates the deubiquitination of GPX2.

We further explored whether knockdown of ACVRL1 affects the intracellular ROS level. After transfection with shACVRL1 or shNTC plasmids, HCT15 and LS174T cells were cultured with DCFH-DA probes and then assessed by flow cytometry. The results showed that the intracellular ROS level significantly increased with ACVRL1 knockdown (Additional file 1: Figure S3E). These results indicate that ACVRL1 can interact with and stabilize GPX2, thereby reducing intracellular ROS level, which may result in mTKI resistance in CRC.

### ACVRL1 enhances resistance to mTKIs in a GPX2-dependent manner

**Fig. 5:**
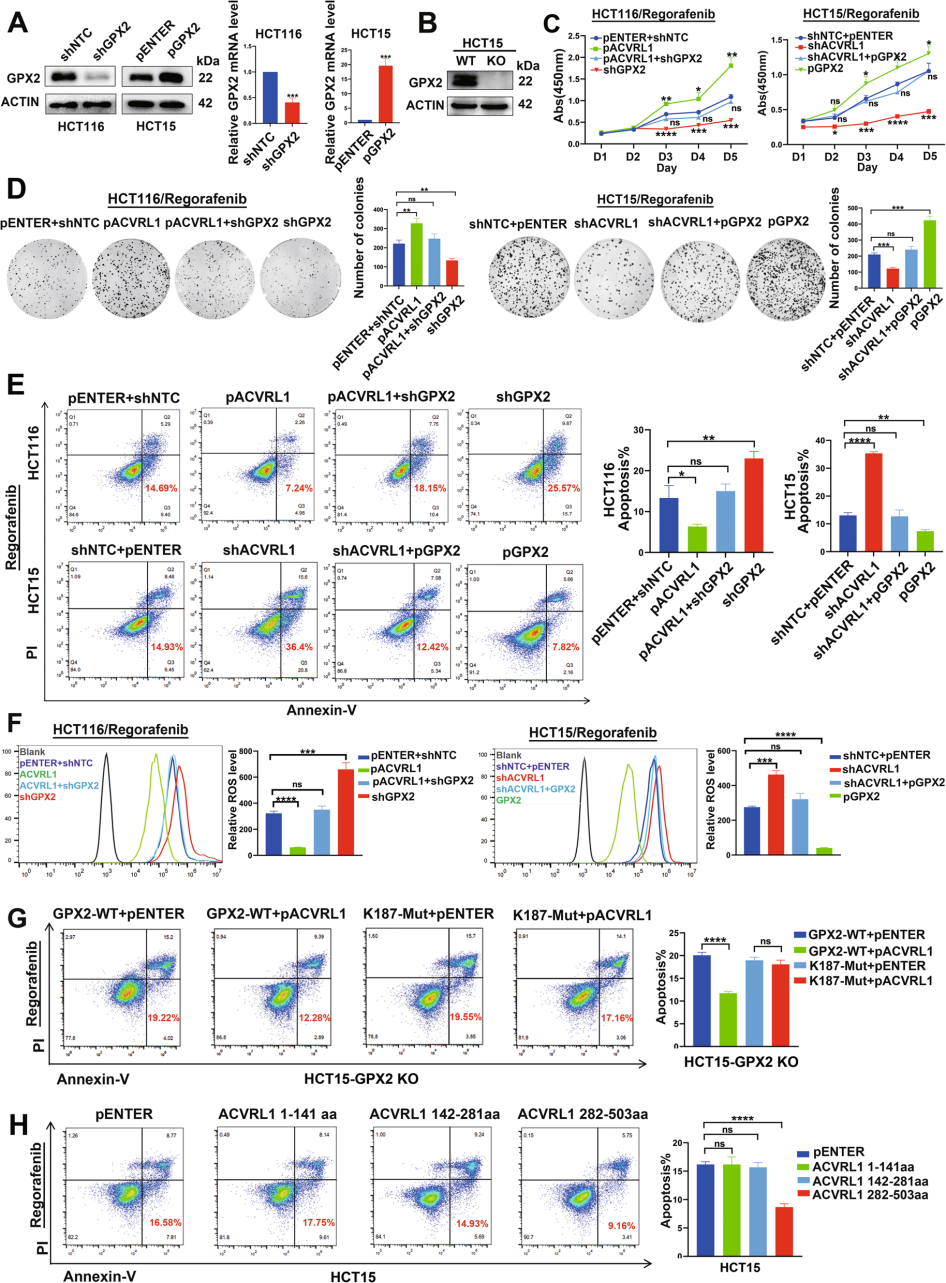
ACVRL1 enhances mTKI resistance in a GPX2-dependent manner.** A** GPX2 was knocked down in HCT116 with shGPX2 plasmid and overexpressed in HCT15 with GPX2 overexpression plasmid (pGPX2). The transfection efficiency was verified by western blot and qRT-PCR. **B** Western blotting analysis to determine the relative protein levels of GPX2 in HCT15-WT/KO cell lines. **C,D** CCK8 and clone formation assay were used to detect the survival ratio of different transfected groups. **E** The apoptosis levels of HCT116 and HCT15 cells transfected with indicated plasmids under Regorafenib treatment were detected by flow cytometry using AnnexinV/PI staining. **F** The intracellular ROS levels of HCT116 and HCT15 cells transfected with indicated plasmids under Regorafenib treatment were detected by flow cytometry. **G** HCT15-KO cells (HCT15 cells with GPX2-Knock out) were transfected with the following plasmids (GPX2-WT and pENTER, GPX2-WT and pACVRL1, GPX2-K187 Mut and pENTER, GPX2-K187 Mut and pACVRL1) and then treated with Regorafenib. The apoptosis levels were detected by flow cytometry using AnnexinV/PI staining. **H** HCT15 cells were transfected with pENTER or ACVRL1 truncations (1-141 aa, 142-281 aa, 282-503 aa) separately and then treated with Regorafenib. The apoptosis levels were detected by flow cytometry using AnnexinV/PI staining (ns means no significance. *P < 0.05, **P < 0.01, ***P < 0.001, ****P < 0.0001)

To further explore whether ACVRL1 directly mediates resistance to mTKIs through its interaction with GPX2, we performed rescue experiments by using HCT15 and HCT116 cells. The transfection efficiency of GPX2 overexpression plasmids and shGPX2 plasmids were verified by using western blot and qRT-PCR (Fig. 5A). Forty-eight hours after transfection with the indicated plasmids, CRC cells were treated with Regorafenib. CCK8 and colony formation assays confirmed that overexpression of ACVRL1 led to an increasement in the survival rate of CRC cells under mTKI treatment, while with GPX2 knockdown, this effect could partially be offset. Furthermore, upregulating GPX2 in HCT15 cells partially offset the growth inhibition mediated by ACVRL1-knockdown (Fig. 5C, D). The inhibition of apoptosis induced by ACVRL1 overexpression was relieved by GPX2-knockdown in HCT116 cells, while upregulating GPX2 in HCT15 cells could offset the apoptosis mediated by ACVRL1-knockdown (Fig. 5E). We then assessed the intracellular ROS level in HCT116 and HCT15 cells transfected with the indicated plasmids following treatment with Regorafenib. Knockdown of GPX2 or ACVRL1 both caused significant increasement of the intracellular ROS. ACVRL1 overexpression partially reduced the ROS generated by GPX2-knockdown in HCT116 cells, whereas ACVRL1-knockdown increased the ROS level in GPX2-overexpressing HCT15 cells (Fig. 5F).

Our previous findings have confirmed that ACVRL1 mediating the accumulation of GPX2 partly due to the deubiquitination of GPX2 at K187 site. To further assess the effect of K187 mutant of GPX2 on TKI resistance, we generated human CRC cells in which GPX2 had been knocked out by CRISPR/Cas9 (HCT15-KO) to perform the following rescue experiments. Western blot confirmed the knockout efficiency of GPX2 in HCT15-KO cells (Fig. 5B). Then the wild-type GPX2 (GPX2-WT) and GPX2 with K187 mutation (GPX2-K187 Mut) were independently transfected into HCT15-GPX2 KO cells with or without ACVRL1 overexpression. As shown in Fig. 5G, the apoptosis ratio of HCT15-GPX2 KO cells transfected with GPX2-WT and pACVRL1 is significantly reduced under the treatment of Regorafenib when compared with the control group. However, the apoptosis ratio of HCT15-GPX2 KO cells transfected with GPX2-K187 Mut and pACVRL1 showed no decreasement when compared with cells transfected with GPX2-K187 Mut and pENTER. The above findings indicate that the k187 site of GPX2 plays a critical role in ACVRL1 mediating mTKI resistance.

To further explore the role of the ‘complex’ of ACVRL1 and GPX2 in mediating mTKI resistance, we next performed rescue experiment by using ACVRL1 truncations. ACVRL1 truncations (1–141 aa, 142–281 aa, 282–503 aa) were separately transfected into HCT15 cells for 48h, then treated with Regorafenib for another 48 h. We found that the cells transfected with ACVRL1 (282–503 aa) which has been confirmed to be responsible for interacting with GPX2 (Fig. 3I) showed obvious resistance to the treatment of Regorafenib with lower percentage of apoptosis, while cells transfected with ACVRL1 (1–141 aa, 142–281 aa) did not show similar effect (Fig. 5H). The above experiments together demonstrate that ACVRL1 drives mTKI resistance in a GPX2-dependent manner and the interaction between the two proteins is crucial in this process.

### The biological axis of Wnt/β-catenin/TCF-1-KCNQ1OT1-miR-7-5p mediates ACVRL1 activation

**Fig. 6:**
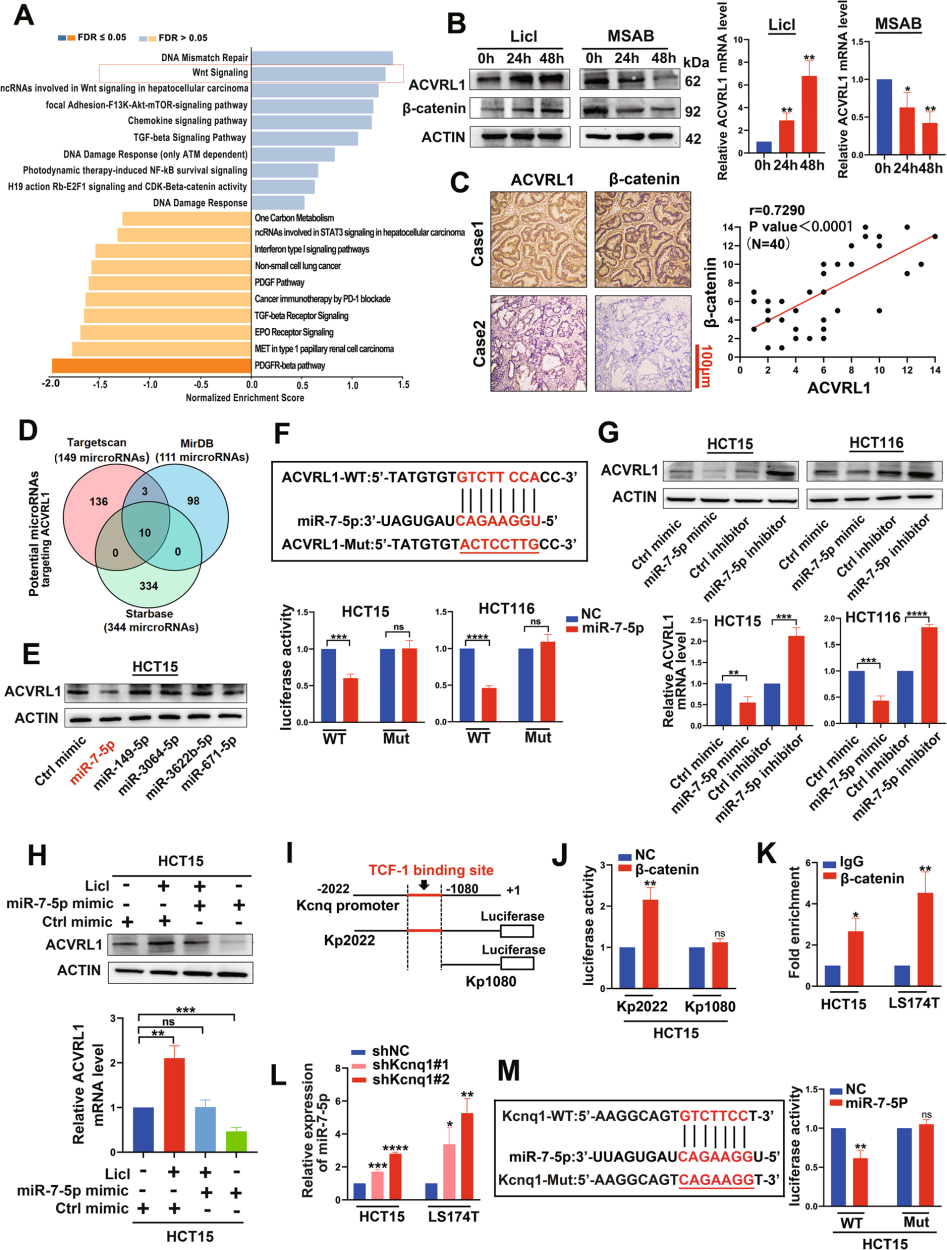
The biological axis of Wnt/β-catenin/TCF-1 -KCNQ1OT1-miR-7-5p mediates ACVRL1 activation. **A** KEGG pathway enrichment analysis revealed that the Wnt/β-catenin signalling was activated with mTKI pretreatment in RNA-seq. **B** The expression of both ACVRL1 and β-catenin under the treatment of Licl or MSAB was detected by western blot and qRT-PCR. **C** Representative images of IHC staining for ACVRL1 and β-catenin in CRC tissues (*P* < 0.0001, *r*=0.7290). **D** Three independent miRNA-target databases were used to predict the potential miRNAs targeted ACVRL1. **E** HCT15 cells were transfected with predicted miRNA mimics, among which only miR-7-5p could effectively inhibit the expression of ACVRL1. **F** Luciferase reporter activity was analysed after co-transfection of miR-7-5p mimic or negative control and WT ACVRL1 3’-UTR luciferase reporter construct or Mut construct into CRC cells.** G** The protein and mRNA levels of ACVRL1 were detected in CRC cells transfected with either miR-7-5p mimics or inhibitors. **H** HCT15 cells were transfected with miR-7-5p mimics or control, then treated with Licl or control. The expression of ACVRL1 was detected by western blot and qRT-PCR.** I** Schematic representation of the KCNQ1OT1 promoter region and the reporter constructs used for the luciferase assay. One reporter contains the KCNQ1OT1 promoter region from −294 to −2022 (Kp2022). The other reporter contains a truncated promoter in which the TCF-1 region was deleted (Kp1080). **J** The relative luciferase activity was detected in HCT15 cells co-transfected by Kp2022/Kp1080 and β-catenin. **K** The ChIP assay of β-catenin binding to the TCF site in the KCNQ1OT1 promoter in CRC cell lines. The assay was performed with or without β-catenin antibody. DNA was recovered from immunoprecipitated and nonimmunoprecipitated (input) chromatin and analysed by qRT-PCR. **L** The expression of miR-7-5p in CRC cells transfected by shKCNQ1OT1. **M** The predicted binding sites between miR-7-5p and KCNQ1OT1. The relative luciferase activities were detected in HCT15 cells transfected by KCNQ1OT1-WT and KCNQ1OT1-Mut (ns means no significance. *P < 0.05, **P < 0.01, ***P < 0.001, ****P < 0.0001)

We further investigated the mechanism underlying the 1upregulation of ACVRL1 in mTKI-pretreated cells. KEGG pathway enrichment analysis of the RNA-seq results revealed that Wnt/β-catenin signalling was activated by mTKI pretreatment (Fig. 6A). Western blot and qRT-PCR verified the upregulation of both β-catenin and ACVRL1 by Regorafenib, which is consistent with the RNA-seq results (Additional file 1: Figure S4A, B). On further exploration, we found that LiCl, a Wnt pathway activator, upregulated the expression of ACVRL1 in a time-dependent manner. In contrast, MSAB, a Wnt pathway inhibitor, significantly decreased the expression of ACVRL1 (Fig. 6B). We detected the expression of ACVRL1 and β-catenin in 40 pairs of human CRC tissues and found a significant positive correlation between the expression of ACVRL1 and β-catenin (Fig. 6C). By referring to the data in Transcription factor prediction databases (TFBIND), we found that the TCF/LEF family which are important effectors of β-catenin are not potential transcription factors of ACVRL1, which suggests β-catenin may regulate the expression of ACVRL1 at the post-transcriptional level. Three independent miRNA databases together predicted ten miRNAs that may target ACVRL1 (Fig. 6D) (Additional file 2: Table S3), of which five miRNAs (miR-149-5p, miR-7-5p, miR-3064-5p, miR-671-5p, and miR-3622b-5p) could be downregulated with the activation of Wnt/β-catenin pathway (Additional file 1: Figure S4C). Among the five miRNAs, only miR-7-5p was able to downregulate the expression of ACVRL1 (Fig. 6E). To further determine whether miR-7-5p inhibits ACVRL1 expression through a direct interaction, we performed dual luciferase reporter assays in CRC cells. Transfection with miR-7-5p mimics suppressed the luciferase activity of the ACVRL1 3′-UTR reporter constructs, whereas this effect was abolished when mutations were introduced into the seed sequences (Fig. 6F). Western blot and qRT-PCR analyses revealed that overexpression of miR-7-5p downregulated ACVRL1, whereas miR-7-5p inhibition upregulated ACVRL1 (Fig. 6G). The rescue experiments showed that the upregulation of ACVRL1 induced by LiCl was partially counteracted by the transfection of miR-7-5p mimics (Fig. 6H). Meanwhile, IHC and ISH staining indicated that ACVRL1 had no regulatory effect on miR-7-5p in tumour xenografts treated with Regorafenib (Additional file 1: Figure S4D). These results together suggest that the Wnt/β-catenin pathway upregulate ACVRL1 expression through the inhibition of miR-7-5p.

In order to further verify the molecular mechanism of the inhibition of miR-7-5p by Wnt/β-catenin pathway, we conducted the following explorations. Studies have shown that lncRNA-KCNQ1OT1 transcription is directly regulated by β-catenin in CRC [17]. To investigate whether β-catenin regulates lncRNA-KCNQ1OT1 transcription through association with the KCNQ1OT1 promoter, we selected a TCF-1 binding site that is predicted to be located closest to the transcription start site on KCNQ1OT1. We constructed human KCNQ1OT1 promoter-luciferase reporter plasmids: plasmid Kp2022 contained a 2020-bp fragment from within the KCNQ1OT1 promoter region that included this TCF-1 binding site, and plasmid Kp1080 contained a truncated fragment of the KCNQ1OT1 promoter that did not include this TCF-1 binding site (Fig. 6I). We then examined the effect of co-transfection of a β-catenin expressing vector or the control vector on the transcriptional activity of these KCNQ1OT1 promoters by measurement of luciferase activity. KCNQ1OT1 promoter activity in the β-catenin/Kp2022 transfected cells was significantly elevated when compared with β-catenin/Kp1080, whose reporter lacks the TCF-1 binding site (Fig. 6J). This result suggests that β-catenin regulates KCNQ1OT1 transcription through an effect on the KCNQ1OT1 promoter. To further determine whether β-catenin directly binds to a TCF site in the KCNQ1OT1 promoter, we performed ChIP analysis. In this assay, crosslinking of β-catenin to the TCF-1 binding site in the KCNQ1OT1 promoter in CRC cells was assayed. These results indicate that β-catenin directly binds to the TCF-1 site on the KCNQ1OT1 promoter (Fig. 6K).

Furthermore, the bioinformatic tool starbase v.2.0 [18] identified lncRNA-KCNQ1OT1 may be a candidate capable of regulating miR-7-5P. The miR-7-5p level was significantly elevated when KCNQ1OT1 was knocked down in CRC cells (Fig. 6L). Moreover, dual luciferase gene reporter assays showed that co-transfection of KCNQ1OT1-WT and miR-7-5P significantly decreased luciferase activities, while co-transfection of KCNQ1OT1-Mut and miR-7-5P had no effect on luciferase activities, when compared with the control group (Fig. 6M). The above results together suggest a novel Wnt/β-catenin/TCF-1-KCNQ1OT1-miR-7-5p axis mediating ACVRL1 activation, which may contribute to mTKI resistance in CRC.

### ACVRL1 inhibition sensitizes CRCs to mTKIs in vitro and in vivo

**Fig. 7:**
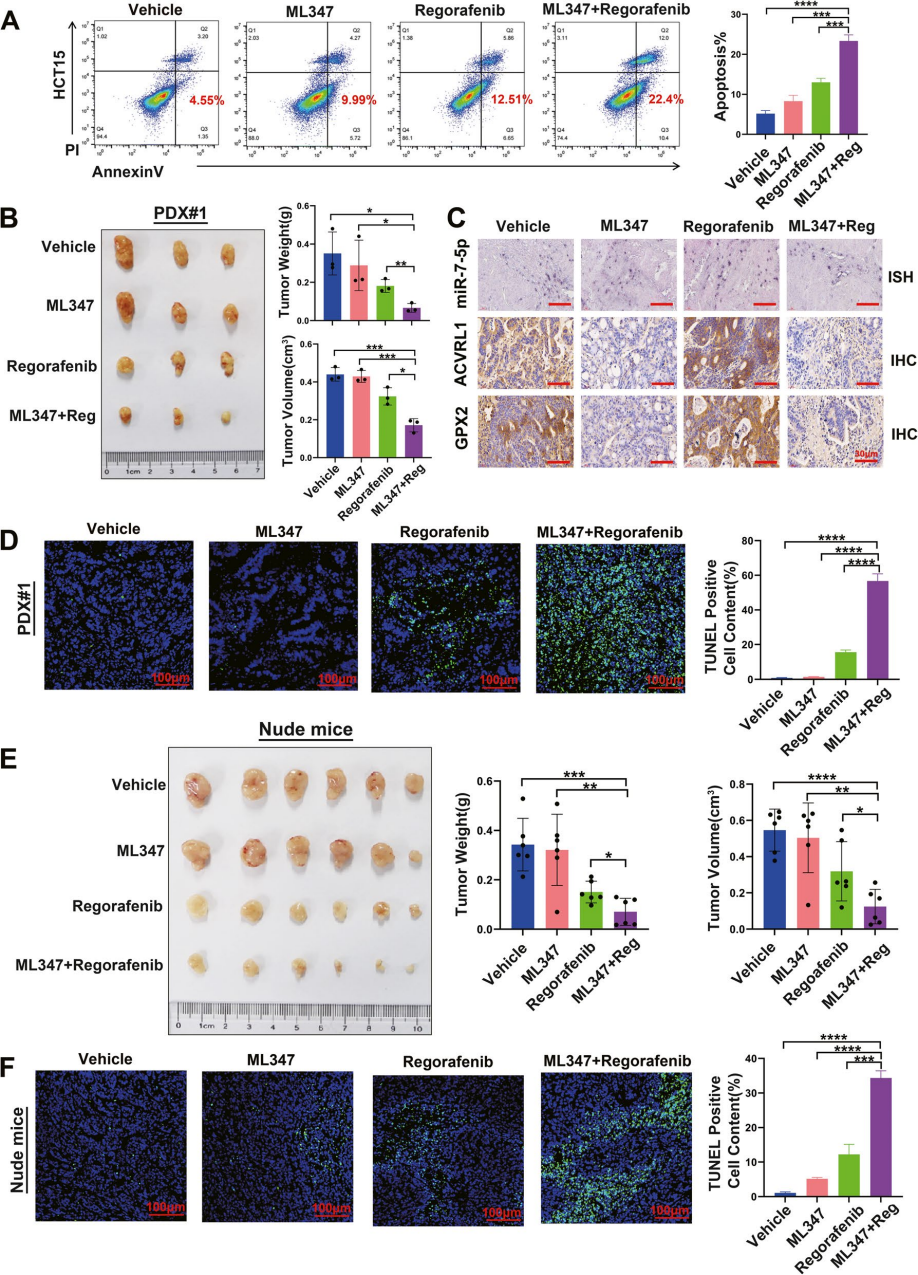
ACVRL1 inhibition sensitizes CRC to mTKI treatment in vitro and in vivo. **A** Annexin V-PI staining showed that Regorafenib can induce apoptosis of HCT15 cells, and Regorafenib combined with ML347 significantly increased the apoptosis ratio of HCT15 cells. **B, E** Regorafenib inhibited CRC tumourigenicity. ML347 and Regorafenib combination therapy effectively abolished CRC growth in PDX#1 model and nude mice model. **C** The representative tumour tissue sections from PDX#1 model in different drug treatment groups were analysed by IHC and ISH for the expressions of miR-7-5p, ACVRL1 and GPX2. Scale bar, 30 μm. **D, F** TUNEL staining of the transplanted tumour tissues of PDX#1 and nude mice models showed that Regorafenib combined with ML347 increased the ratio of tumour apoptosis (*P < 0.05, **P < 0.01, ***P < 0.001, ****P < 0.0001)

**Fig. 8:**
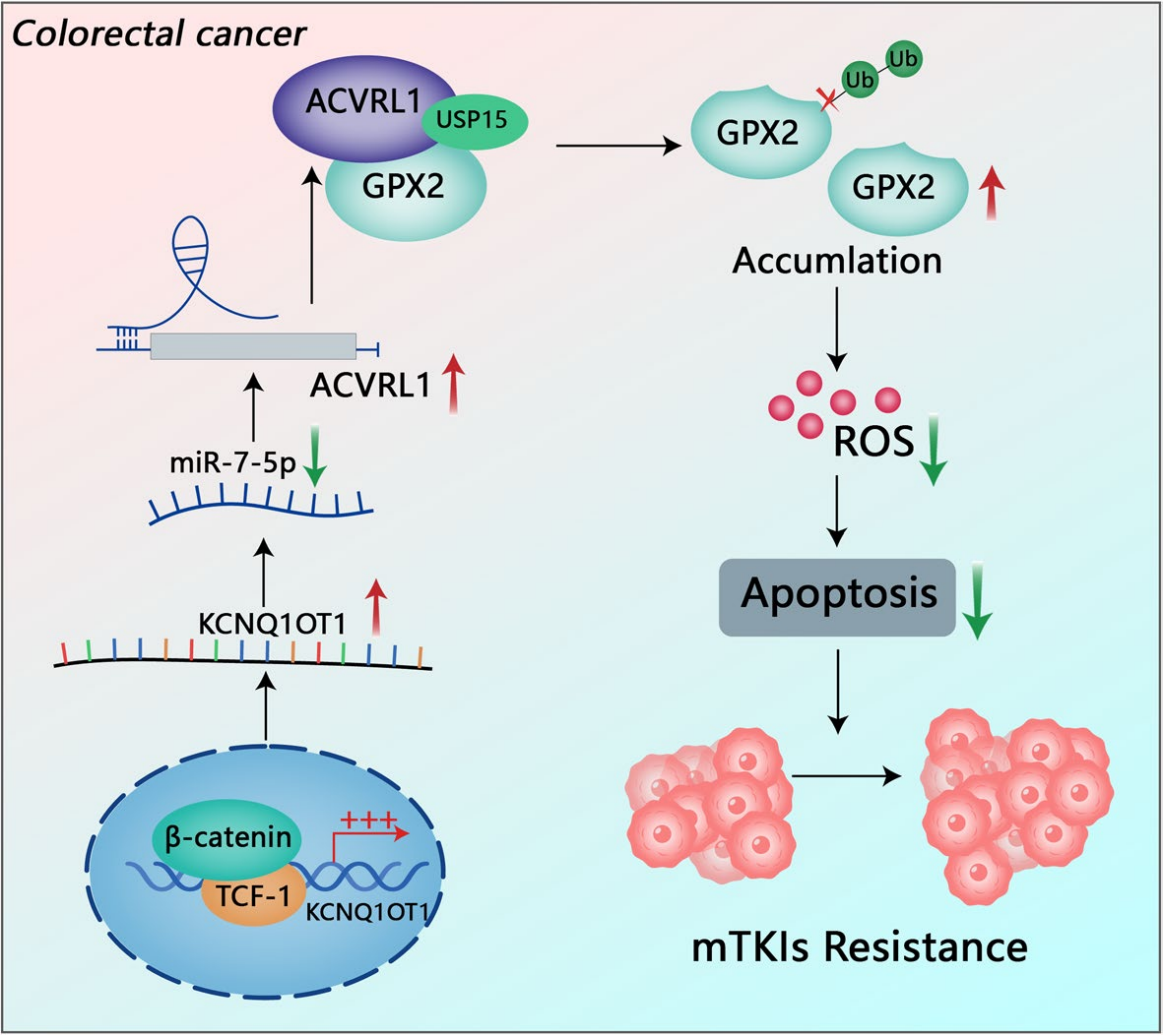
Graphical diagram. ACVRL1 is activated by the Wnt/β-catenin/KCNQ1OT1/miR-7-5p axis and interacts with USP15 to accumulate GPX2, which eventually leads to mTKI resistance

To further examine whether ACVRL1 inhibition could increase sensitivity to Regorafenib treatment in CRC, we treated HCT15 cells with vehicle (DMSO, concentration<1/1000), Regorafenib, ML347(a highly selective ACVRL1 inhibitor), and Regorafenib combined with ML347. The combination index (CI) value for different combination dosages of Regorafenib and ML347 is presented in Additional file 2: Table S4. The CI value for 10 µM Regorafenib and 25 µM ML347 was 0.88 in HCT15 cells. Thus, above combination dosage was used for the validation of further experiments. Annexin V and PI staining demonstrated that Regorafenib alone could induce CRC cells apoptosis after 48 h treatment. Strikingly, Regorafenib in combination with ML347 doubled the number of apoptotic cells, although ML347 itself only slightly induced apoptosis (Fig. 7A). Next, we used PDX models and HCT15 cell-derived xenografts to assess the combination of ML347 and Regorafenib in vivo. When tumours were palpable, the mice were randomly divided into four groups and treated respectively with vehicle, ML347, Regorafenib, Regorafenib and ML347 in combination. Regorafenib alone could reduce tumour growth, ML347 alone showed weak tumour inhibition ability. However, the combination of Regorafenib and ML347 completely inhibited tumour growth in vivo (Fig. 7B, E and Additional file 1: Figure S5A). Tumour cell apoptosis was further assessed by TUNEL assay in sections of PDX models and HCT15 cell-derived xenografts. There were significantly more apoptotic cells in the combined treatment group than in the ML347 or Regorafenib treatment groups (Fig. 7D, F and Additional file 1: Figure S5B). By ISH and IHC staining, we observed a same pattern for the expressions of ACVRL1 and GPX2 under different drug treatment, but for miR-7-5p, no obvious change was seen in different drug treatment groups (Fig. 7C). Taken together, these data confirm that ACVRL1 inhibition could effectively sensitize colon cancer cells to Regorafenib treatment in vivo Fig. 8.

## Discussion

Owing to the increasements of effective drugs and improvements in surgical procedures, the overall survival of mCRC after diagnosis is now approximately 30 months. Molecular targeted agents have played a vital role for improving the overall survival in mCRC [19]. mTKIs have achieved encouraging results in a variety of tumours, such as hepatocellular carcinoma, renal cell carcinoma, and gastrointestinal stromal tumour; however, their performance in CRC is unsatisfactory. The ORRs of Regorafenib were between 1 and 4% in the CORRECT and CONCUR trials [5, 20]. Further, the best response to Sorafenib in clinical trials for CRC was stable disease [21, 22]. The emergence of drug resistance is the main cause of mTKI treatment failure in CRC [23]. Previous studies have reported that FBW7 mutations can mediate Regorafenib resistance by blocking Mcl-1 degradation [24]. Activation of the Notch-1 signalling pathway is also involved in Regorafenib resistance [25]. As for Sorafenib, few studies have focused on the drug resistance mechanism of it in CRC [26]. The known molecular mechanism of mTKI resistance is very limited. Our study aims to explain the clinical problem of the low ORRs of mTKIs in CRC, which is urgently needed to be solved. RNA-seq identified ACVRL1 may be the driver gene for mTKI resistance. On further exploration, a novel β-catenin/ACVRL1/GPX2 axis activation was identified to be the underlined molecular mechanism which eventually leads to CRC cells resisting to mTKI treatment. ACVRL1 has the potential to be an efficiency predictor and applicable target for combined treatment to overcome mTKI resistance.

ACVRL1 was previously thought to be strictly expressed in endothelial cells [7]. Our study revealed the localisation and expression of ACVRL1 in the cytoplasm of CRC cells. Furthermore, our study revealed the biological function of ACVRL1 independent of the TGF-β pathway and angiogenesis. We found ACVRL1 promotes mTKI resistance independently by stabilizing GPX2, rather than through the classical TGF-β signalling pathway. ACVRL1 stabilizes GPX2 through USP15-mediated deubiquitination. The ROS-scavenging enzyme GPX2 is a critical determinant of tumour differentiation, growth, and metastasis in CRC. GPX2 maintains low intracellular ROS level, thereby maintaining the clonogenic and metastatic tumour cell populations in CRC [14]. Elevated GPX2 mediates the resistance of lung adenocarcinoma to cisplatin [27]. However, whether GPX2 is involved in drug resistance in CRC is still unclear. ROS are inevitable products of cellular metabolism in aerobic life and are closely related to drug resistance in tumours [28]. Our study reveals that upregulated ACVRL1 neutralizes ROS by stabilizing GPX2, leading to the intracellular ‘redox reset’, which contributes to the general resistance of CRC to mTKIs.

The Wnt/β-catenin signalling pathway is an evolutionarily conserved and unique signalling pathway that regulates cell proliferation, differentiation, invasion and metastasis during the initiation and progression of CRC [29]. Up to 80% of CRCs carries APC mutation, as a result, β-catenin accumulates, translocates to the nucleus, and associates with TCF/LEF, leading to subsequent transcriptional activation of genes associated with CRC development [30, 31]. Our study indicates that the low ORRs of mTKIs may partly due to the upregulated ACVRL1 which is activated by the general activation of the Wnt/β-catenin pathway in CRC. We further found that the TCF/LEF family are not potential transcription factors of ACVRL1 through the prediction of Transcription factor databases, so we hypothesized that β-catenin might regulate ACVRL1 at the post-transcriptional level. We next found that β-catenin could promote lncRNA-KCNQ1OT1 transcription through direct binding to the KCNQ1OT1 promoter. Dual luciferase reporter assay confirmed that miR-7-5p could directly bound to the 3’-UTR of KCNQ1OT1 and ACVRL1. The previous studies also have shown that KCNQ1OT may act as s miR-7-5p sponge regulating ferroptosis in neonatal rat ventricle cardiomyocytes [32]. In HCC, KCNQ1OT1 also has been reported to modulate oxaliplatin resistance through miR-7-5p/ABCC1 axis [33]. Taken together, KCNQ1OT1 may act as an endogenous sponge by binding miR-7-5p, thus abolishing the miRNA-induced repressing activity on the ACVRL1 3’-UTR. Finally, we uncovered that the β-catenin/TCF-1-KCNQ1OT1-miR-7-5p axis participates in the activation of ACVRL1, blocking which may be a potential strategy for reversing mTKI resistance in CRC.

Although we tried to make the design rigorous, there still have some limitations and deficiencies in our study. It was limited to models based on CRC cells and mouse xenografts. Though the PDX model was used, we did not perform a clinical cohort analysis based on patients treated with mTKIs, which made it impossible to objectively evaluate whether ACVRL1 could be used as a biomarker for mTKIs’ efficacy. We will continue to accumulate clinical evidence and try to confirm our conclusions based on real-world research as soon as possible.

## Conclusions

mTKIs are indispensable treatment options for patients with mCRC. Overcoming the resistance to mTKIs is critical for improving the overall survival and life quality of mCRC patients. Our study identified that ACVRL1 interacts with GPX2 mediating ROS clearance, which is the main mechanism leading to the resistance of CRC to mTKIs. Targeting the Wnt/β-catenin//KCNQ1OT1/miR-7-5p/ACVRL1/GPX2 biological axis has the potential to be an attractive method for addressing mTKI resistance in CRC.

### Supplementary Information

**Additional file 1:** **Figure S1.** Related to Fig. 1.**Figure S2.** Related to Fig. 2. **Figure S3.** Related to Fig. 4. **Figure S4.** Related to Fig. 6. **Figure S5.** Related to Fig. 7.** Figure S6.** Interference with the expression of ACVRL1 affects the survival and apoptosis ratio of CRC cells. **Figure S7.** Interference with the expression of ACVRL1 and GPX2 had no significant effect on the proliferation and differentiation of CRC cells under the treatment of Regorafenib.

**Additional file 2:** **Table S1.** The IC50 of Regorafenib. **Table S2.** The IC50 of Sorafenib. **Table S3.** Potential mircroRNAs targeting ACVRL1. **Table S4. **Combination index data for non-constant combinations. **Table S5.** Primers used for the qPCR reaction. **Table S6.** Reagents and antibodies used in this study. **Table S7.** Expression vectors used in this study. **Table S8. **SiRNA target sequences.

**Additional file 3.** Supplementary Materials and Methods.

**Additional file 4.** Data of RNA-seq.

**Additional file 5.** Data of LC-MS.

**Additional file 6.** Images of the original blot.

## Data Availability

The raw sequence data reported in this paper have been deposited in the Genome Sequence Archive (Genomics, Proteomics & Bioinformatics 2021) in National Genomics Data Center (Nucleic Acids Res 2021), China National Center for Bioinformation / Beijing Institute of Genomics, Chinese Academy of Sciences (GSA: HRA004669) that are publicly accessible at https://ngdc.cncb.ac.cn/gsa.

## Abbreviations

ACVRL1: Activin A receptor-like type 1
CHIP: Chromatin immunoprecipitation assay
CRC: Colorectal cancer
GPX2: Glutathione peroxidase 2
IP: Immunoprecipitation
LC-MS: Liquid chromatography-mass spectrometry
mTKIs: Multitarget tyrosine kinase inhibitors
USP15: Ubiquitin-specific peptidase 15

## References

[1] Lichtenstern CR, Ngu RK, Shalapour S, Michael K (2020). Immunotherapy, inflammation and colorectal cancer. Cells.

[2] Bray F, Ferlay J, Soerjomataram I, Siegel RL, Torre LA, Jemal A (2018). Global cancer statistics 2018: GLOBOCAN estimates of incidence and mortality worldwide for 36 cancers in 185 countries. CA Cancer J Clin.

[3] Biller LH, Schrag D (2021). Diagnosis and treatment of metastatic colorectal cancer: a review. JAMA.

[4] Huang L, Jiang S, Shi Y (2020). Tyrosine kinase inhibitors for solid tumors in the past 20 years (2001–2020). J Hematol Oncol.

[5] Grothey A, Van Cutsem E, Sobrero A, Siena S, Falcone A, Ychou M (2013). Regorafenib monotherapy for previously treated metastatic colorectal cancer (CORRECT): an international, multicentre, randomised, placebo-controlled, phase 3 trial. Lancet.

[6] Strumberg D, Clark JW, Awada A, Moore MJ, Richly H, Hendlisz A (2007). Safety, pharmacokinetics, and preliminary antitumor activity of Sorafenib: a review of four phase I trials in patients with advanced refractory solid tumors. Oncologist.

[7] Bocci M, Sjölund J, Kurzejamska E, Lindgren D, Marzouka NA, Bartoschek M (2019). Activin receptor-like kinase 1 is associated with immune cell infiltration and regulates CLEC14A transcription in cancer. Angiogenesis.

[8] Cunha SI, Pardali E, Thorikay M, Anderberg C, Hawinkels L, Goumans MJ (2010). Genetic and pharmacological targeting of activin receptor-like kinase 1 impairs tumor growth and angiogenesis. J Exp Med.

[9] Hu-Lowe DD, Chen E, Zhang L, Watson KD, Mancuso P, Lappin P (2011). Targeting activin receptor-like kinase 1 inhibits angiogenesis and tumorigenesis through a mechanism of action complementary to anti-VEGF therapies. Cancer Res.

[10] Cunha SI, Bocci M, Lövrot J, Eleftheriou N, Roswall P, Cordero E (2015). Endothelial ALK1 is a therapeutic target to block metastatic dissemination of breast cancer. Cancer Res.

[11] Liu C, Shi J, Li Q, Li Z, Lou C, Zhao Q (2019). STAT1-mediated inhibition of FOXM1 enhances gemcitabine sensitivity in pancreatic cancer. Clin Sci (Lond).

[12] The Human Protein Atlas. https://www.proteinatlas.org/ENSG00000- 139567. Accessed 10 Feb 2021.

[13] TCGA Research Network. https://www.cancer.gov/tcga. Accessed 10 Feb 2021.

[14] Emmink BL, Laoukili J, Kipp AP, Koster J, Govaert KM, Fatrai S (2014). GPx2 suppression of H2O2 stress links the formation of differentiated tumor mass to metastatic capacity in colorectal cancer. Cancer Res.

[15] Zhang L, Zhou F, Drabsch Y, Gao R, Snaar-Jagalska BE, Mickanin C (2012). USP4 is regulated by Akt phosphoyla- tion and deubiquitylates TGF-beta type I receptor. Nat Cell Biol.

[16] BDM-PUB. http://bdmpub.biocuckoo.org/prediction.php. Accessed 6 May 2021.

[17] Sunamura N, Ohira T, Kataoka M, Inaoka D, Kugoh H (2016). Regulation of functional KCNQ1OT1 lncRNA by β-catenin. Sci Rep.

[18] Starbase v.2.0. http://starbase.sysu.edu.cn/. Accessed 26 Oct 2022.

[19] De Falco V, Napolitano S, Roselló S, Huerta M, Cervantes A, Ciardiello F (2020). How we treat metastatic colorectal cancer. ESMO Open.

[20] Li J, Qin S, Xu R, Yau TC, Ma B, Pan H (2015). Regorafenib plus best supportive care versus placebo plus best supportive care in Asian patients with previously treated metastatic colorectal cancer (CONCUR): a randomised, double-blind, placebo-controlled, phase 3 trial. Lancet Oncol.

[21] Strumberg D, Richly H, Hilger RA, Schleucher N, Korfee S, Tewes M (2005). Phase I clinical and pharmacokinetic study of the novel Raf kinase and vascular endothelial growth factor receptor inhibitor BAY 43–9006 in patients with advanced refractory solid tumors. J Clin Oncol.

[22] Awada A, Hendlisz A, Gil T, Bartholomeus S, Mano M, de Valeriola D (2005). Phase I safety and pharmacokinetics of BAY 43–9006 administered for 21 days on/7 days off in patients with advanced, refractory solid tumours. Br J Cancer.

[23] Mirone G, Shukla A, Marfe G (2016). Signaling mechanisms of resistance to EGFR- and anti-angiogenic inhibitors cancer. Crit Rev Oncol Hematol.

[24] Tong J, Tan S, Zou F, Yu J, Zhang L (2017). FBW7 mutations mediate resistance of colorectal cancer to targeted therapies by blocking Mcl-1 degradation. Oncogene.

[25] Mirone G, Perna S, Shukla A, Marfe G (2016). Involvement of Notch-1 in resistance to Regorafenib in colon cancer cells. J Cell Physiol.

[26] Tang W, Chen Z, Zhang W, Cheng Y, Zhang B, Wu F (2020). The mechanisms of Sorafenib resistance in hepatocellular carcinoma: theoretical basis and therapeutic aspects. Signal Transduct Target Ther.

[27] Du H, Chen B, Jiao NL, Liu YH, Sun SY, Zhang YW (2020). Elevated glutathione peroxidase 2 expression promotes cisplatin resistance in lung adenocarcinoma. Oxid Med Cell Longev.

[28] Cui Q, Wang JQ, Assaraf YG, Ren L, Gupta P, Wei L (2018). Modulating ROS to overcome multidrug resistance in cancer. Drug Resist Updat.

[29] Muzny DM, Bainbridge MN, Chang K, Dinh HH, Drummond JA, Fowler G (2012). Comprehensive molecular characterization of human colon and rectal cancer. Nature.

[30] Morin PJ, Sparks AB, Korinek V, Barker N, Clevers H, Vogelstein B (1997). Activation of beta-catenin-Tcf signaling in colon cancer by mutations in beta-catenin or APC. Science.

[31] Markowitz SD, Bertagnolli MM (2009). Molecular origins of cancer: molecular basis of colorectal cancer. N Engl J Med.

[32] Zhuang Shaowei, Ma Yan, Zeng Yuxiao, Cheng Lu, Yang Fenghua, Jiang Nianxin (2021). METTL14 promotes doxorubicin-induced cardiomyocyte ferroptosis by regulating the KCNQ1OT1-miR-7-5p-TFRC axis. Cell Biol Toxicol.

[33] Hu H, Yang L, Li L, Zeng C (2018). Long non-coding RNA KCNQ1OT1 modulates oxaliplatin resistance in hepatocellular carcinoma through miR-7-5p/ABCC1 axis. Biochem Biophys Res Commun.

