# Supplementary material for: ACVRL1 drives resistance to multitarget tyrosine kinase inhibitors in colorectal cancer by promoting USP15-mediated GPX2 stabilization
Source: BMC Med. 2023 Sep 25;21:366. doi: 10.1186/s12916-023-03066-4 (PMC10518977; doi:10.1186/s12916-023-03066-4)
Supplement: Supplementary file 2 — Additional file 2: Table S1. The IC50 of Regorafenib. Table S2. The IC50 of Sorafenib. Table S3. Potential mircroRNAs targeting ACVRL1. Table S4. Combination index data for non-constant combinations. Table S5. Primers used for the qPCR reaction. Table S6. Reagents and antibodies used in this study. Table S7. Expression vectors used in this study. Table S8. SiRNA target sequences. [file 12916_2023_3066_MOESM2_ESM.docx]

**Table S1** The IC50 of Regorafenib

| REG/cell | LS174T | HCT15 | SW1116 | DLD1 | RKO | HT29 | LOVO | HCT116 | SW48 | SW620 | SW480 | SW948 |
| --- | --- | --- | --- | --- | --- | --- | --- | --- | --- | --- | --- | --- |
| IC50(mmol) | 22.4 | 17.6 | 17.4 | 16.8 | 11.2 | 7.5 | 7.3 | 6.8 | 6.6 | 5.4 | 5.0 | 3.9 |

**Table S2** The IC50 of Sorafenib

| REG/cell | LS174T | SW1116 | HCT15 | DLD1 | HT29 | HCT116 | RKO | SW620 | LOVO | SW948 | SW480 | SW48 |
| --- | --- | --- | --- | --- | --- | --- | --- | --- | --- | --- | --- | --- |
| IC50(mmol) | 11.7 | 11.4 | 9.1 | 8.1 | 6.8 | 5.5 | 5.1 | 4.8 | 2.4 | 2.3 | 1.7 | 0.4 |

**Table S3** Potential mircroRNAs targeting ACVRL1

| miR-7-5p | miR-3064-5p | miR-532-3p | miR-485-5p |
| --- | --- | --- | --- |
| miR-149-5p | miR-3622b-5p | miR-214-3p | miR-3619-5p |
| miR-671-5p | miR-670-5p |  |  |

| ML347(µM) | Regorafenib(µM) | Effect (FA) | Combination Index (CI） |
| --- | --- | --- | --- |
| 5 | 10 | 0.29 | 1.30 |
| 10 | 10 | 0.48 | 0.93 |
| 25 | 10 | 0.62 | 0.88 |
| 50 | 10 | 0.79 | 0.71 |

**Table S4**  Combination index data for non-constant combinations

| Target | Species | Primers | Sequences (5’→3’) |
| --- | --- | --- | --- |
| ACVRL1 | Human | Forward | CATCGCTCAGACATGACCTC |
|  |  | Reverse | GTTTGCCCTGTGTACCGAAGA |
| GPX2 | Human | Forward | GGTAGATTTCAATACGTTCCGGG |
|  |  | Reverse | TGACAGTTCTCCTGATGTCCAAA |
| KCNQ1OT1 | Human | Forward | CAGGCACAGACGTTCTGAAG |
|  |  | Reverse | CACAGCCTCTCGTTGTTCTG |
| miR-7-5P | Human | Reverse transcript primers | GTCGTATCCAGTGCAGGGTCCGAGGTA TTCGCACTGGATACGACAACAAC |
|  |  | Forward | CGTggaagacTagTgaTTTTgTTgTT |
|  |  | Reverse | ATCCAGTGCAGGGTCCGAGG |
| TCF-1 site-specific detection primers of KCNQ1OT1 | Human | Forward | TTGAACACGGTCAGCACG |
|  |  | Reverse | CAGCCCACTCTGAAC CACC |

**Table S5** Primers used for the qPCR reaction

**Table S6** Reagents and antibodies used in this study

| Reagents and antibodies | Source | Identifier |
| --- | --- | --- |
| Regorafenib | MedChemExpress | HY-10331 |
| Sorafenib | MedChemExpress | HY-10201 |
| Cycloheximide (CHX) | MedChemExpress | HY-12320 |
| MG132 | MedChemExpress | HY-13259 |
| ML347 | MedChemExpress | HY-12274 |
| Anti-ACVRL1 | Abcam | ab68703 |
| Anti-ACVRL1 | Santa Cruz | sc-101556 |
| Anti-GPX2 | Abcam | ab137431 |
| Anti-GPX2 | Santa Cruz | sc-133160 |
| Anti-USP15 | Abcam | ab71713 |
| Anti-HA-tag | Abcam | ab236632 |
| Anti-Myc tag | Abcam | ab32 |
| Anti-Flag | Abcam | ab205606 |
| Anti-β-actin | ABclonal Technology | AC026 |
| Anti-β-catenin | Wanleibio | WL0962a |
| Anti-β-catenin | Abcam | ab32572 |
| Anti-Bcl-2 | Abcam | ab182858 |
| Anti-BAX | Abcam | ab32503 |
| Anti-Cleaved PARP | Abcam | ab32064 |
| Anti-MUC2 | Abcam | ab272692 |
| Anti-Cytokeratin 20 | Proteintech | 17329-1-AP |
| Anti-Ki67 | Abcam | ab16667 |

**Table S7** Expression vectors used in this study

| Recombinant DNA | Source |
| --- | --- |
| pENTER | This study |
| pLent-U6 | This study |
| pENTER-ACVRL1-Flag | WZ Biosciences Inc |
| pENTER-ACVRL1-1-141-Flag | GENERAL BIOL |
| pENTER-ACVRL1-142-281-Flag | GENERAL BIOL |
| pENTER-ACVRL1-282-503-Flag | GENERAL BIOL |
| pENTER-GPX2-Flag | WZ Biosciences Inc |
| pCMV-GPX2-HA | GENERAL BIOL |
| pCMV-GPX2(K187R)-HA | GENERAL BIOL |
| pCMV-GPX2(K120R)-HA | GENERAL BIOL |
| pCMV-GPX2(K87R)-HA | GENERAL BIOL |
| pCMV-GPX2(K20R)-HA | GENERAL BIOL |
| pCMV-GPX2(K6R)-HA | GENERAL BIOL |
| pENTER-USP15-Flag | WZ Biosciences Inc |
| pRK5-Ubiquitin-HA | This study |
| pCDNA3.1-Ubiquitin-Myc | WZ Biosciences Inc |

| Target | Species | Sequences (5′→3′) |
| --- | --- | --- |
| shACVRL1#1 | Human | GCGGATCAAGAAGACACTACATTCAAGAGATGTATGTCTTCTTGATCCGCTTTTTT |
| shACVRL1#2 | Human | GCTGGGAGAGTCCAGTCTCATTTCAAGAGAATGAGACTGGACTCTCCCAGCTTTTTT |
| shGPX2 | Human | CCTACCCTTATGATGACCCATTTCAAGAGAATGGGTCATCATAAGGGTAGGTTTTTT |
| miR-7-5P mimic | Human | UGGAAGACUAGUGAUUUUGUUGUUAACAACAAAAUCACUAGUCUUCCA |
| KCNQ1OT1 siRNA#1 | Human | GCACAAACCUCAAGACUAATTUUAGUCUUGAGGUUUGUGCTT |
| KCNQ1OT1 siRNA#2 | Human | GCUAAGAACCACAGGAUAATTUUAUCCUGUGGUUCUUAGCTT |
| shUSP15 | Human | GATACAGAGCACGTGATTATTTCAAGAGAATAATCACGTGCTCTGTATCTTTTTT |

**Table S8** SiRNA target sequences
