# Supplementary material for: ACVRL1 drives resistance to multitarget tyrosine kinase inhibitors in colorectal cancer by promoting USP15-mediated GPX2 stabilization
Source: BMC Med. 2023 Sep 25;21:366. doi: 10.1186/s12916-023-03066-4 (PMC10518977; doi:10.1186/s12916-023-03066-4)
Supplement: Supplementary file 3 — Additional file 3. Supplementary Materials and Methods. [file 12916_2023_3066_MOESM3_ESM.doc]

**Supplementary Materials and Methods**

## **Western blot and qRT-PCR**

## The protein extracts were prepared according to standard protocols. Cell lysates were separated by 10% SDS-PAGE and transferred to PVDF membranes(Millipore, Germany). The membranes were incubated with specific primary antibodys at 4℃ overnight. The protein immunoreactive signals were tested by the ECL detection system (Thermo Fisher,USA). The results were quantified using the image analysis tool ImageLab. Each experiment was repeated three times. The mRNA expression was determined by qRT-PCR. Total RNA was extracted using TRIZOL according to standard protocols. The cDNA was synthesized from 1 μg of total RNA by PrimeScript RT Master Mix (TakaraBio,Japan ). The sequences of primers were as Supplementary Table S5.

**CCK-8 and Colony formation assay**

The cells were inoculated into 96-well plates at a cell density of 4×10^3^ cells per well and incubated at the indicated time point after TKIs treatment. Then, the cells were incubated with 10 µL CCK-8 for 60 min at 37 °C, 5% CO2 condition. The absorption value was conducted in triplicate and repeated three times. Absorbance at 450 nm was measured using a microplate reader. The transfected cells were plated in 6-well plates at a density of 1000 cells/well with indicated drug concentration, the medium is changed daily. After 10 days of culture, the cells were washed with PBS, fixed with 4% paraformaldehyde, and stained with crystal purple for 20 minutes. The stained colonies were then photographed. Clone formation counts were performed using the imageJ software.

**Apoptosis assay**

## [Transfect](../../../../C:/Program%2520Files%2520(x86)/Youdao/Dict/8.9.6.0/resultui/html/index.html#/javascript:;)ed cells were seeded at 2 × 10^5^ cells/well in a six-well plate in triplicate. After 48 h of treatment with TKIs, cells were harvested to assess apoptosis. Apoptosis was assessed using the Annexin V-FITC Apoptosis Analysis Kit (AO2001-02P-H, Tianjin Sungene Biotech Co) according to the manufacturer’s instructions. Stained cells were analysed using a FACSCanto II Analyzer (BD Biosciences, USA). Data were analysed using FlowJo software (USA).

## **Immunofluorescence(IF)**

Cells were plated on coverslips, fixed with 4% paraformaldehyde for 20 min, and permeabilised in PBS containing 0.3% Triton X-100 for 10 min. Cells were then incubated in blocking buffer (5% BSA in PBS) at room temperature for 1 h, followed by staining with primary antibodies. The coverslips were washed and incubated for 1 h with the secondary antibodies and then stained with DAPI and blocked with glycerol. Fluorescence and confocal microscopy (Olympus, Japan) was used to detect the fluorescence of cells.

**Immunohistochemistry(IHC) and in situ hybridization(ISH)**

Tissue microarray（TMA）was purchased from SHANGHAI OUTDO BIOTECH CO.,LTD(China). Paraffin-embedded tumor tissues were cut into 4 μm sections. These sections were de-paraffinised, heated in citrate buffer (0.01 M), treated with 0.3% H_2_O_2_, and rehydrated. The sections were then incubated with anti-ACVRL1 antibody (1:500 dilution, ab68703, Abcam) or anti-GPX2 antibody (1:500 dilution, ab137431, Abcam) at 4°C overnight. The next day, the sections were incubated with biotinylated secondary antibody, exposed to diaminobenzidine, and counterstained with haematoxylin. For ISH, a 5’- and 3’- digoxigenin (DIG)-labeled locked nucleic acid-based probe specific for miR-7-5p (BOSTER) was incubated with the same tissue microarray chip. The results of IHC and ISH were independently scored by two independent observers. Expression levels were visualized and classified based on the percentage of positive cells and the intensity of staining.

**TUNEL assay**

The One Step TUNEL Apoptosis Assay Kit (C1088, Beyotime) was used to assess the apoptosis in the tumor samples. The percentage of positive cells was determined by scanning the slides with an Aperio CS Scanscope scanner (Aperio, Vista, CA, USA) and quantified by imageJ software (USA).

**Reactive oxygen species measurement**To determine the ROS concentration, CRC cells were seeded into six-well plates. The ROS assay kit (S0033S, [Beyotime Biotechnology](https://www.so.com/link?m=bLfVgv2mX+1l2UdoMi5aUDwnElVLtKncNJdwqJHdLhw2j3DY4l/Lx6YEsR2e8AmaQ0R+nQhVuFKNBnU4ARwN37cgKDGN8SpW4LHxw+JGaQytQmi9r4opJO5XOgSqrH+3ufzm47g==), China) was used to detect intracellular ROS levels according to the manufacturer’s instructions, and the harvested cells were analysed using the FACSCanto II Analyzer (BD Biosciences, USA). The results were analysed using FlowJo software(USA).
