# Supplementary material for: ACVRL1 drives resistance to multitarget tyrosine kinase inhibitors in colorectal cancer by promoting USP15-mediated GPX2 stabilization
Source: BMC Med. 2023 Sep 25;21:366. doi: 10.1186/s12916-023-03066-4 (PMC10518977; doi:10.1186/s12916-023-03066-4)
Supplement: Supplementary file 6 — Additional file 6. Images of the original blot. [file 12916_2023_3066_MOESM6_ESM.doc]

Fig1.E

ACVRL1


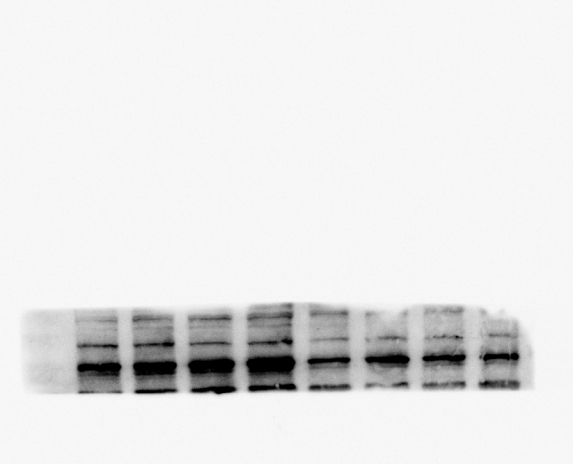


ACTIN


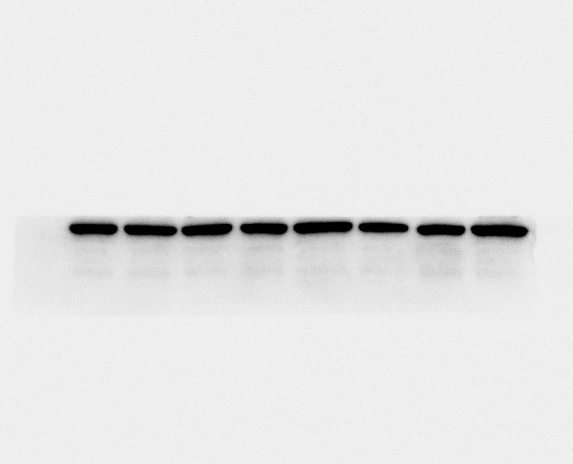


Fig1.H

ACVRL1


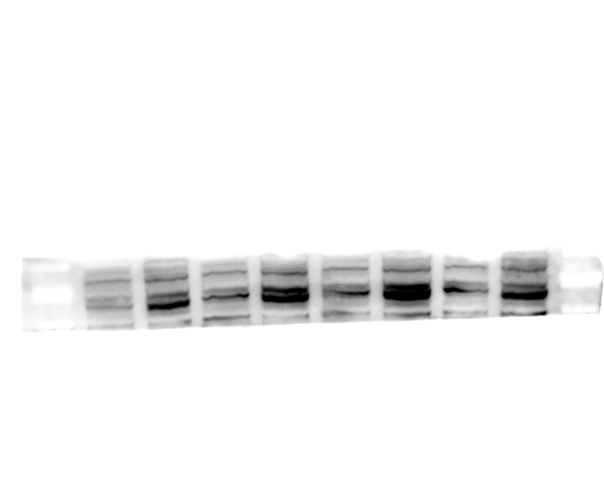


ACTIN


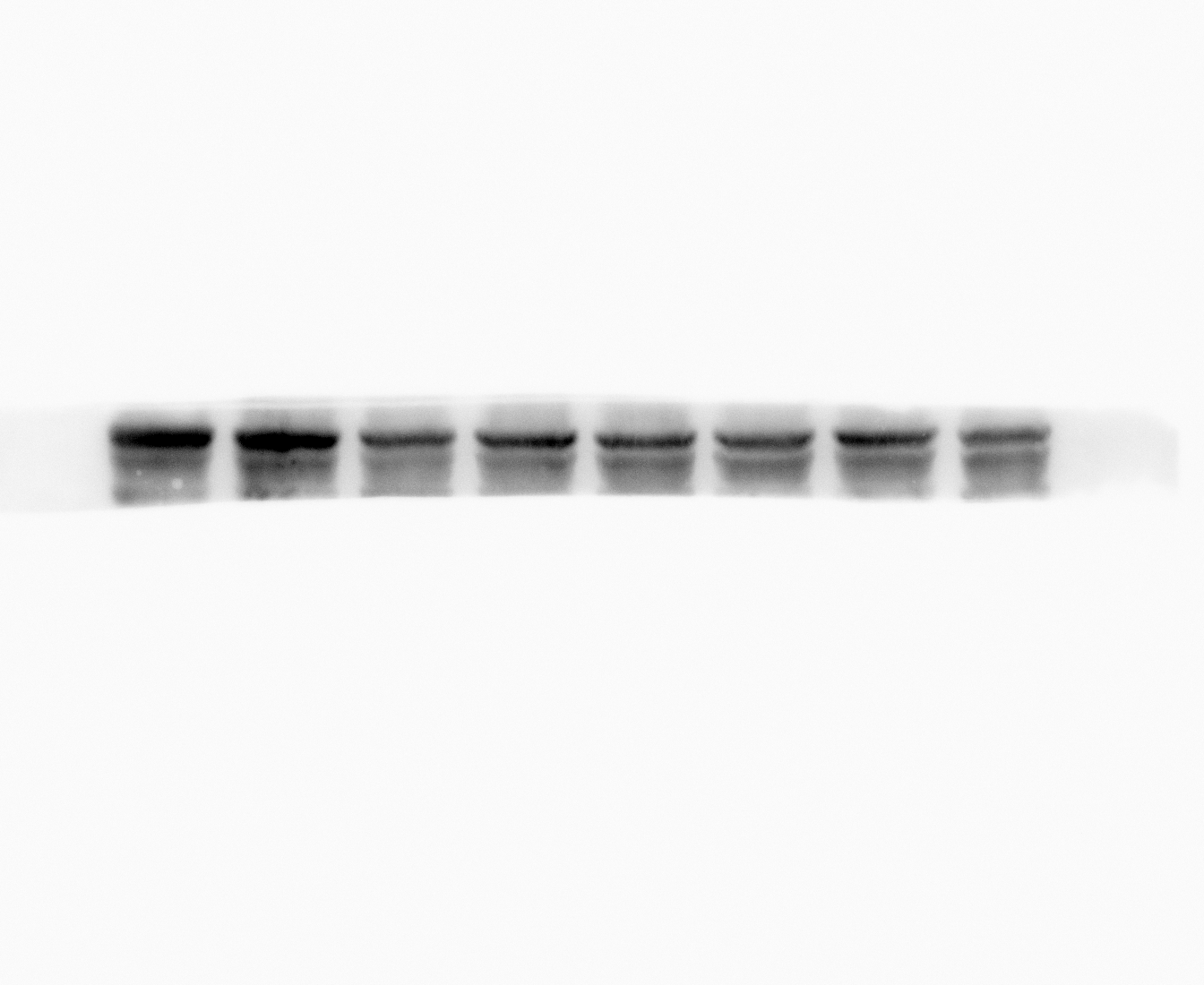


Fig2.A

HCT15-ACVRL1(Lane 1-3)


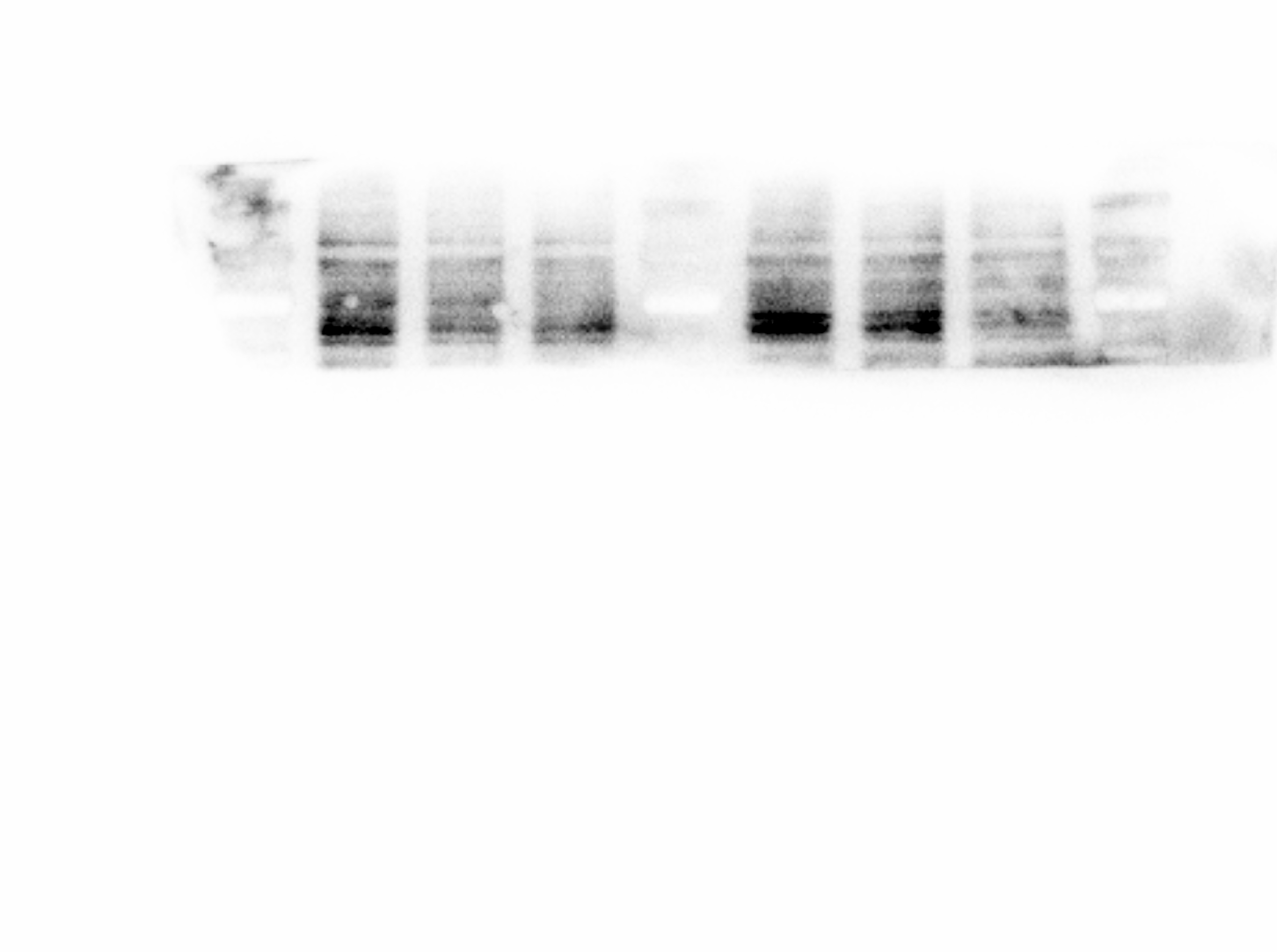


HCT15-ACTIN(Lane 1-3)


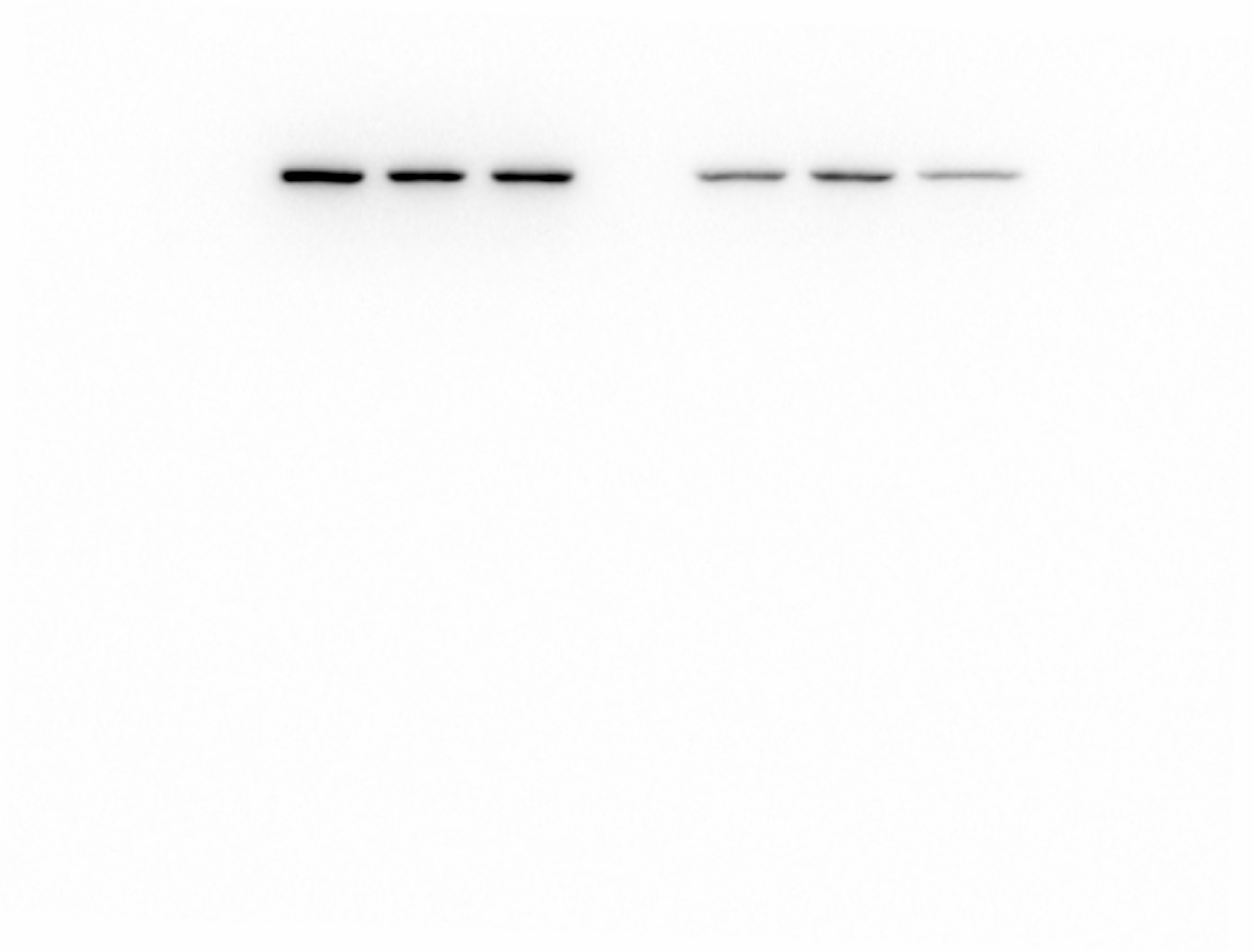


LS174T-ACVRL1(Lane 1-3)


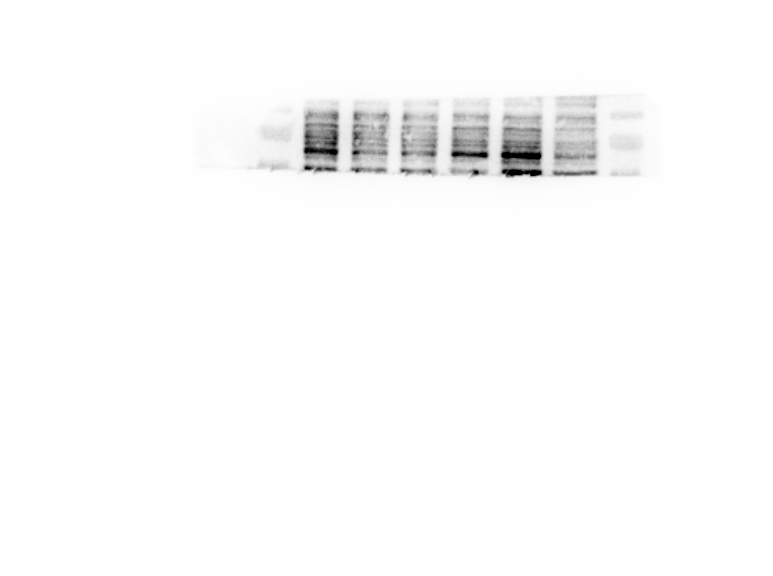


LS174T-ACTIN(Lane 1-3)


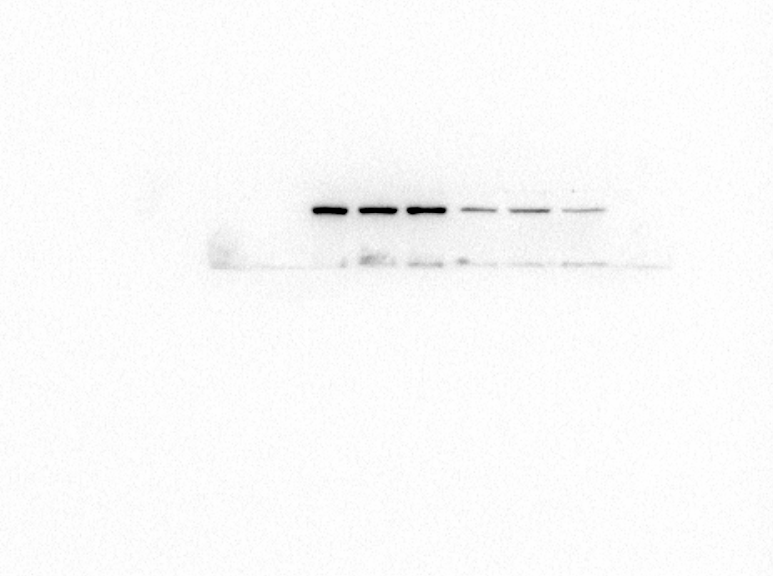


HCT116-ACVRL1(Lane 1-2)


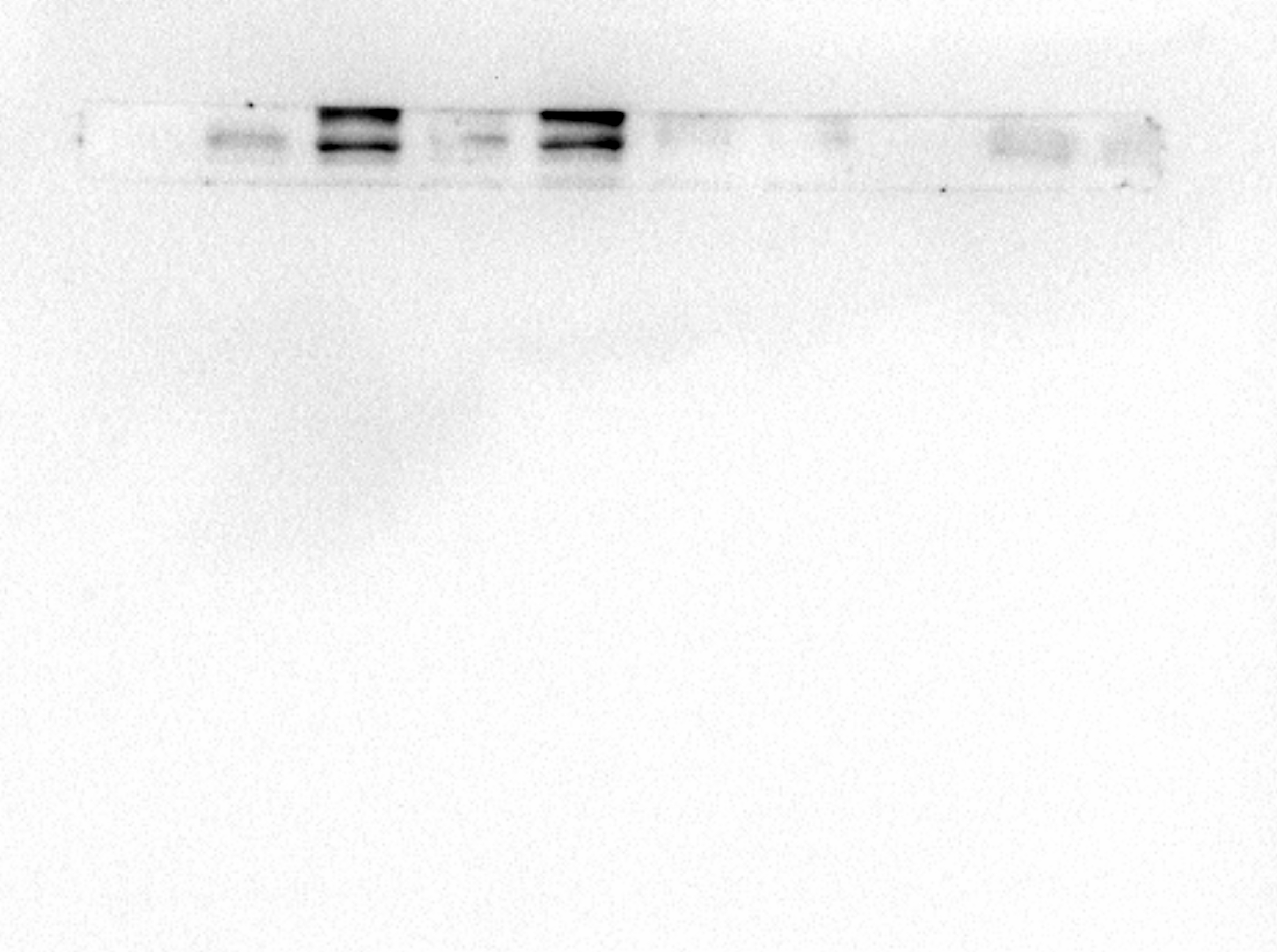


HCT116-ACTIN(Lane 1-2)


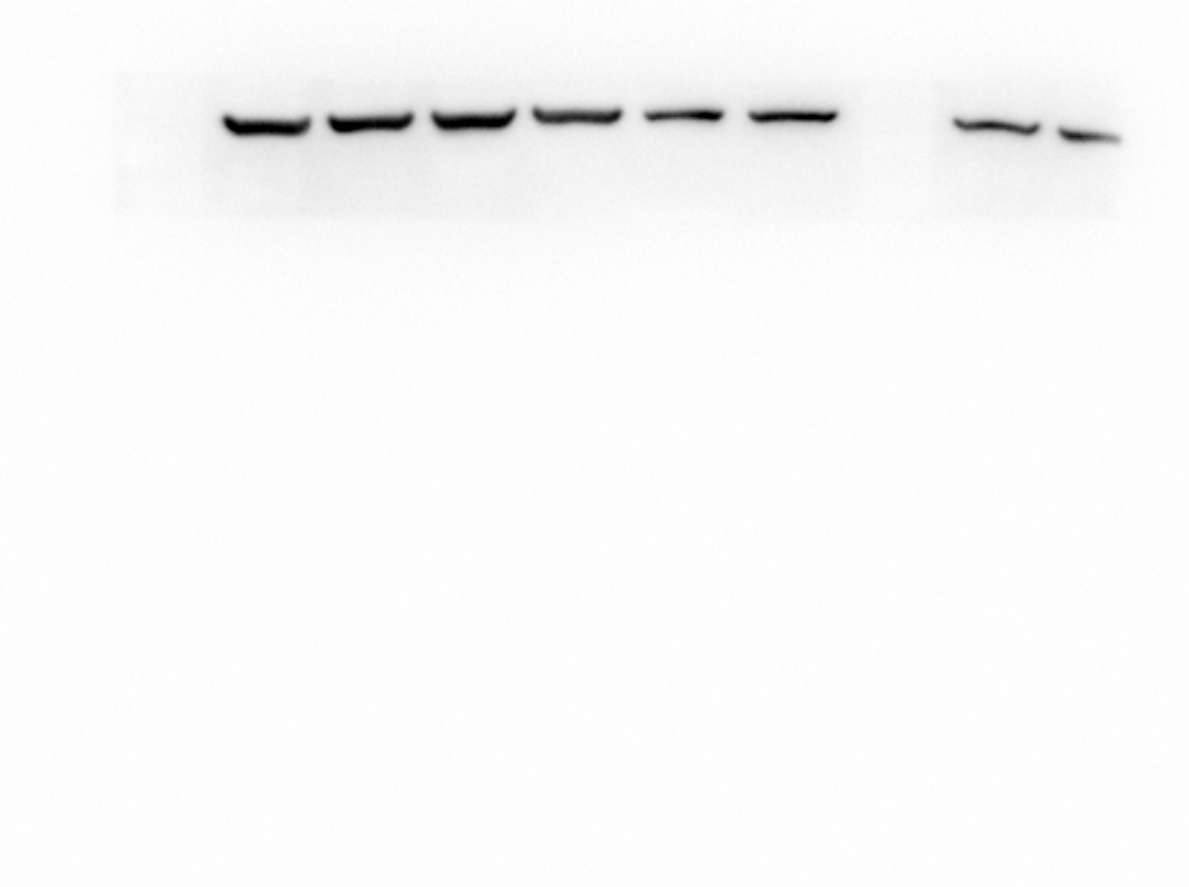


Fig3.A


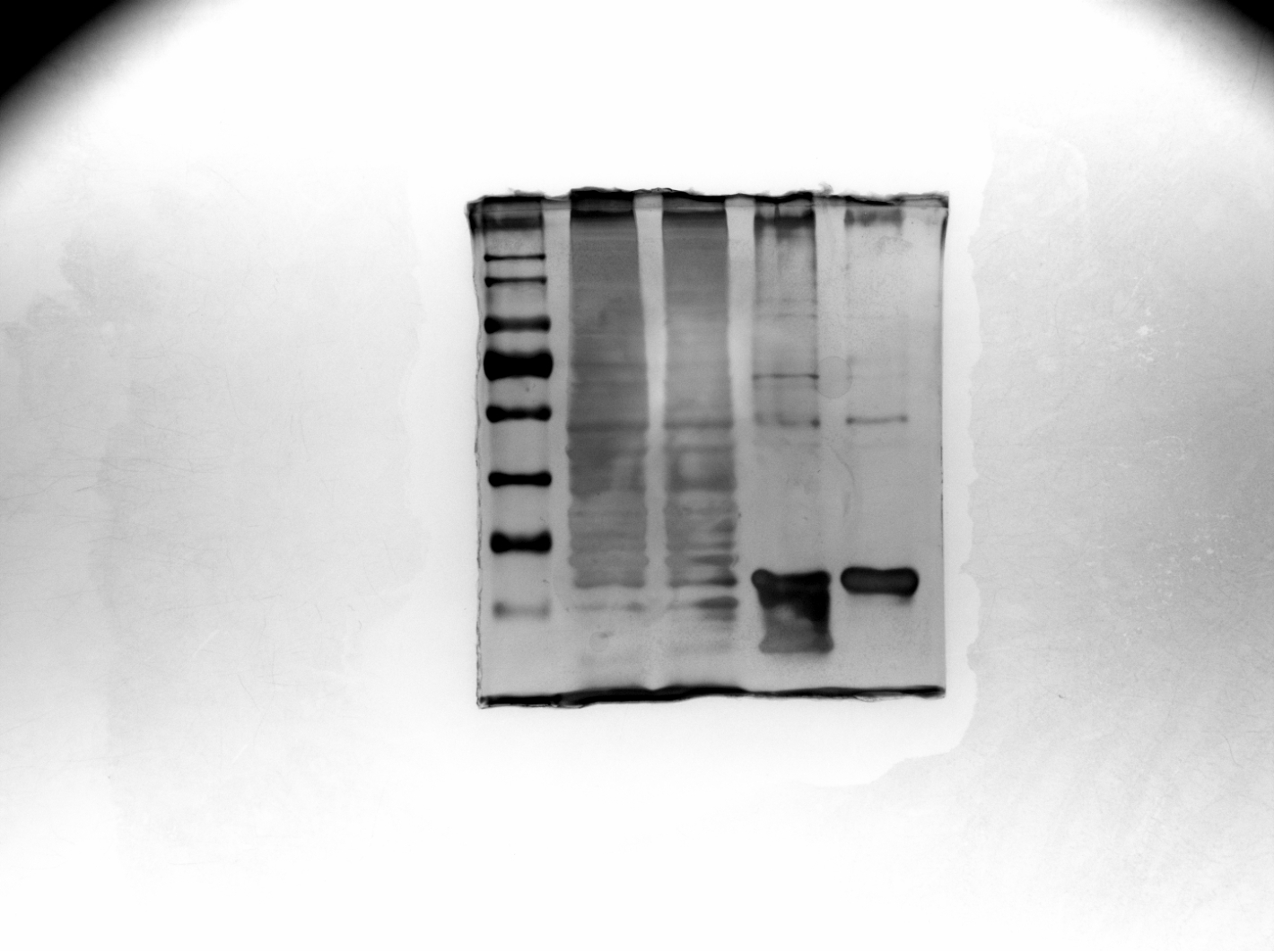


Fig. 3B

Input-ACVRL1(Lane1-2） IP-Flag（Lane3-4）


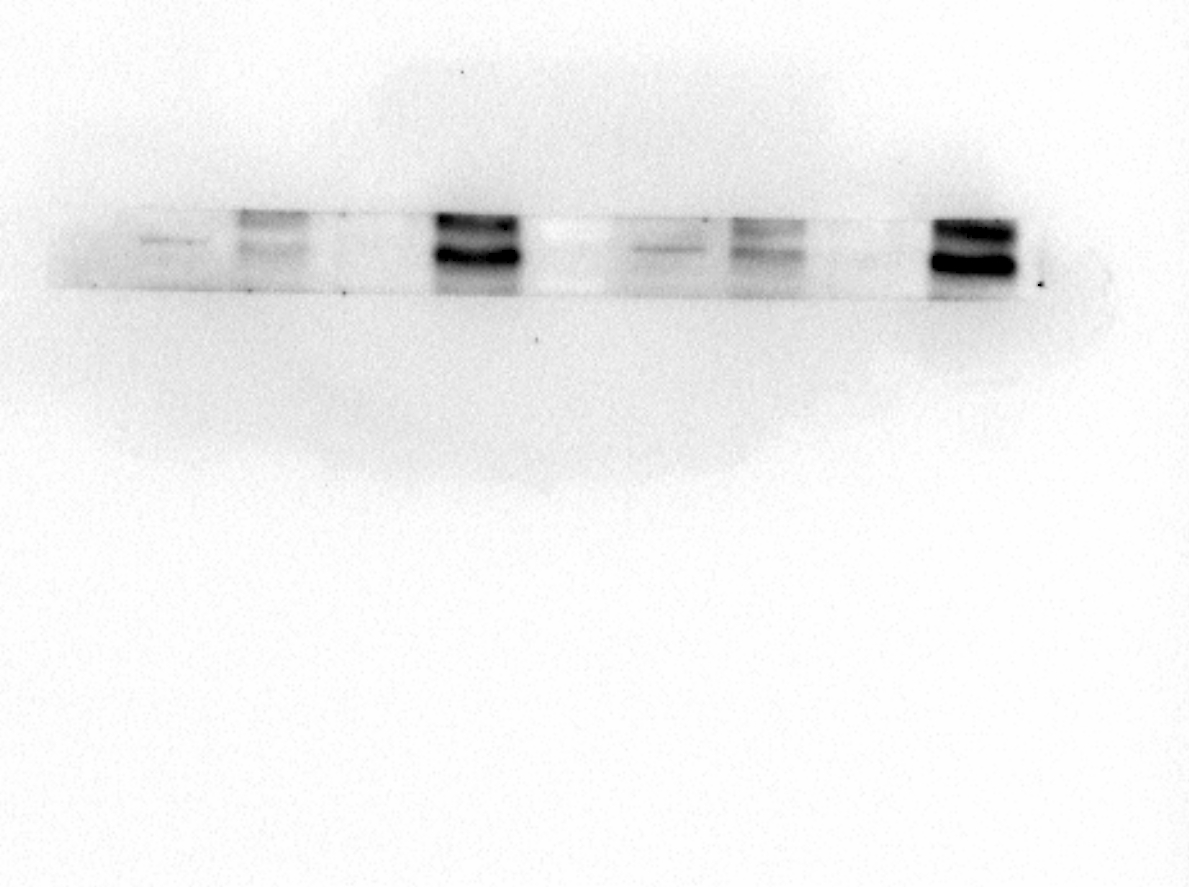


Input-GPX2(Lane1-2） IP-GPX2（Lane3-4）


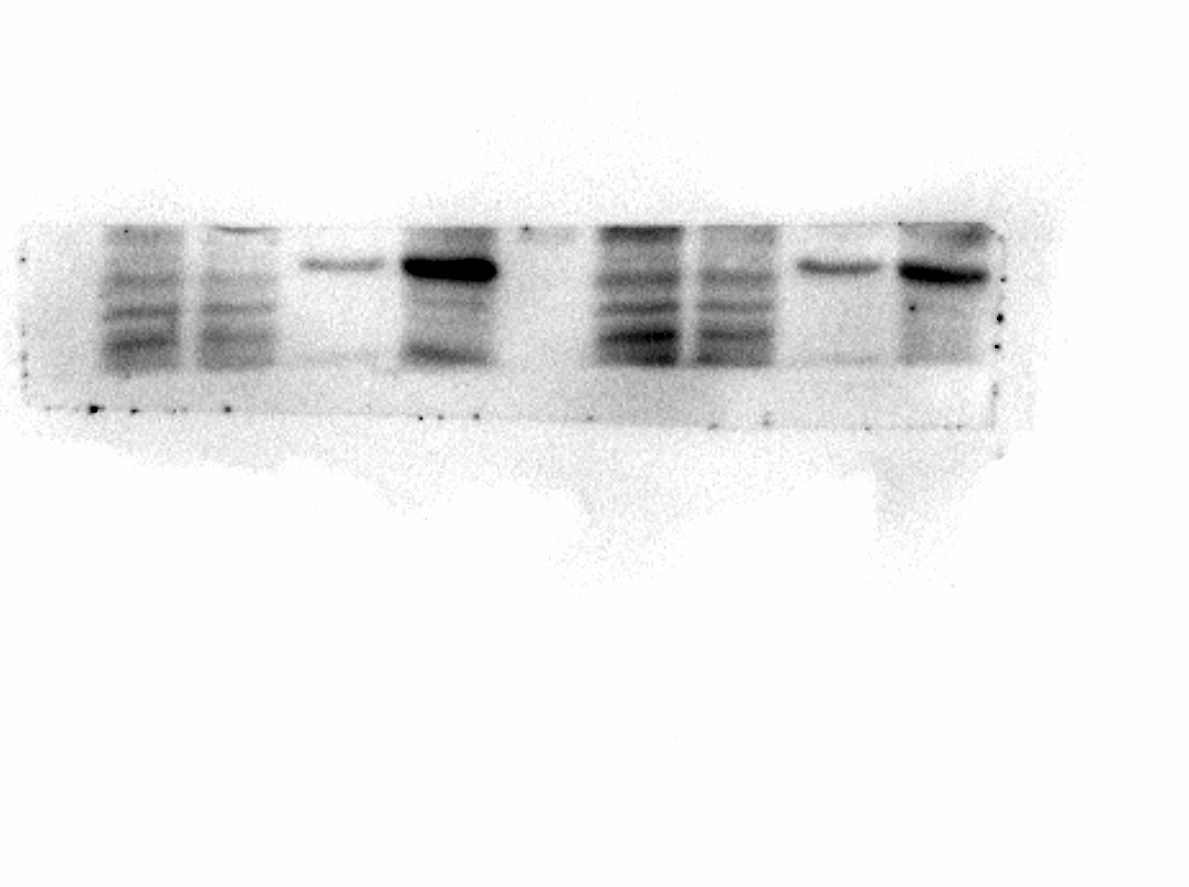


Fig. 3C

Input-ACVRL1


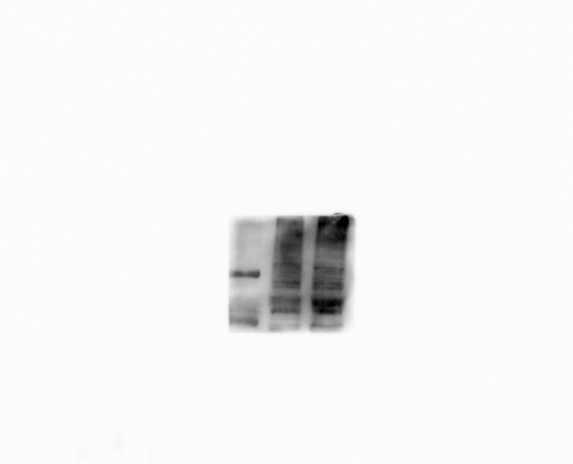


Input-GPX2


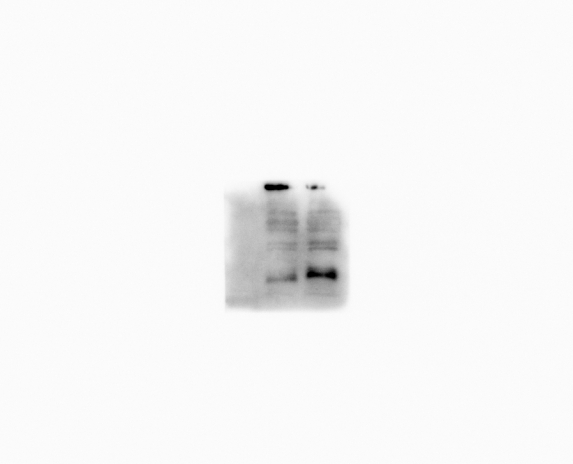


IP-Flag


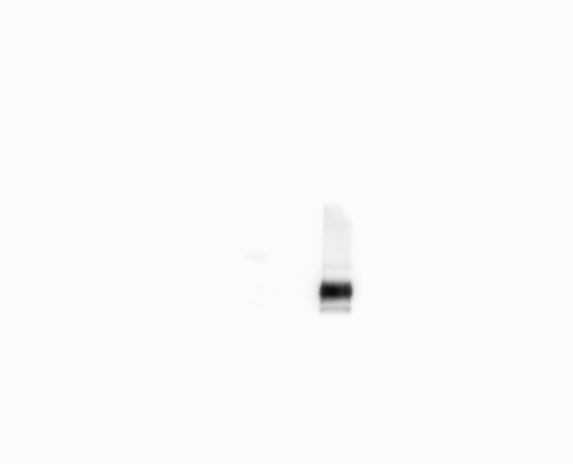


IP-GPX2


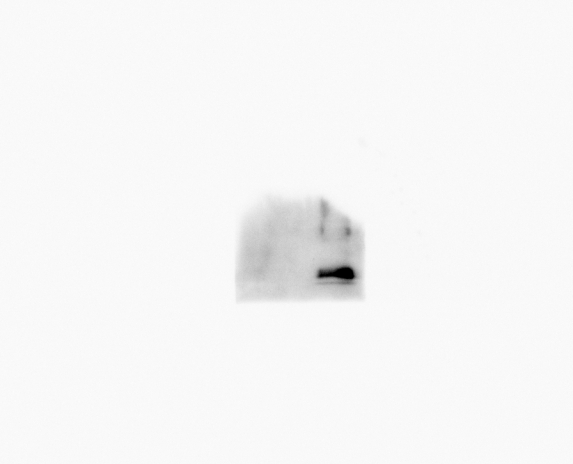


Fig. 3D

IP-ACVRL1:

ACVRL1


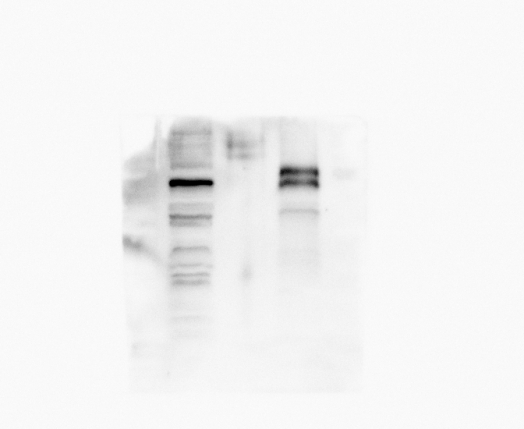


GPX2


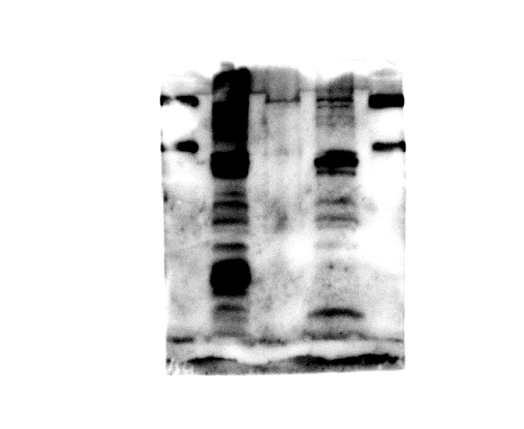


Fig. 3D

IP-GPX2:

ACVRL1


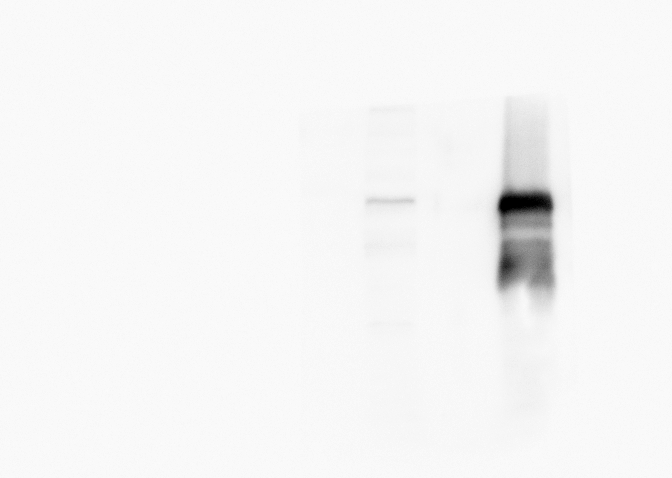


GPX2


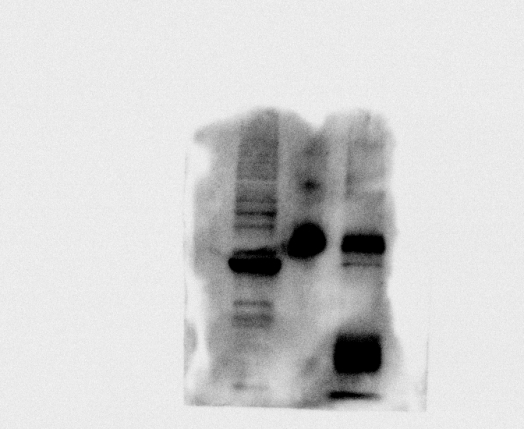


Fig. 3E

IP-ACVRL1:

ACVRL1


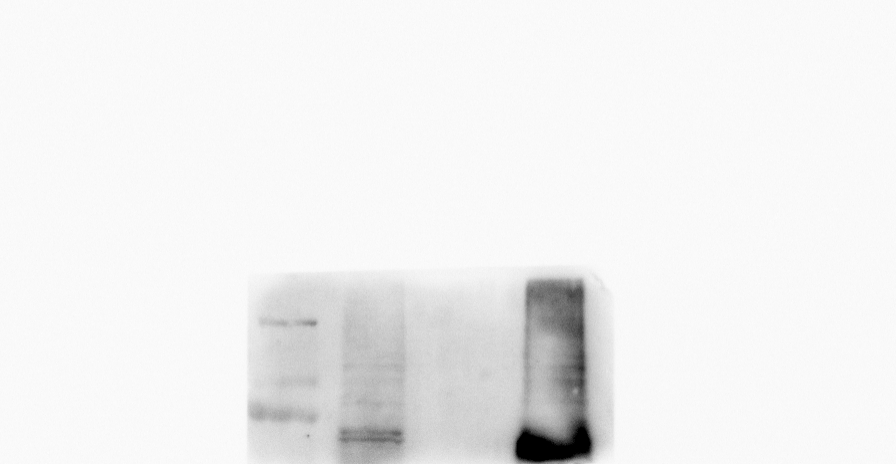


GPX2


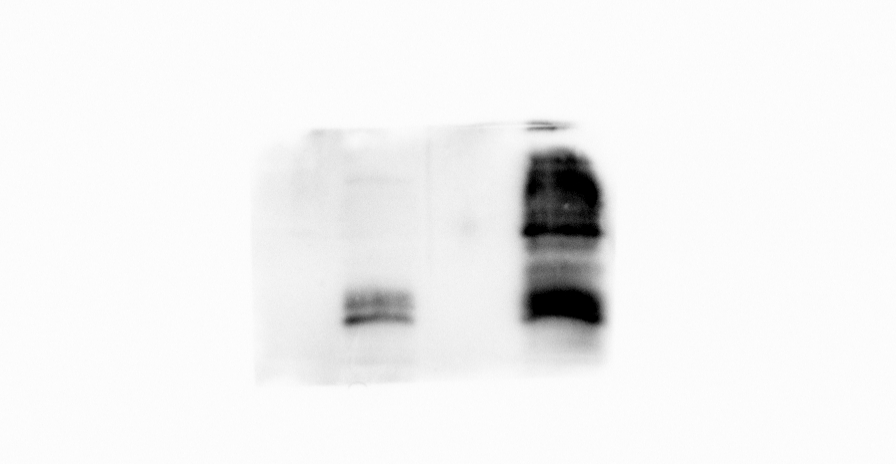


IP-GPX2:

ACVRL1


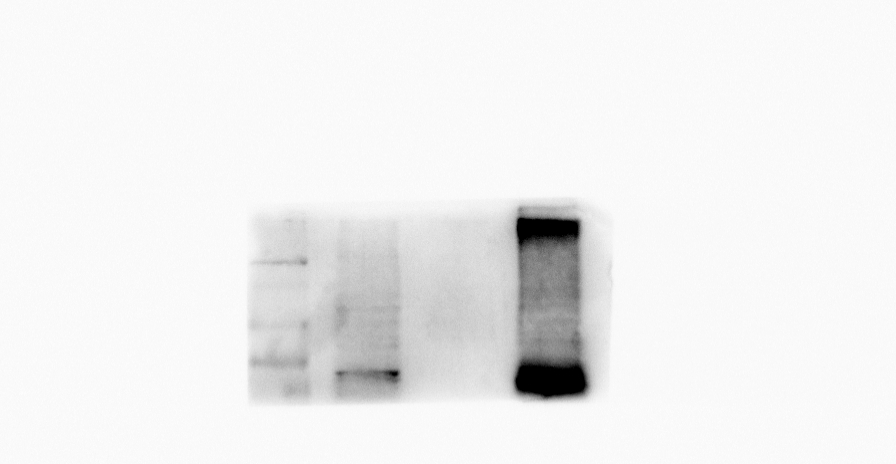


GPX2


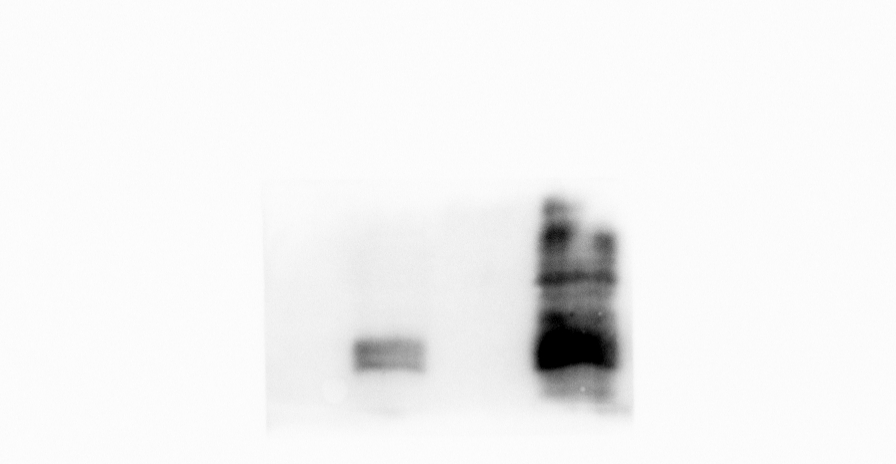


Fig. 3G

GPX2


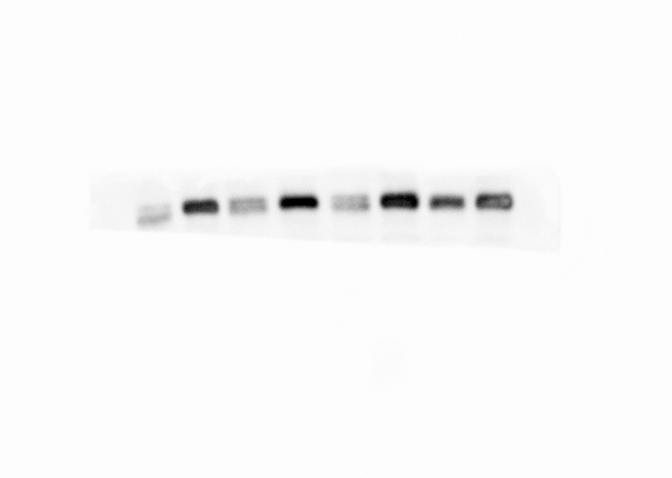


ACVRL1


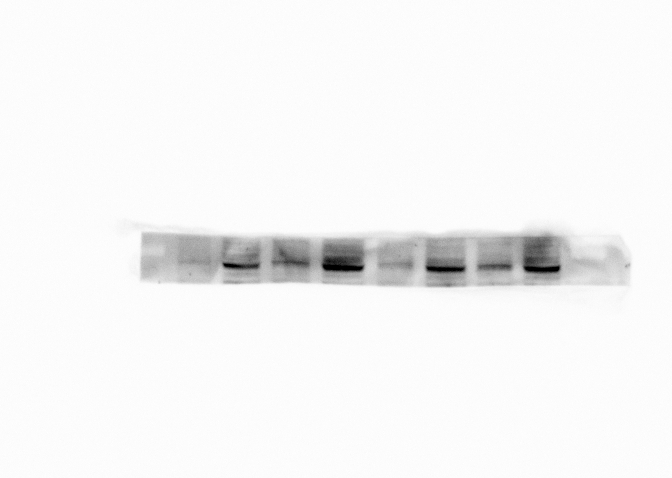


ACTIN


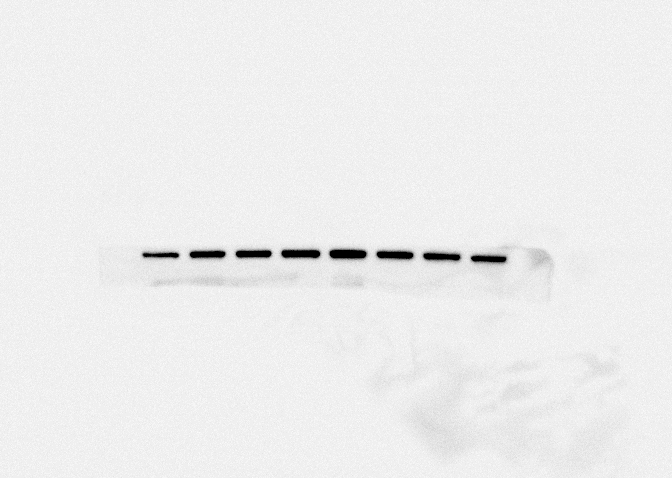


Fig. 3I

LS174T

IP:Flag





IP:GPX2


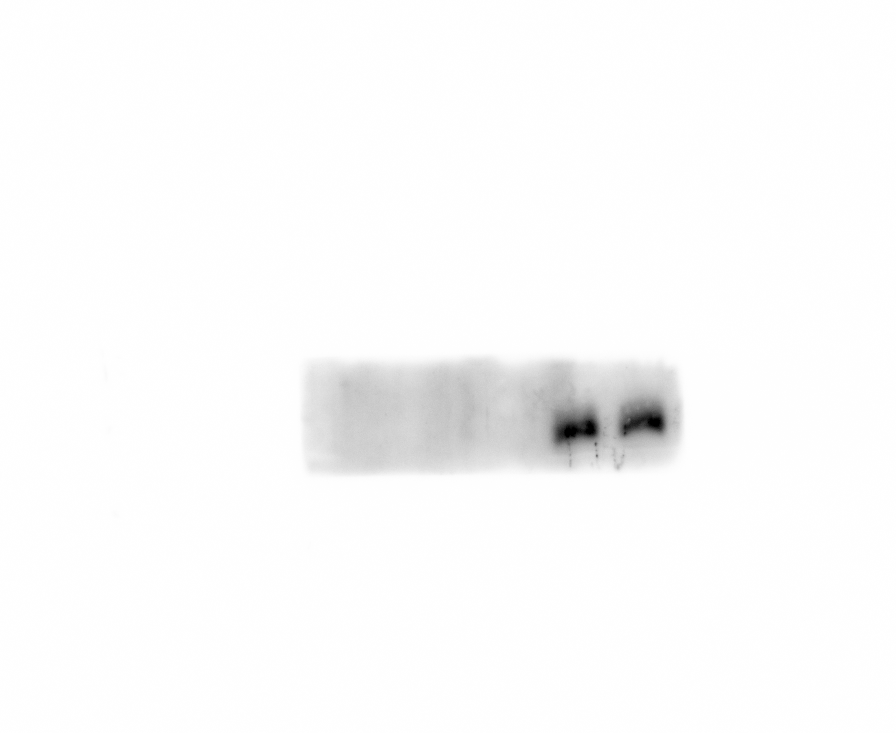


Input-Flag





Input-GPX2





HCT15

IP:Flag





IP:GPX2





Input-Flag





Input-GPX2





Fig. 4A

HCT15:

ACTIN


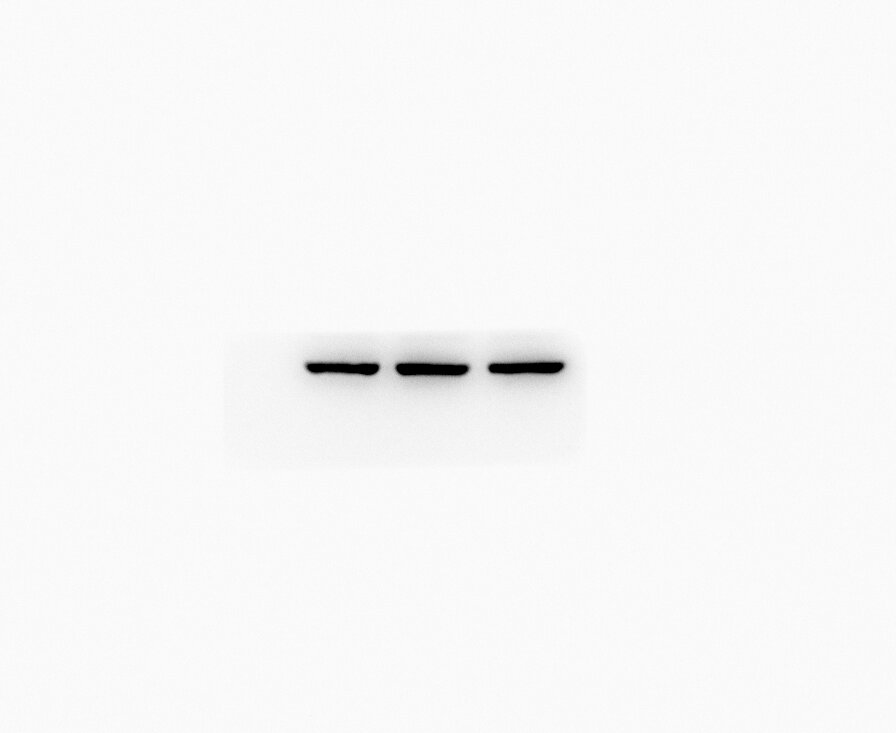


ACVRL1


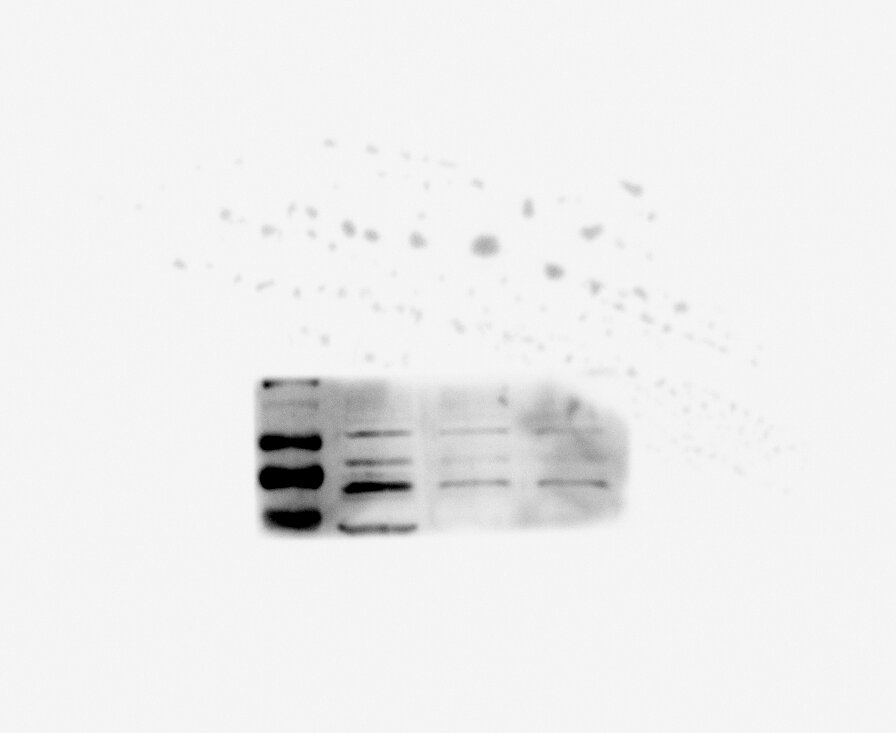


GPX2


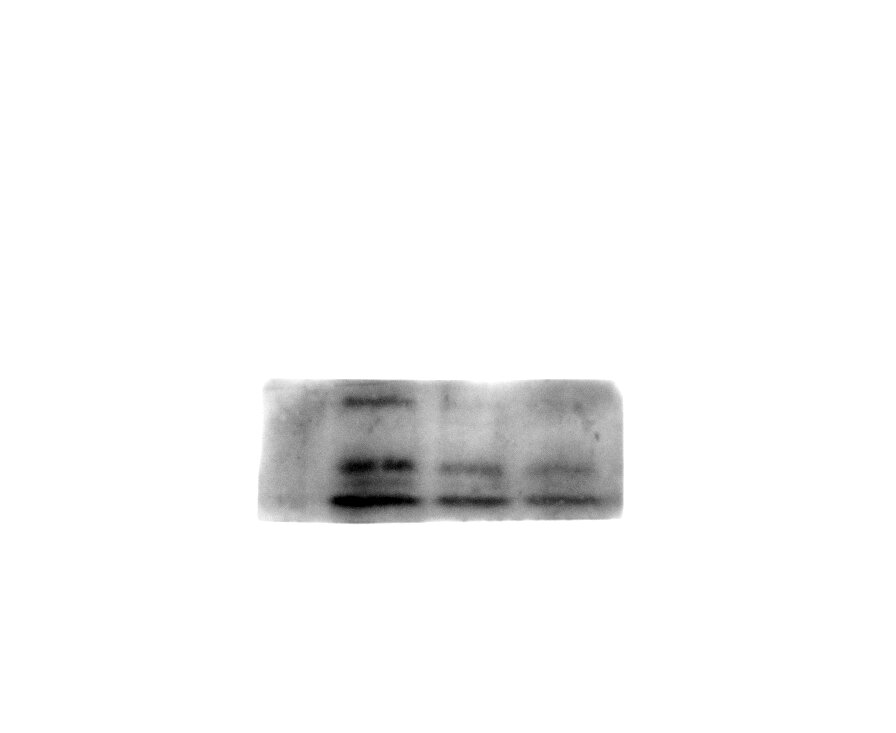


LS174T:

ACTIN


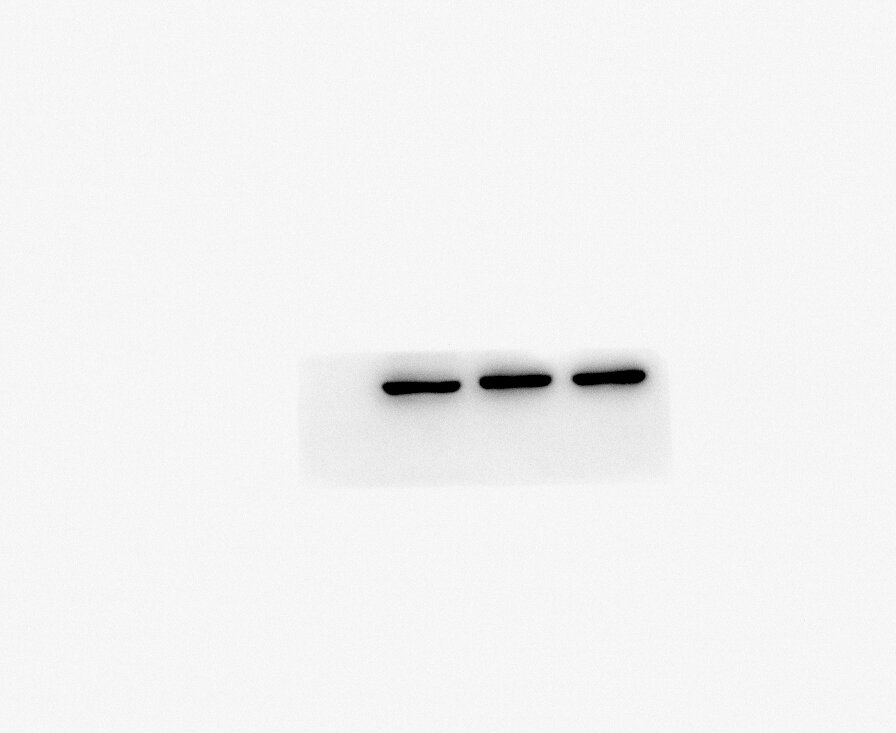


ACVRL1


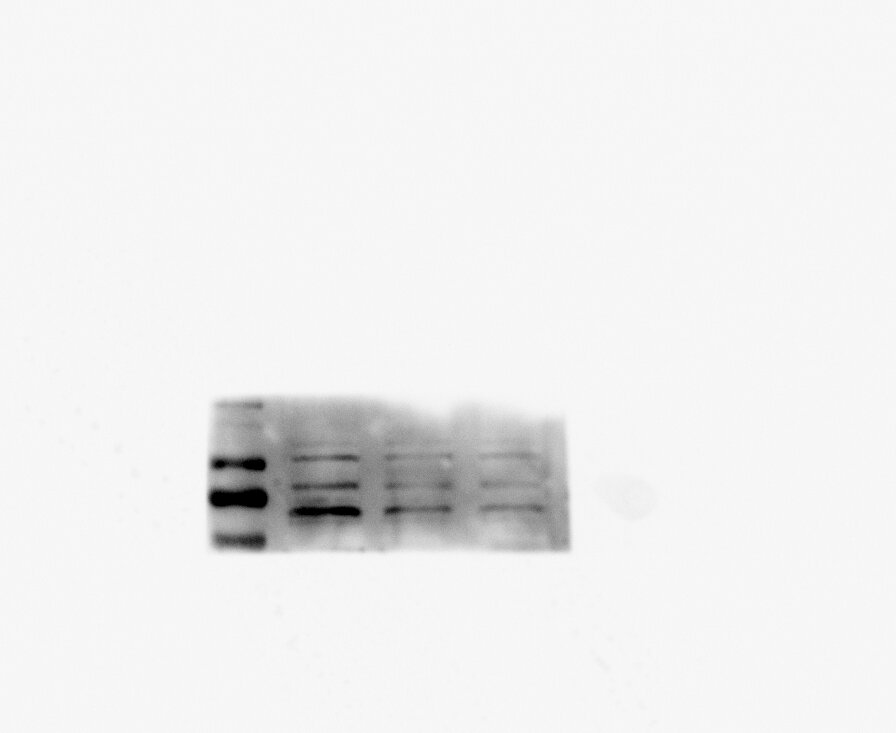


GPX2


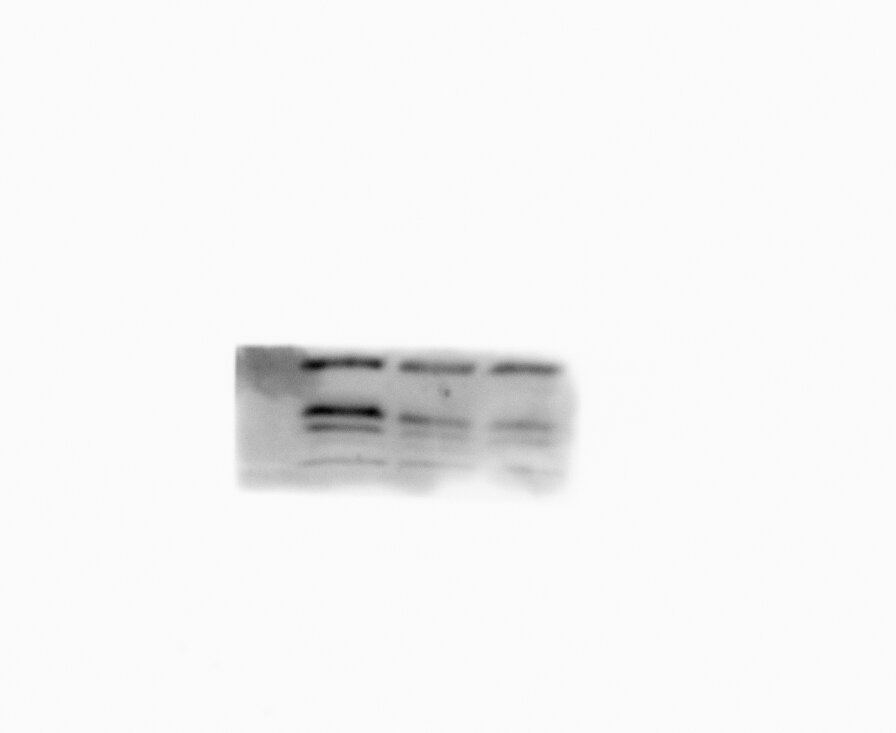


Fig. 4B

HCT15:

ACTIN


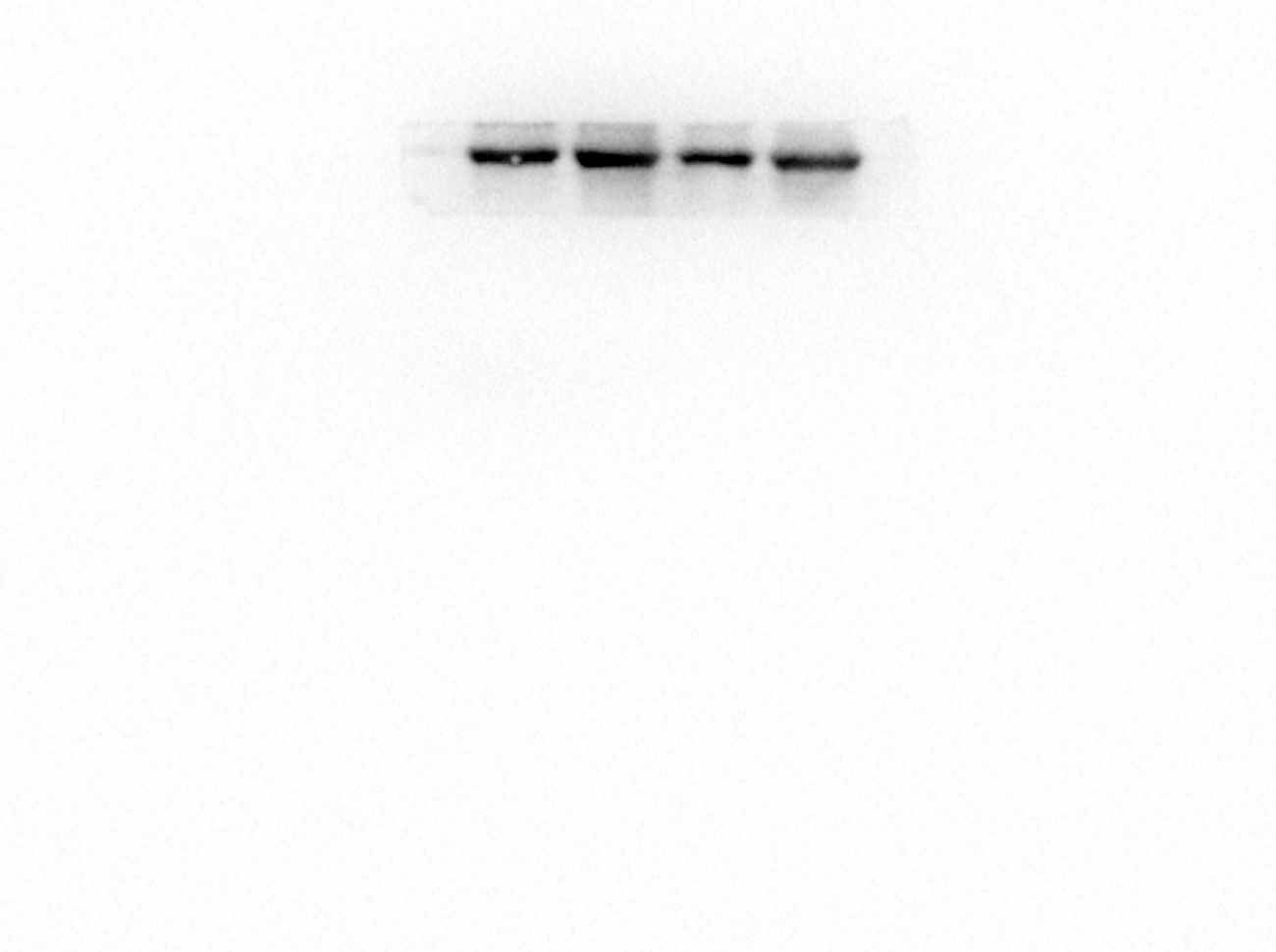


GPX2


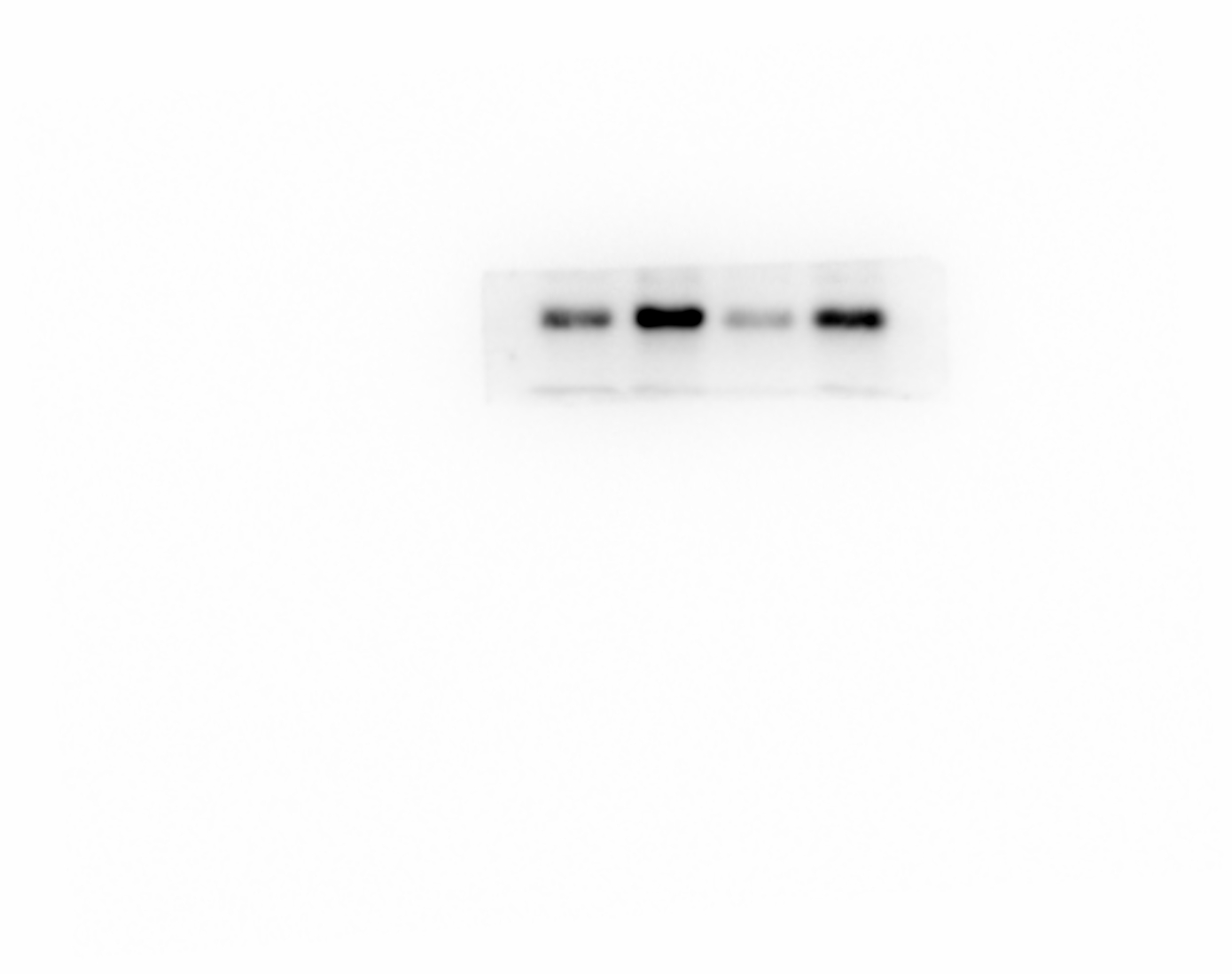


LS174T:

ACTIN


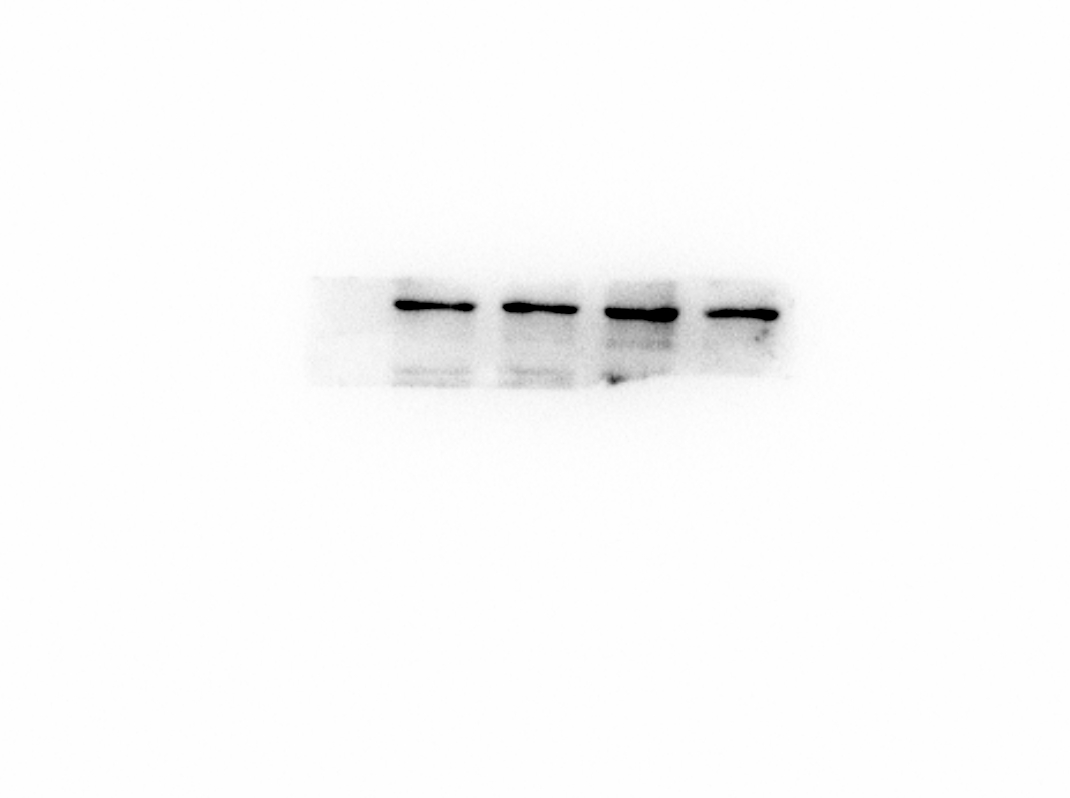


GPX2


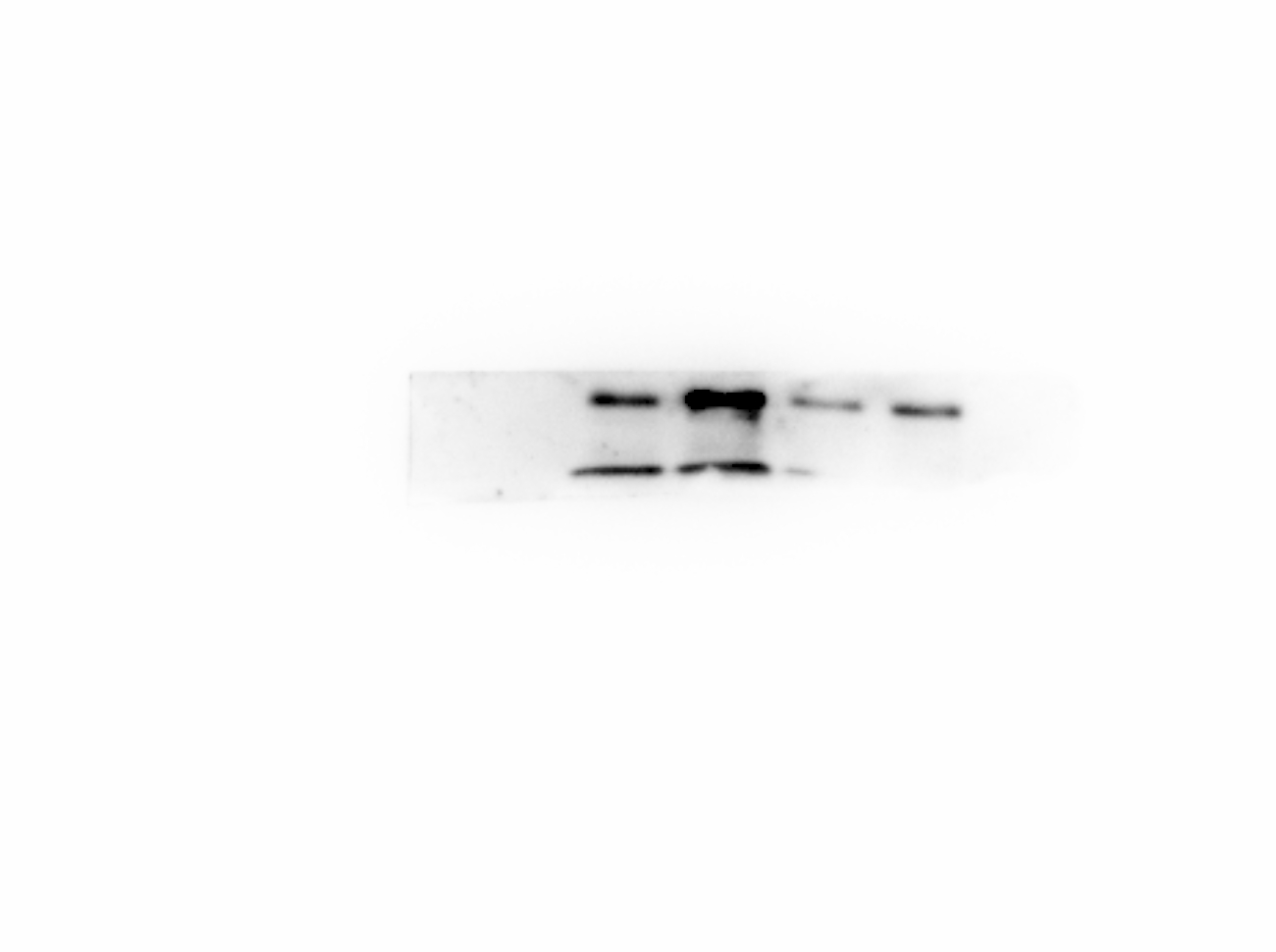


Fig. 4C

shACVRL1:

ACTIN


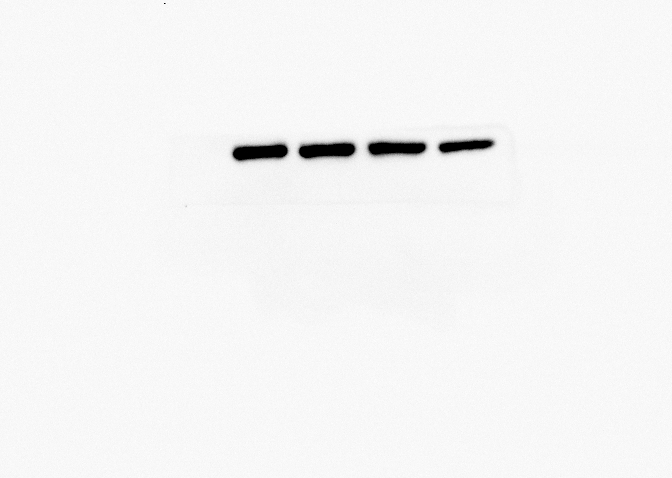


GPX2


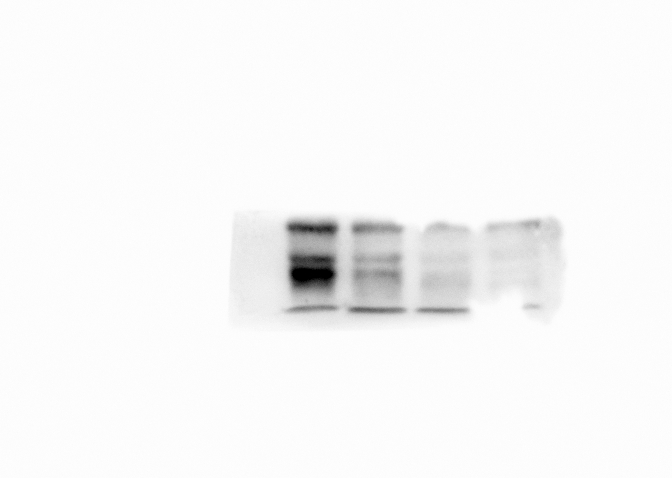


shNTC:

ACTIN


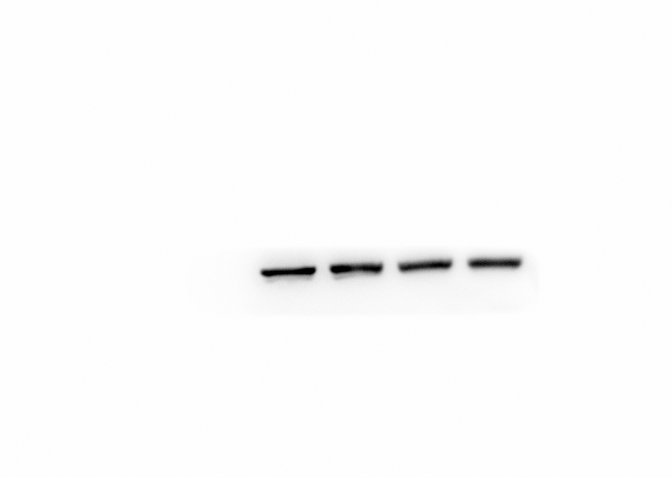


GPX2


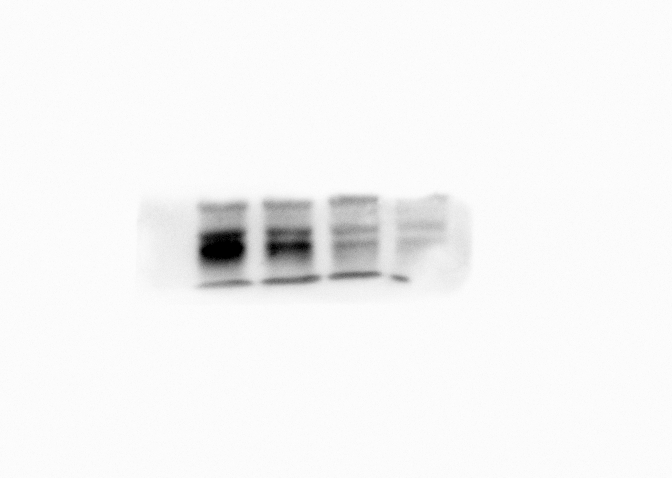


Fig. 4D

ACTIN(shNTC-Lane1-4 shACVRL1-Lane6-9)


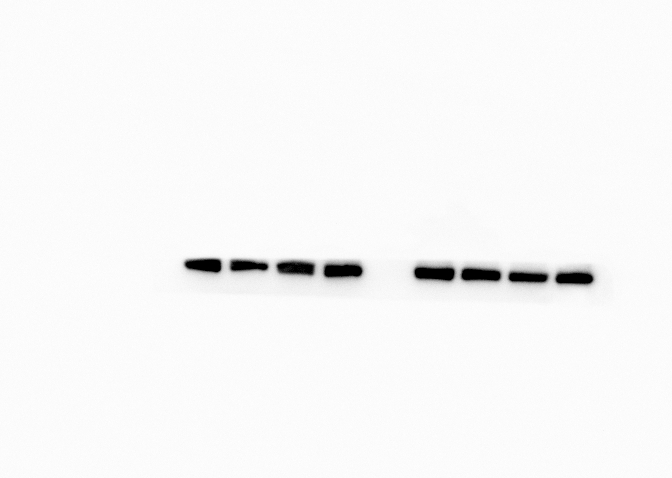


GPX2(shNTC-Lane1-4 shACVRL1-Lane6-9)


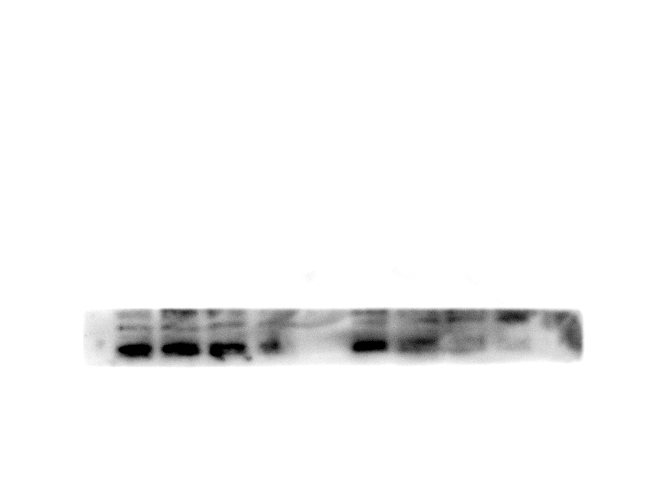


Fig. 4E

IP:ACVRL1

ACVRL1


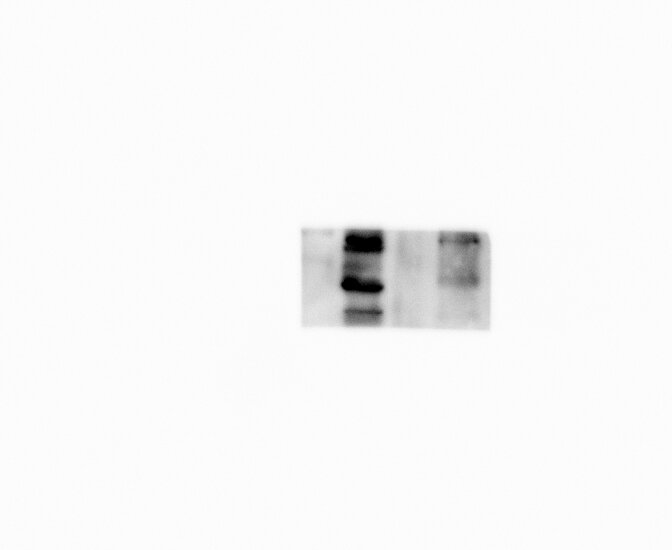


USP15


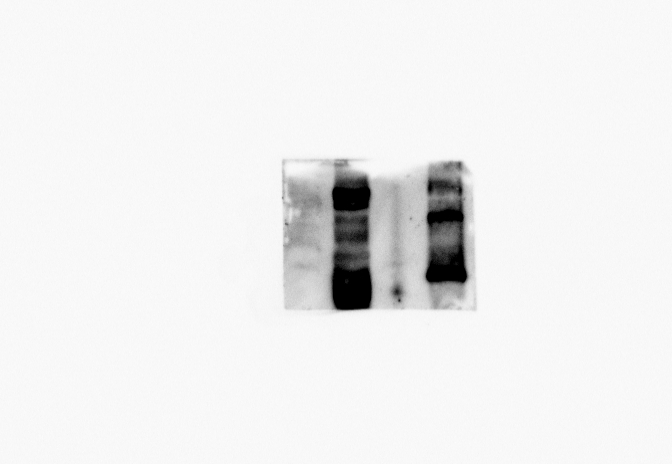


IP:USP15

ACVRL1


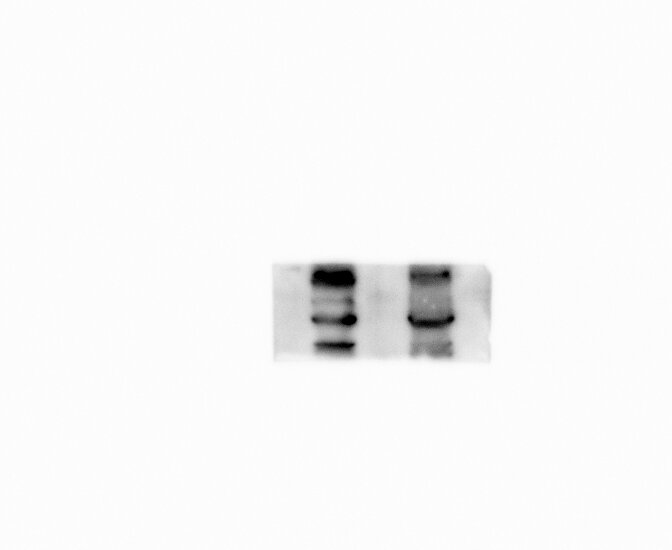


USP15


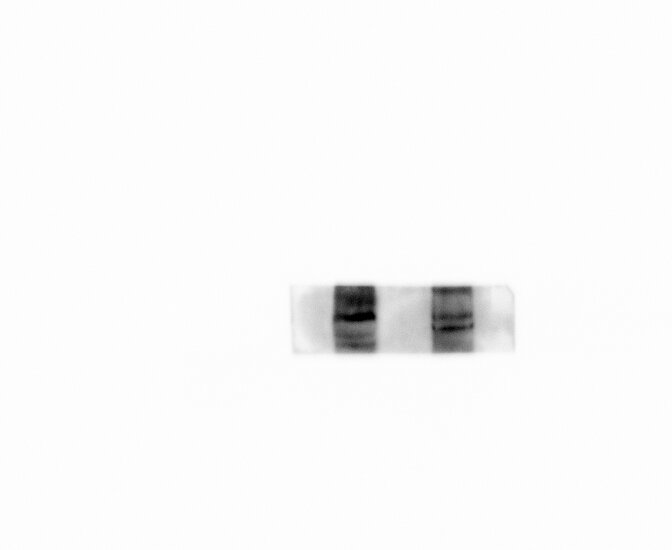


Fig. 4F

IP:GPX2

GPX2


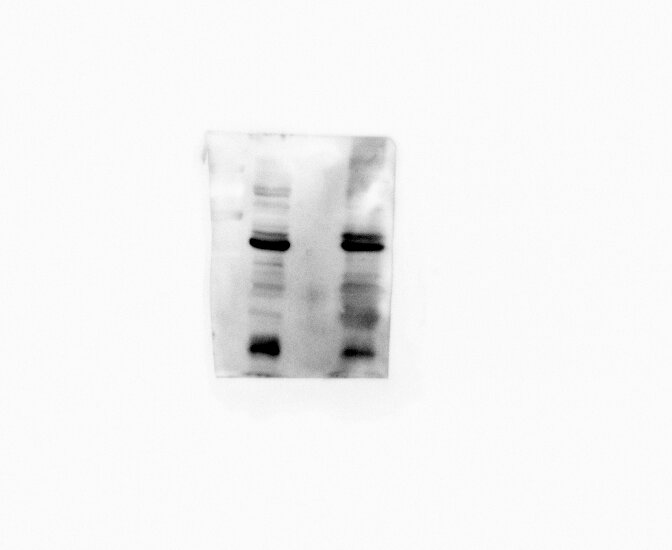


USP15


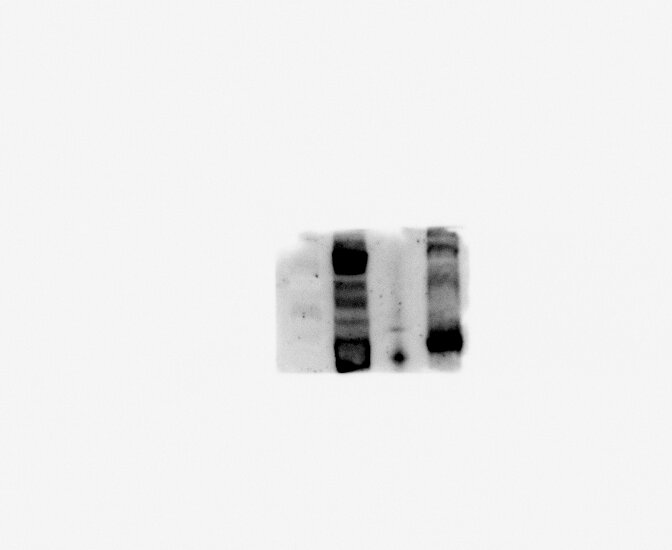


IP:USP15

GPX2


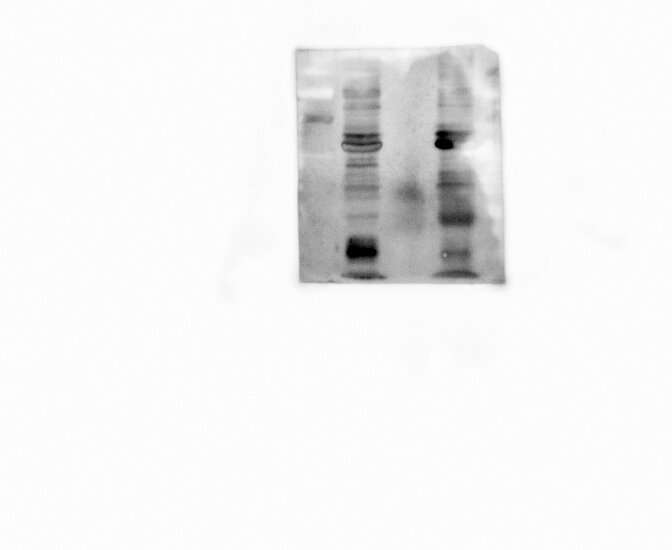


USP15


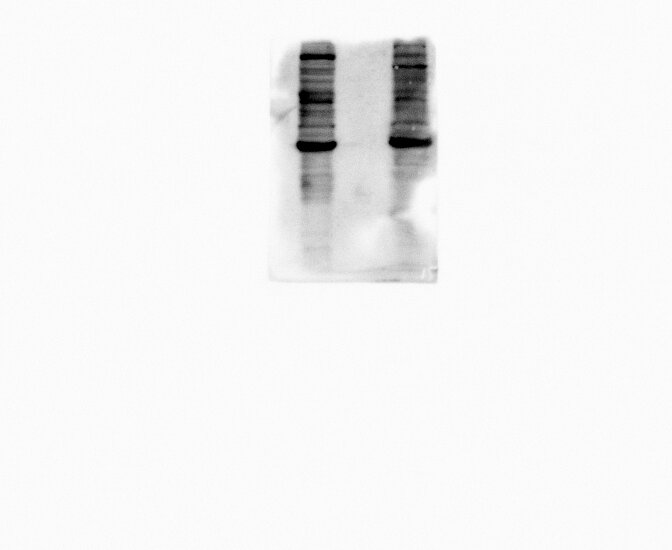


Fig. 4G

HCT15-IP

GPX2


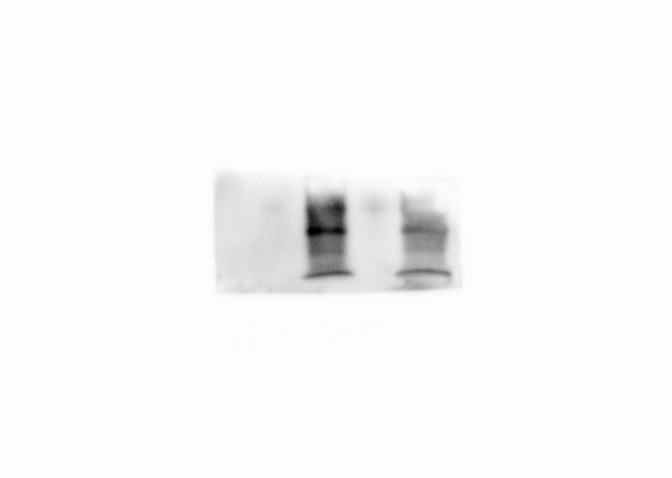


USP15


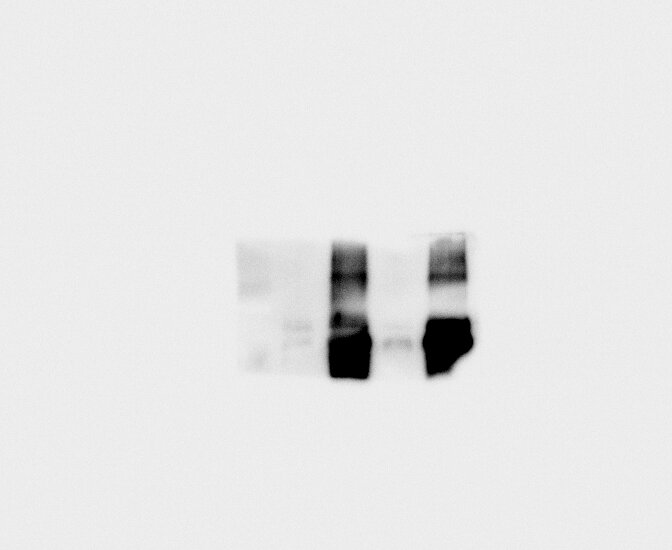


HCT15-Input

ACTIN


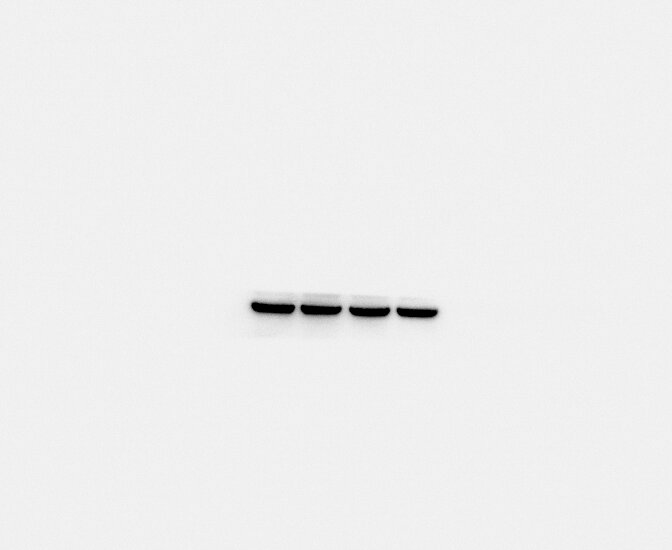


ACVRL1


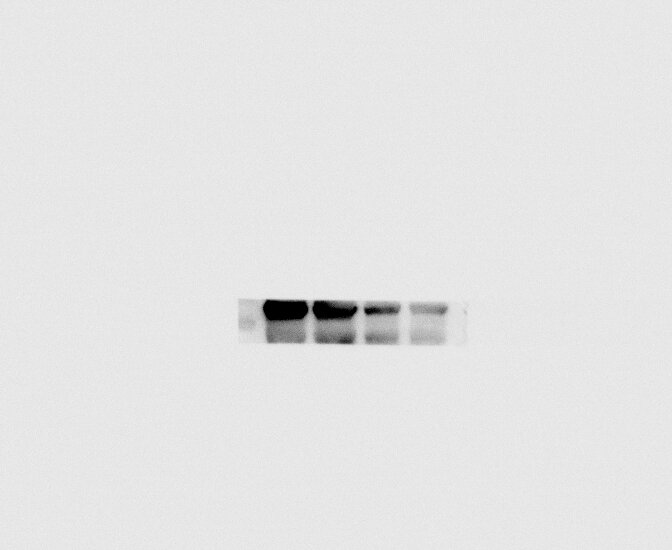


GPX2


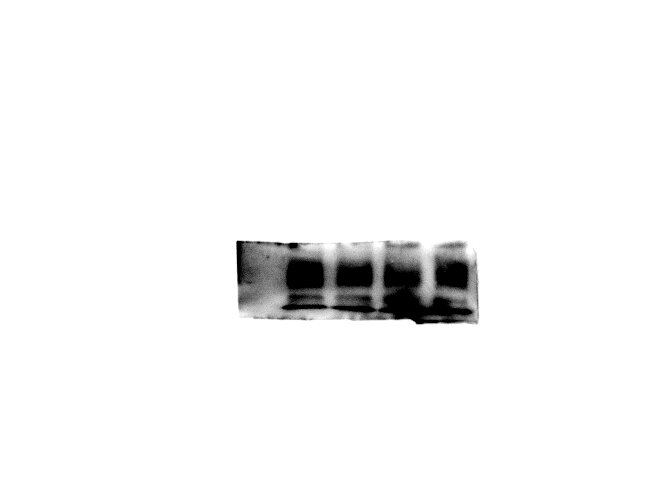


LS174T-IP

GPX2


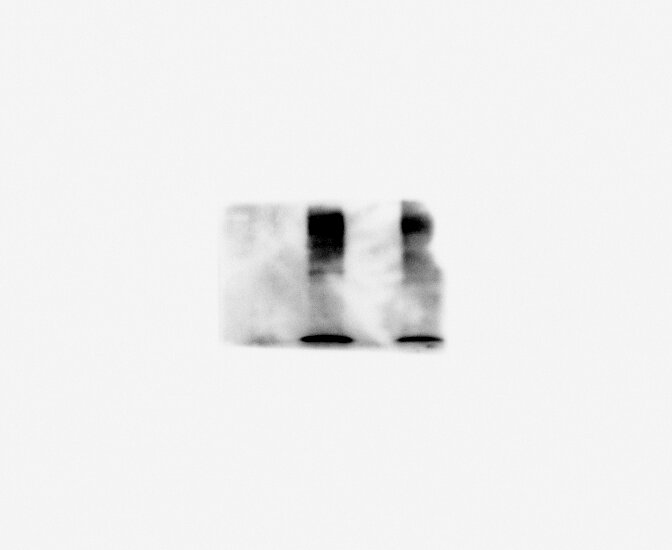


USP15


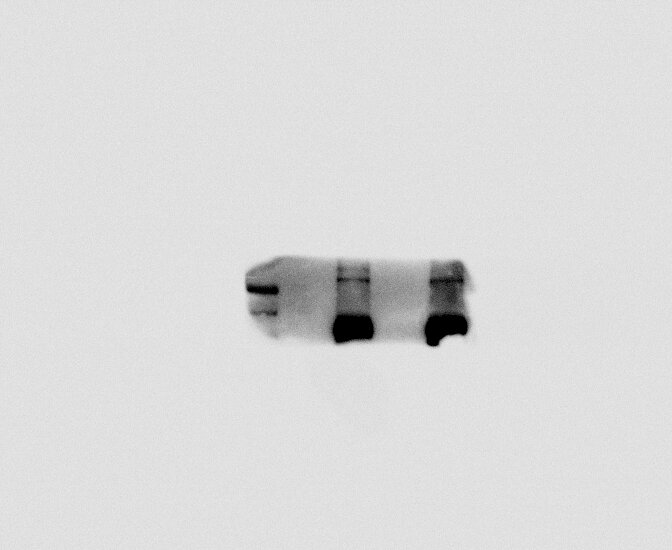


LS174T-Input

ACTIN


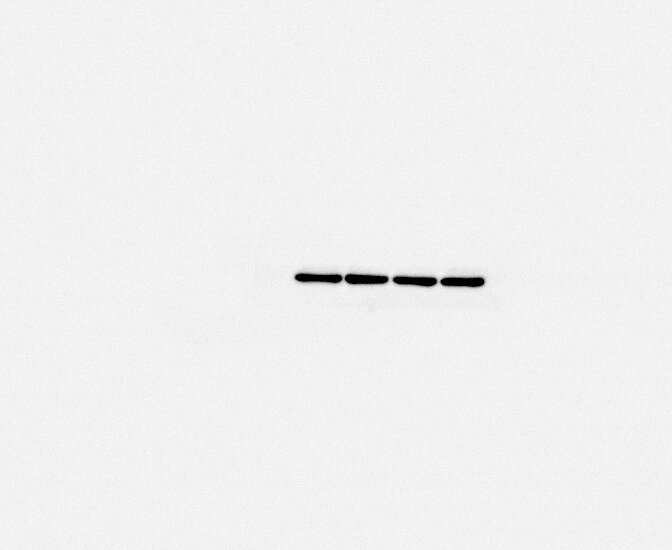


ACVRL1


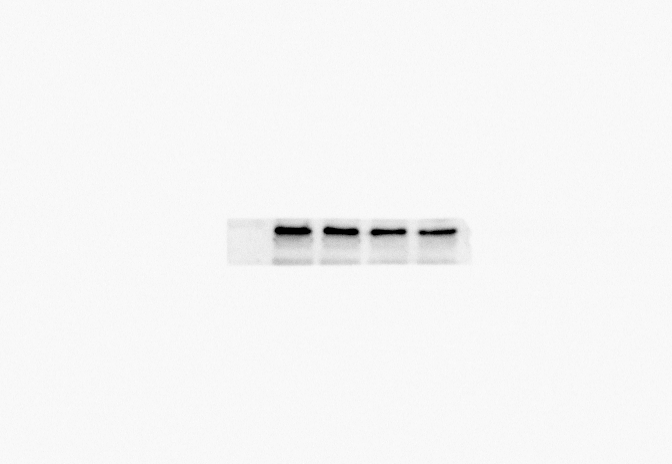


GPX2


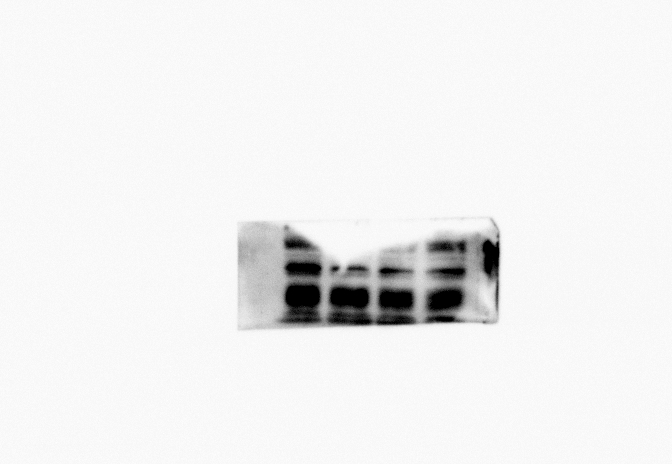


Fig.4H

HCT15:

ACTIN


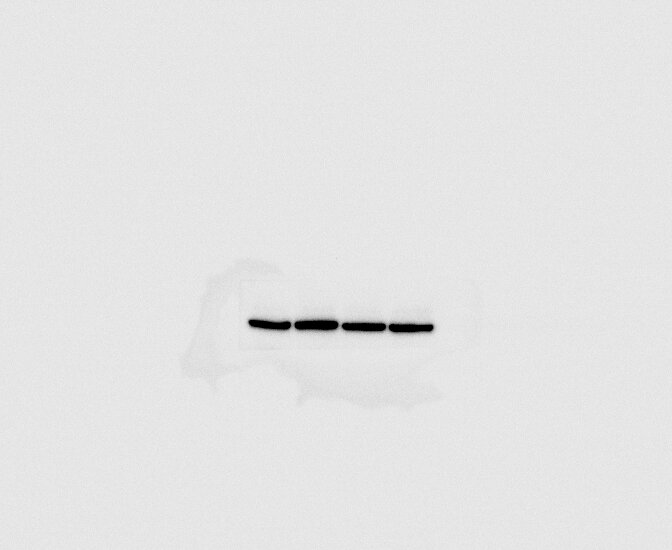


GPX2


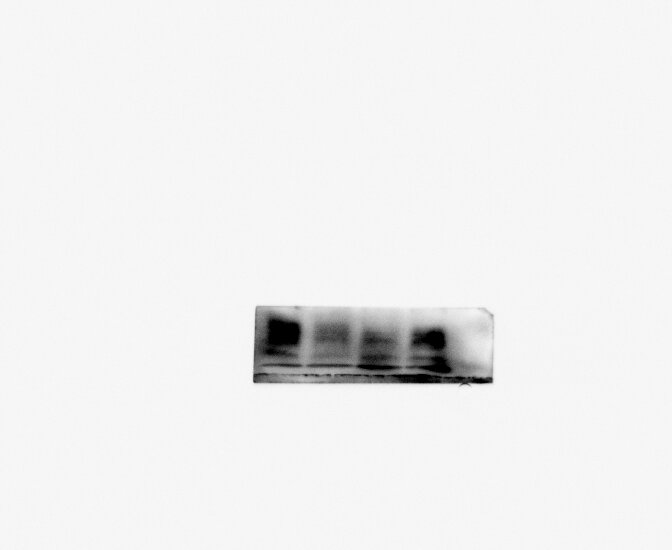


USP15


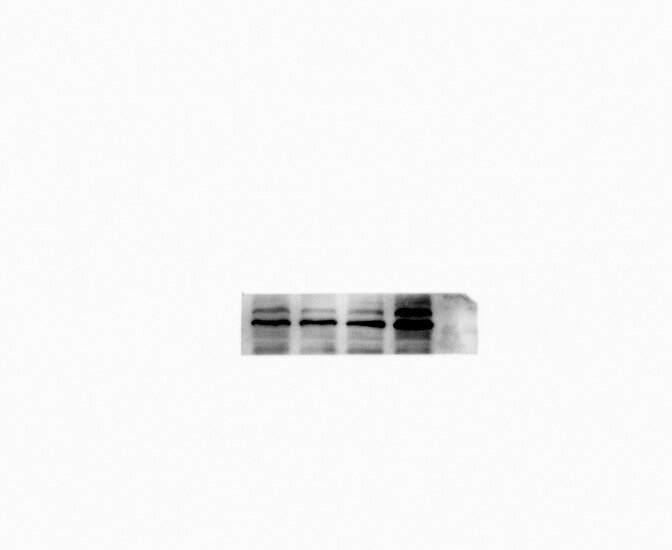


LS174T:

ACTIN


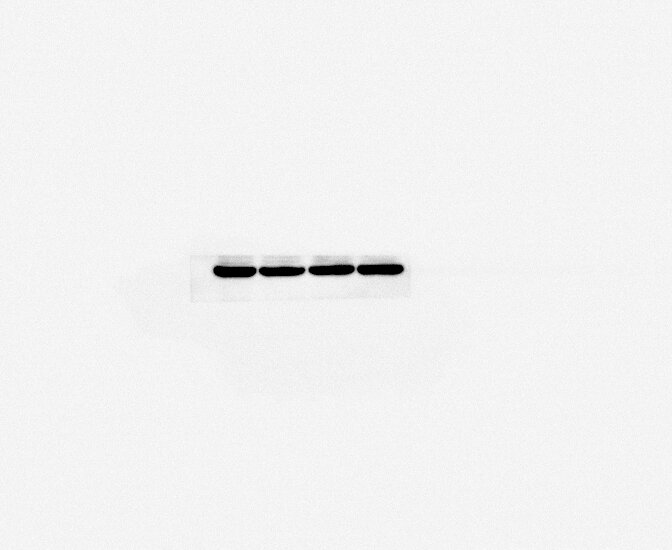


GPX2


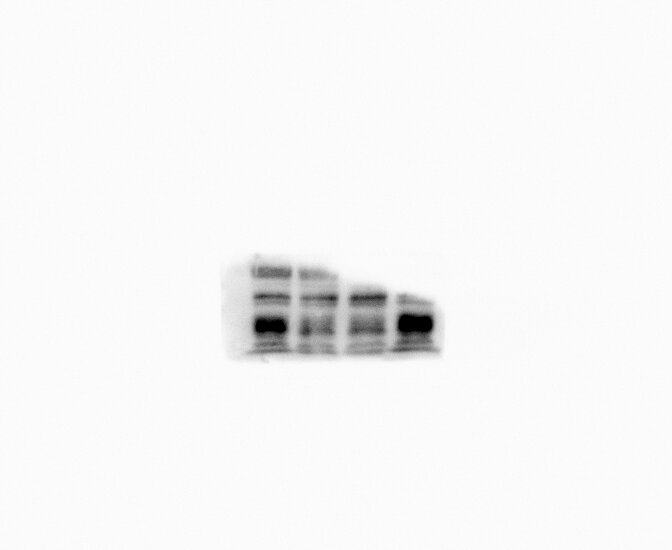


USP15


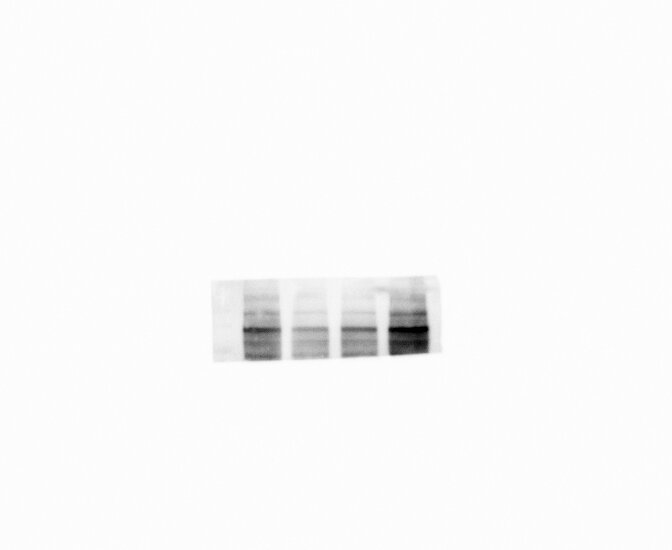


Fig.4I

HCT15

IP-HA


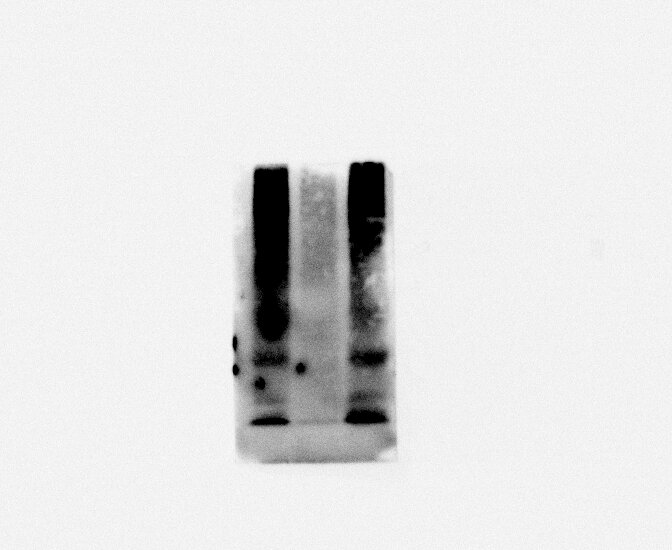


Input:

ACTIN


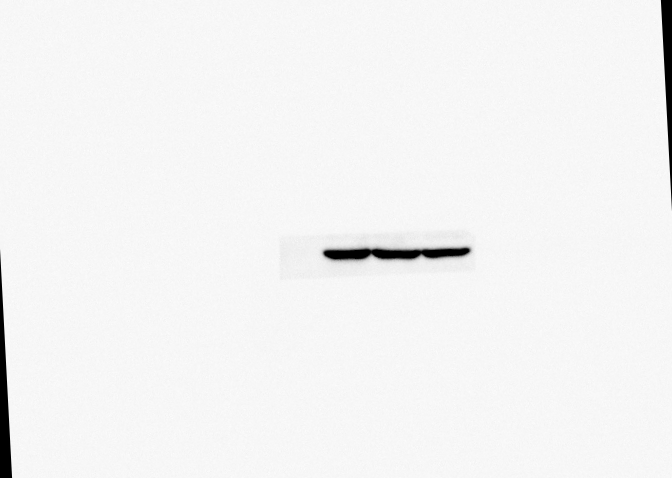


ACVRL1


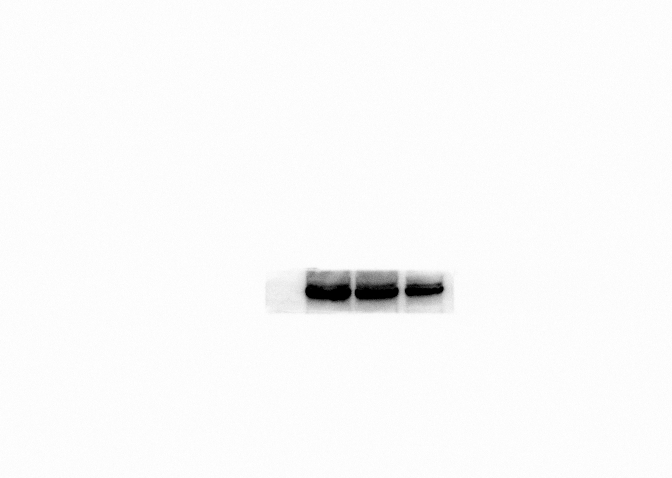


GPX2


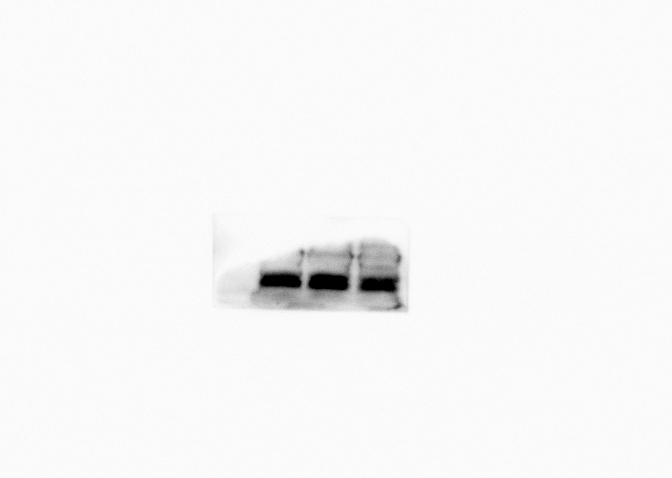


USP15


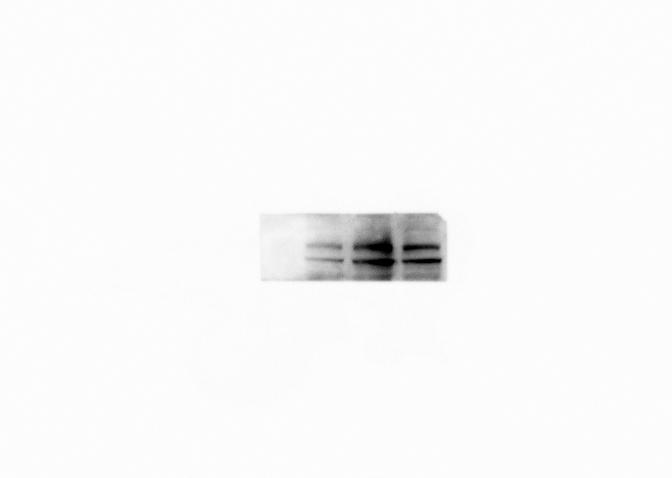


LS174T

IP-HA


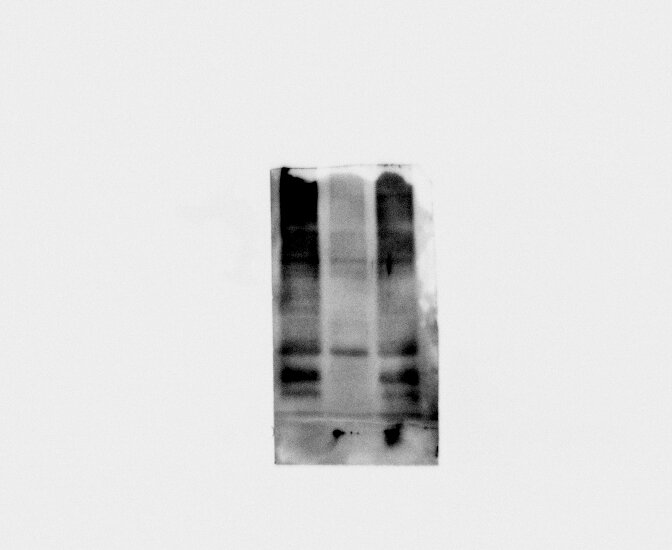


Input:

ACTIN


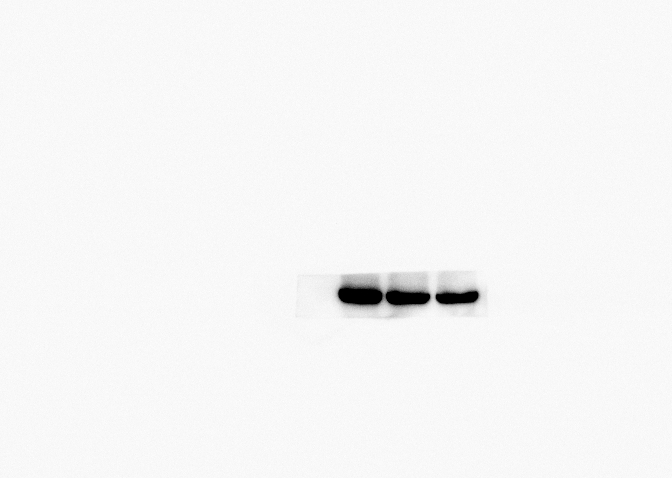


ACVRL1


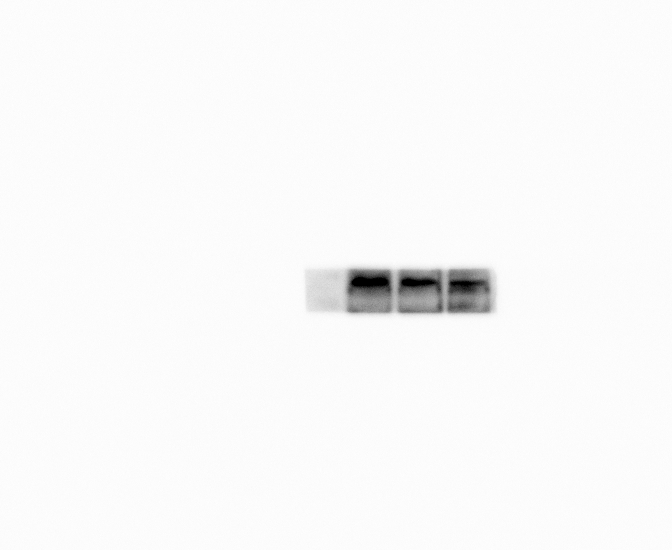


GPX2


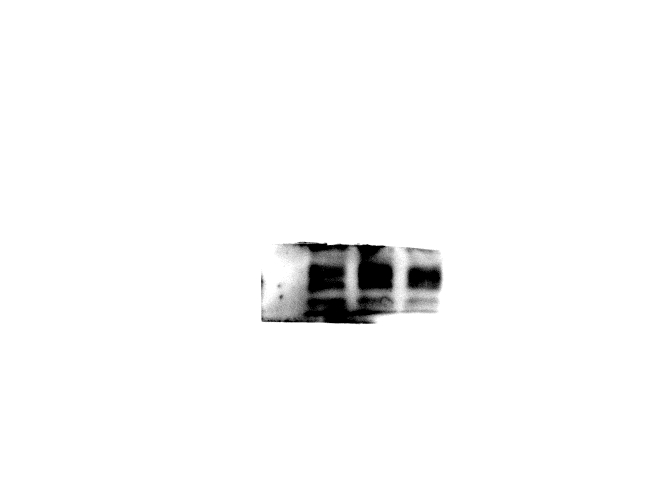


USP15


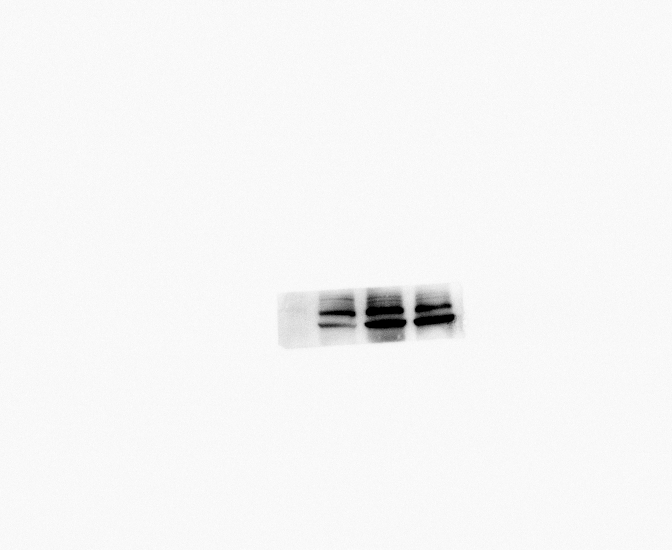


Fig.4J

IP:Myc


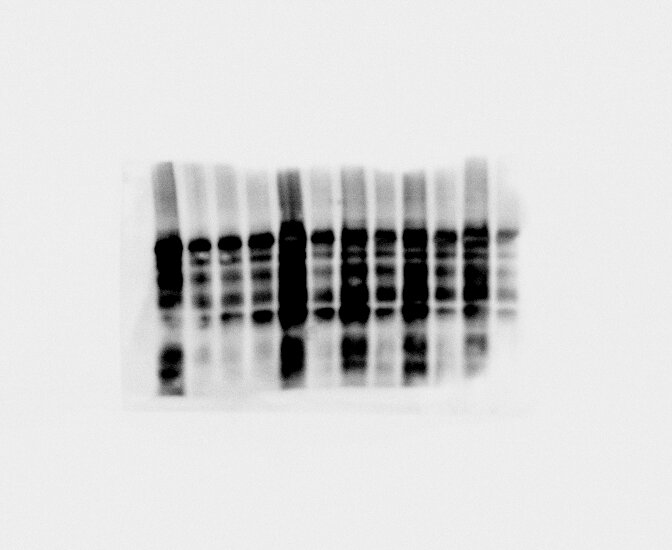


Input

ACTIN


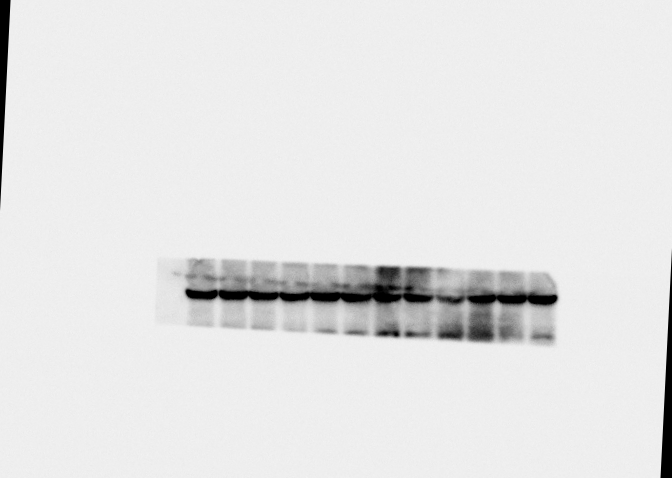


HA


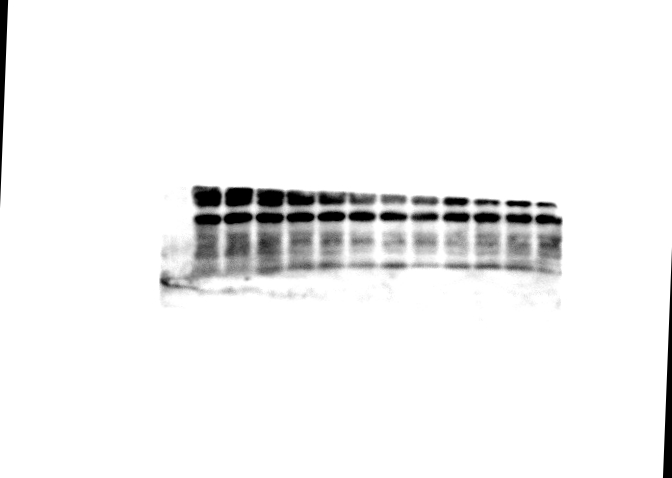


USP15


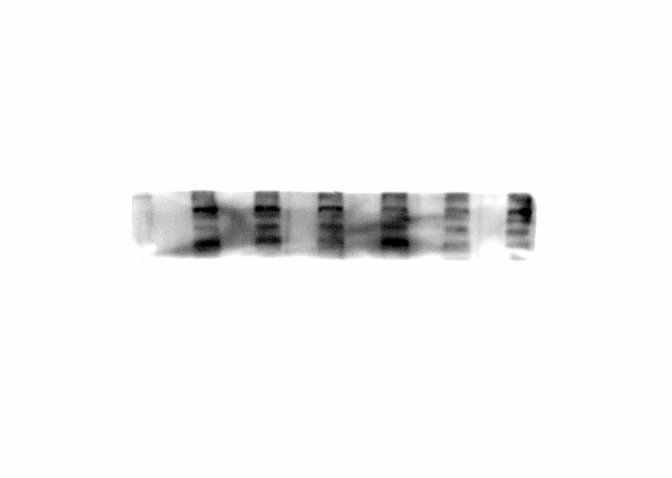


Fig.5A

HCT116

ACTIN(Lane1-2)


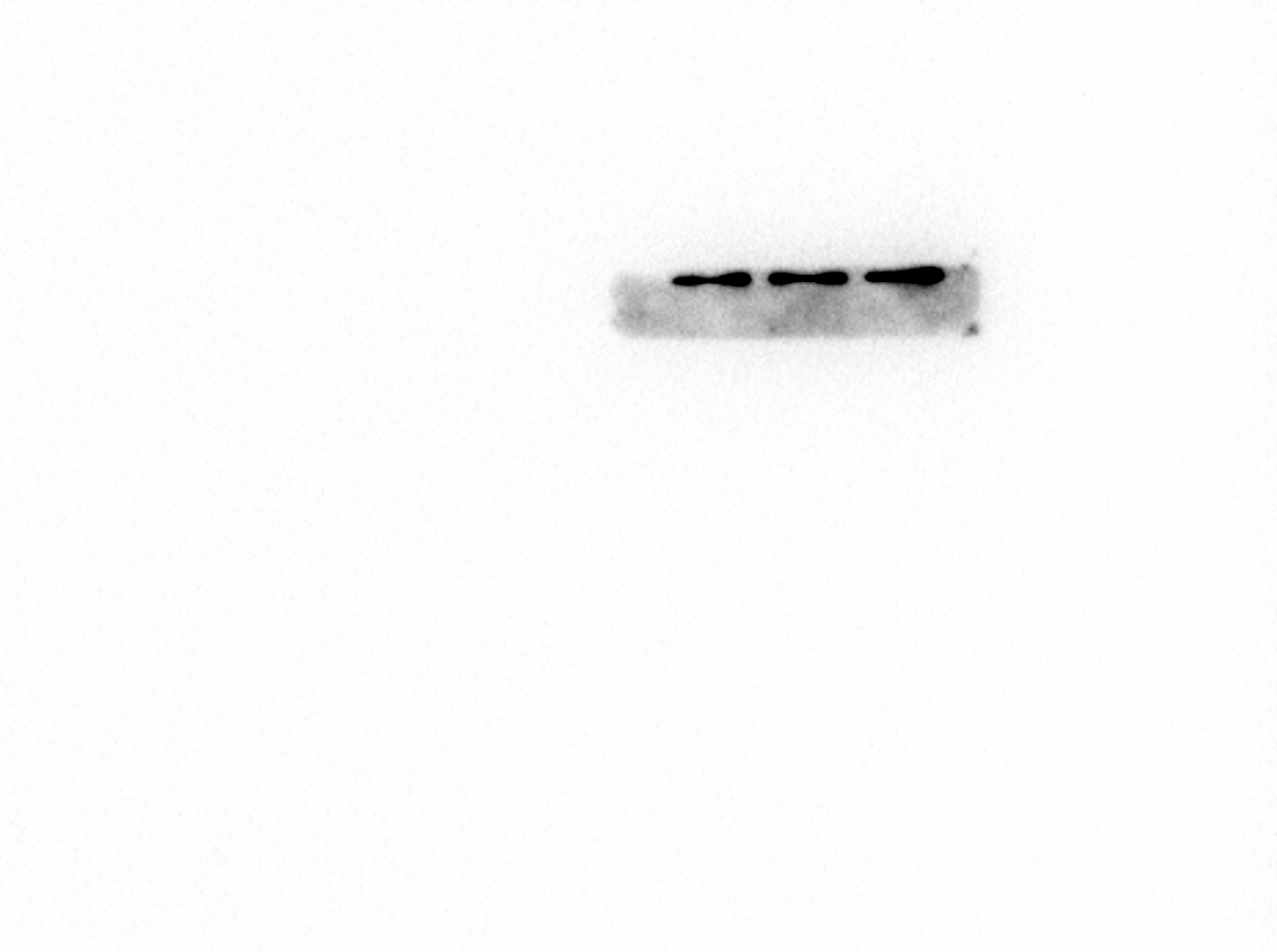


GPX2(Lane1-2)


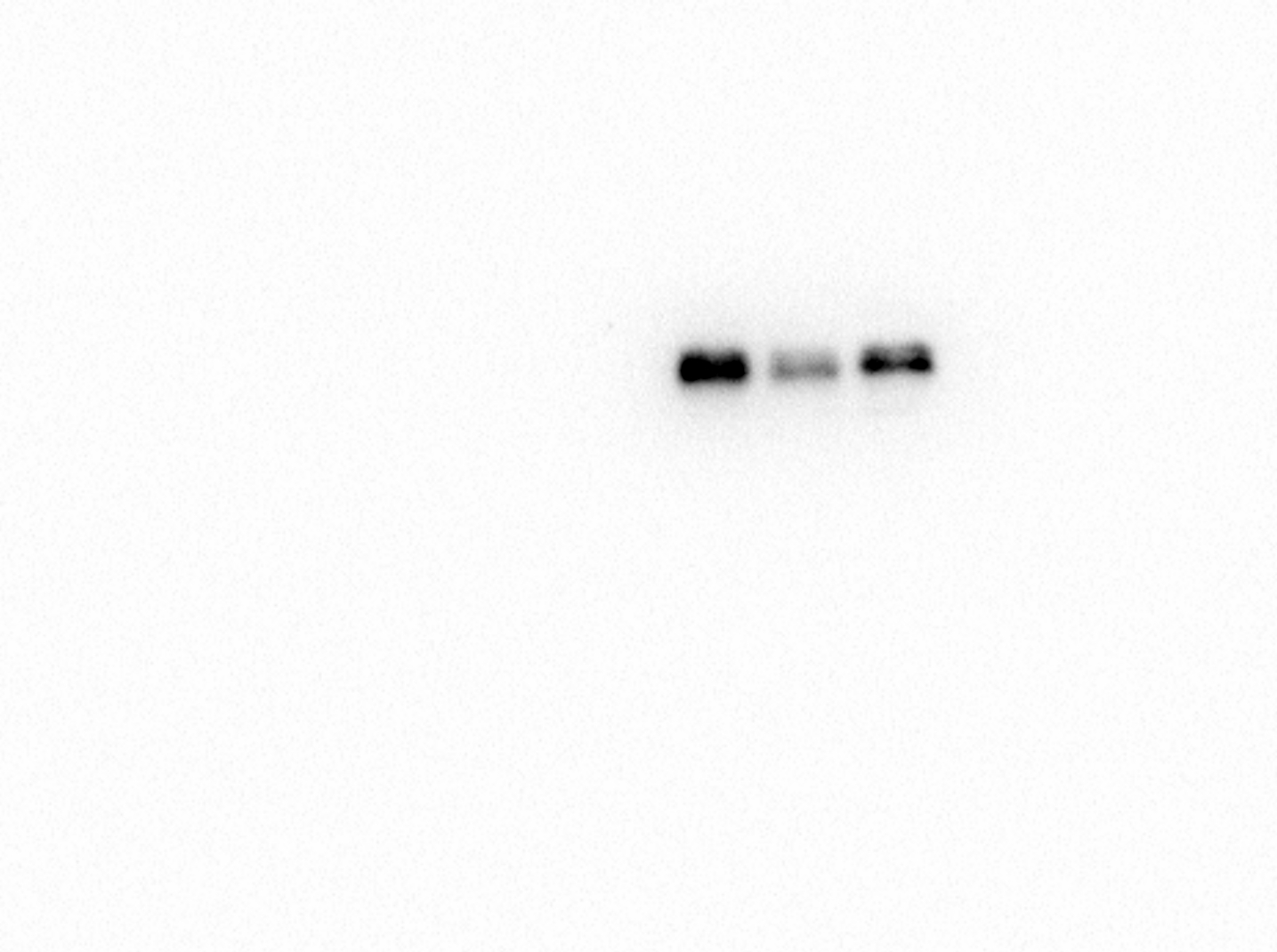


HCT15

ACTIN


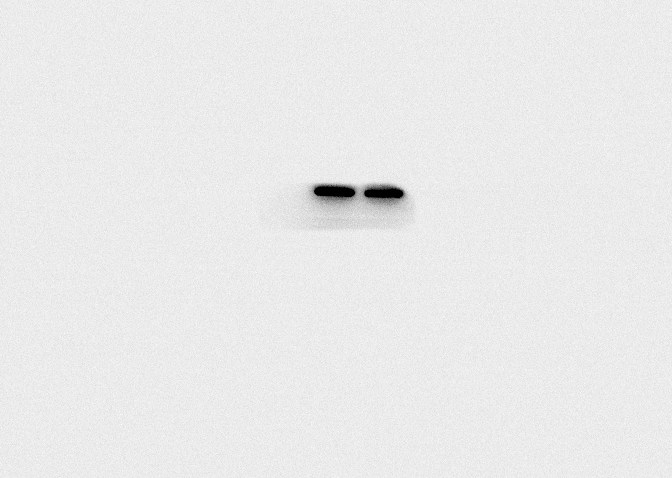


GPX2


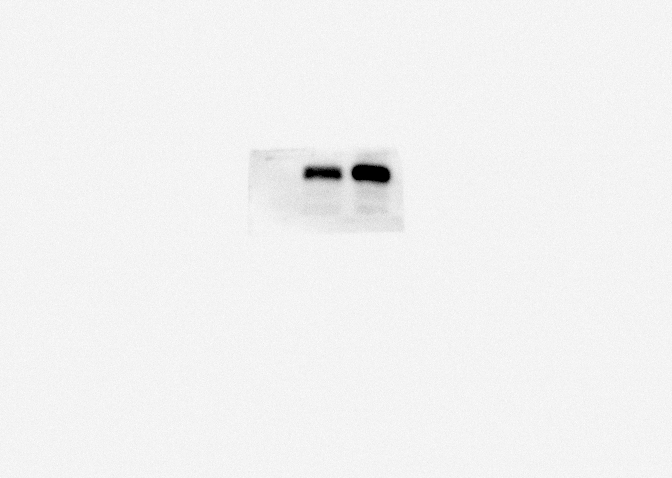


Fig.5B

ACTIN


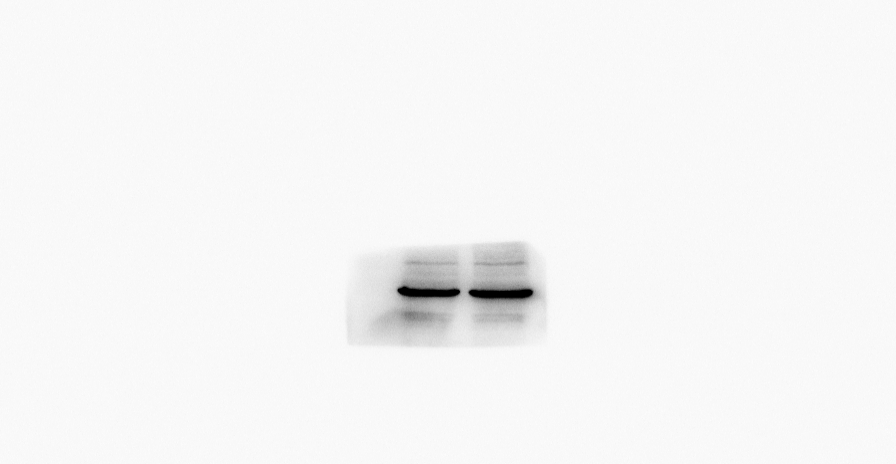


GPX2


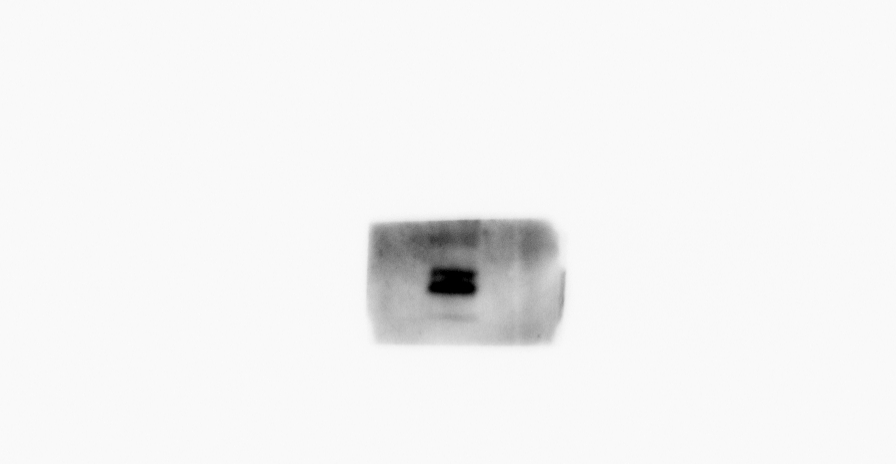


Fig.6B

Licl:

ACTIN


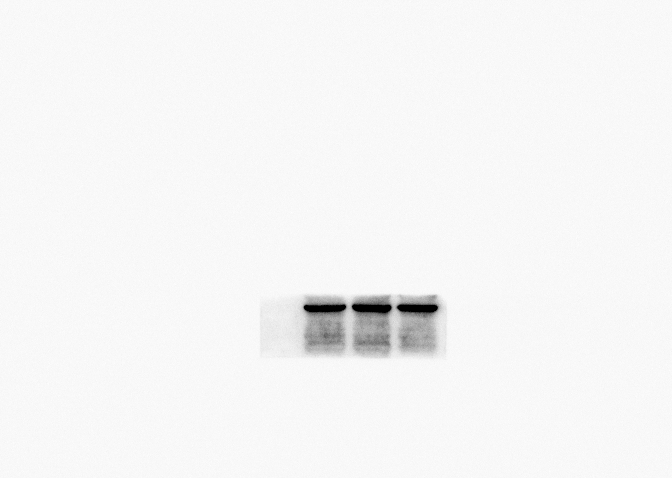


ACVRL1


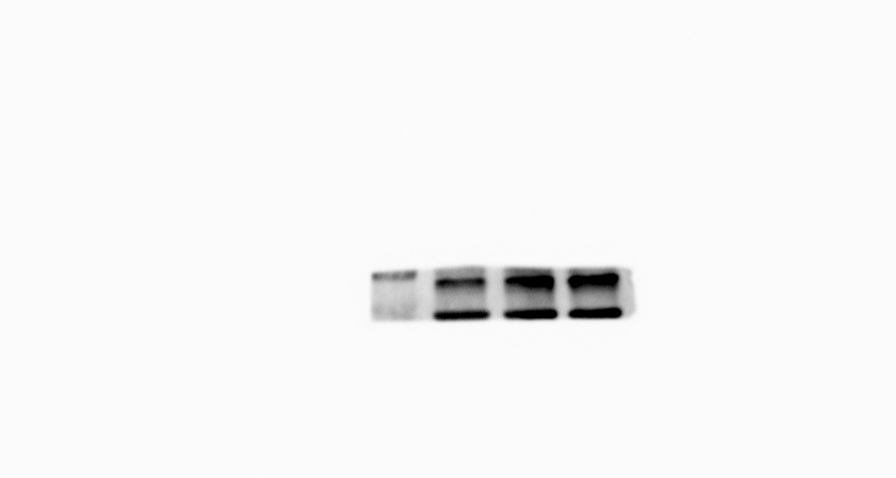


β-catenin


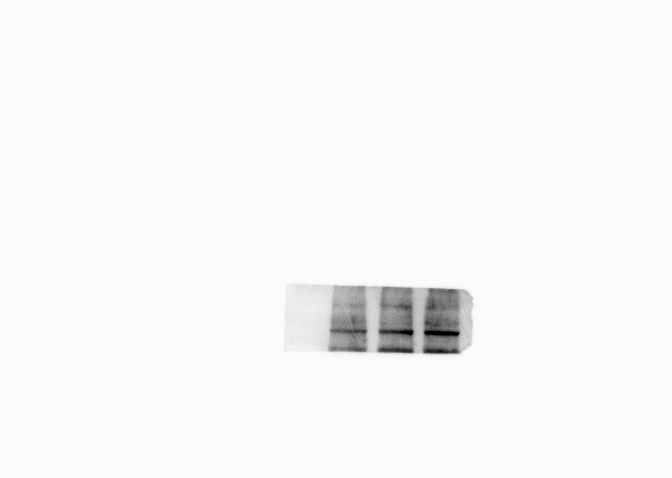


MSAB:

ACTIN


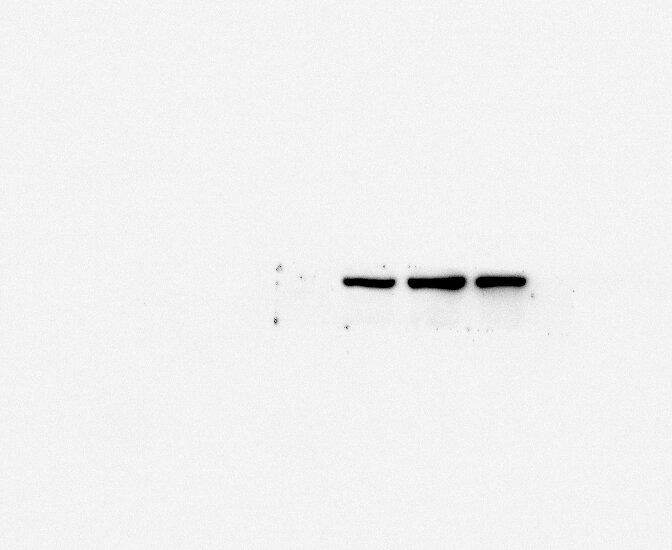


ACVRL1

β-catenin

Fig.6E

ACTIN

ACVRL1

Fig.6G

HCT15

ACTIN

ACVRL1

HCT116

ACTIN

ACVRL1

Fig. 6H

ACTIN

ACVRL1

Fig. S1C

ACTIN

ACVRL1

Fig. S4B

HCT15

ACTIN

ACVRL1

GPX2

LS174T:

ACTIN

ACVRL1

GPX2

Fig. S4C

Input:

ACTIN

GPX2

USP15

IP:

ACVRL1

GPX2

Fig. S4D

Input:

ACTIN

GPX2

USP15

IP:

ACVRL1

USP15

Fig. S5A

ACTIN(Lane1-3)

ACVRL1(Lane1-3)

β-catenin(Lane1-3)
